# Investigations Into Chemically Stabilized Four-Letter DNA for DNA-Encoded Chemistry

**DOI:** 10.3389/fchem.2022.894563

**Published:** 2022-06-09

**Authors:** Marco Potowski, Verena B. K. Kunig, Lukas Eberlein, Mateja Klika Škopić, Alexandros Vakalopoulos, Stefan M. Kast, Andreas Brunschweiger

**Affiliations:** ^1^ Department of Chemistry and Chemical Biology, Medicinal Chemistry, TU Dortmund University, Dortmund, Germany; ^2^ Department of Chemistry and Chemical Biology, Physical Chemistry, TU Dortmund University, Dortmund, Germany; ^3^ Bayer AG, Pharmaceuticals, Research and Development, Synthetic Modalities, Wuppertal, Germany

**Keywords:** DNA-encoded library, DNA-encoded chemistry, chemically modified nucleobase, multi-component reaction, heterocyclic chemistry

## Abstract

DNA-encoded libraries are a prime technology for target-based small molecule screening. Native DNA used as genetic compound barcode is chemically vulnerable under many reaction conditions. DNA barcodes that are composed of pyrimidine nucleobases, 7-deazaadenine, and 7-deaza-8-azaguanine have been investigated for their suitability for encoded chemistry both experimentally and computationally. These four-letter barcodes were readily ligated by T4 ligation, amplifiable by Taq polymerase, and the resultant amplicons were correctly sequenced. Chemical stability profiling showed a superior chemical stability compared to native DNA, though higher susceptibility to depurination than a three-letter code based on pyrimidine DNA and 7-deazaadenine.

## 1 Introduction

**FIGURE 1:**
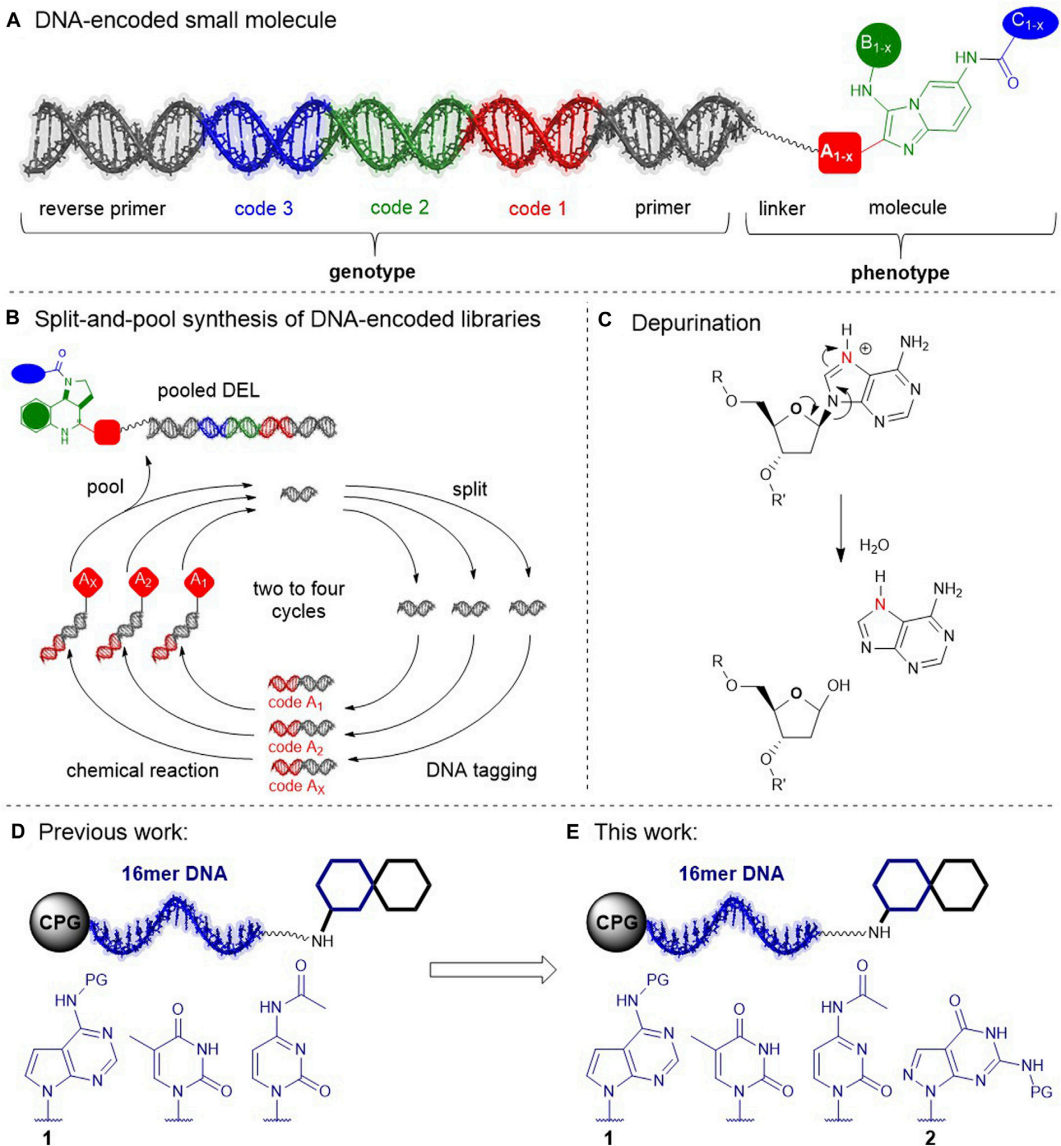
DNA-encoded libraries. **(A)** A DNA-encoded compound. **(B)** Split-and-pool DEL synthesis **(C)** DNA damage by depurination. **(D)** Previous work: Study of a chemically stabilized code consisting of nucleobases T, C, and 7-deazaA **1**. **(E)** This work: From a three-to a four-letter barcode. Investigation of 8a-7-deazaG **2**; PG, protective group: benzoyl (**1**) or DMF (**2**).

DNA-encoded compound libraries (DELs) have evolved to a prominent small molecule screening technology for drug discovery projects (Flood et al., 2020; Gironda-Martínez et al., 2021; Satz et al., 2022). Typically synthesized by split-and-pool combinatorial chemistry routes that go through cycles of iterative DNA-tagging and compound synthesis steps, DELs give efficient access to unsurpassed numbers of compounds for target-based screening (Figures 1A,B). DEL chemical space is biased by stringent demands on chemistry for library design (Franzini and Randolph, 2016; Malone and Paegel, 2016; Fitzgerald and Paegel, 2021): split-pool-compatible building blocks, and robust “click-chemistry”-like reactions that tolerate moisture. Furthermore, the synthesis process demands any DEL reactions to be compatible with the DNA barcode in order to obtain functional libraries that can be read out by sequencing with high fidelity. Reaction conditions need to avoid DNA damage reactions such as depurination (Figure 1C), nucleobase deamination, 8-oxopurine formation, nucleophile addition, thymine dimerization, tautomerization e.g. from metal adducts, and oligomer fragmentation, to name a few notorious reactions that render the barcode unreadable (Gates, 2009). Recently, we demonstrated a compound barcoding strategy that was initiated with controlled pore glass-connected chemically stabilized (cs)DNA barcodes (Potowski et al., 2021). These csDNA barcodes were composed of the pyrimidine nucleobases T and C, and 7-deazaadenine (7De-dA) which replaced the particularly vulnerable purine nucleobases A and G, as demonstrated earlier for encoded solid phase peptide synthesis (Needels et al., 1993) (Figure 1D). The three-letter code was functional as a genetic code, showed remarkable chemical stability to protic acids such as trifluoroacetic acid, as well as to a large number of metal catalysts, and allowed for ready translation of more than a dozen reactions for diverse DEL design, including Boc-chemistry, the Pictet-Spengler reaction and isocyanide multi-component reactions. Here, we investigated expanding the three-letter barcode to a full four-letter barcode, requiring a chemically stabilized 2′-deoxyguanosine analogue to be added to the established dT, dC, 7De-dA-code. The functionality of heavily modified DNA strands has been investigated by e.g. the research groups of Famulok, Herdewijn, and Hocek in the context of aptamer design, synthetic biology, and nucleic acid sequencing, inspiring our research (Jäger et al., 2005; Ondruš et al., 2020; Kodr et al., 2021). We selected 7-deaza-8-aza-2′-deoxyguanine (abbreviated 7De8a-dG) for our studies because the analogous adenine derivative showed high chemical stability previously (Potowski et al., 2021) (Figure 1E), while 7-deaza-2′-deoxyguanine (7De-dG) was not considered for barcoding chemistry because of its known potential to form oxidation products (Yang and Thorp, 2001). The 7De8a-dG nucleoside is a relatively understudied DNA modification, capable of forming canonical DNA duplexes (Seela and Driller, 1988; Seela and Driller, 1989), and stabilizing parallel DNA duplex strands (Seela et al., 2000). It served to project dyes into the major groove of a DNA duplex via linker moieties that were installed in the 7-position (Seela et al., 2009), and to investigate the structure-catalytic activity of the 8–17 DNAzyme (Liu et al., 2013). G-C-rich nucleic acid sequences may form G-quadruplexes, a DNA tertiary structure that was shown to produce sequencing artifacts. Owing to the missing nitrogen in the 7-position, 7De8a-dG-containing DNA oligonucleotides did not form such structures, (Seela and Kröschel, 2003), and were therefore used as probes for single nucleotide polymorphism genotyping of G-C-rich sequences (Belousov et al., 2004). The tolerance of the 5′-triphosphate of 7De8a-dG as a substrate of DNA polymerases was investigated by the Herdewijn group in primer extension assays towards future applications e.g. for aptamer diversification, or in synthetic biology. These revealed that the extension of a primer with the 5′-triphosphate of 7De8a-dG gave the target DNA duplex with very low efficiency, and PCR amplification of a template with a mix of nucleoside triphosphates that included the 5′-triphosphate of 7De8a-dG gave hardly the desired amplicon product (Eremeeva et al., 2017a; Eremeeva et al., 2017b). Thus, the utility of 7De8a-dG to substitute the native dG in a compound-encoding DNA template was unclear at the outset of our investigations. These included ligation of barcodes by T4 ligase, amplification of a template strand that contained this modification with Taq polymerase, and sequencing of the amplicon, as well as chemical stability profiling, and reaction translation.

## Materials and Methods

Please see Supporting information for on-DNA synthesis protocols, and protocols for DNA ligation, PCR amplification, and sequencing.

## Results

### Computational Analysis

**FIGURE 2:**
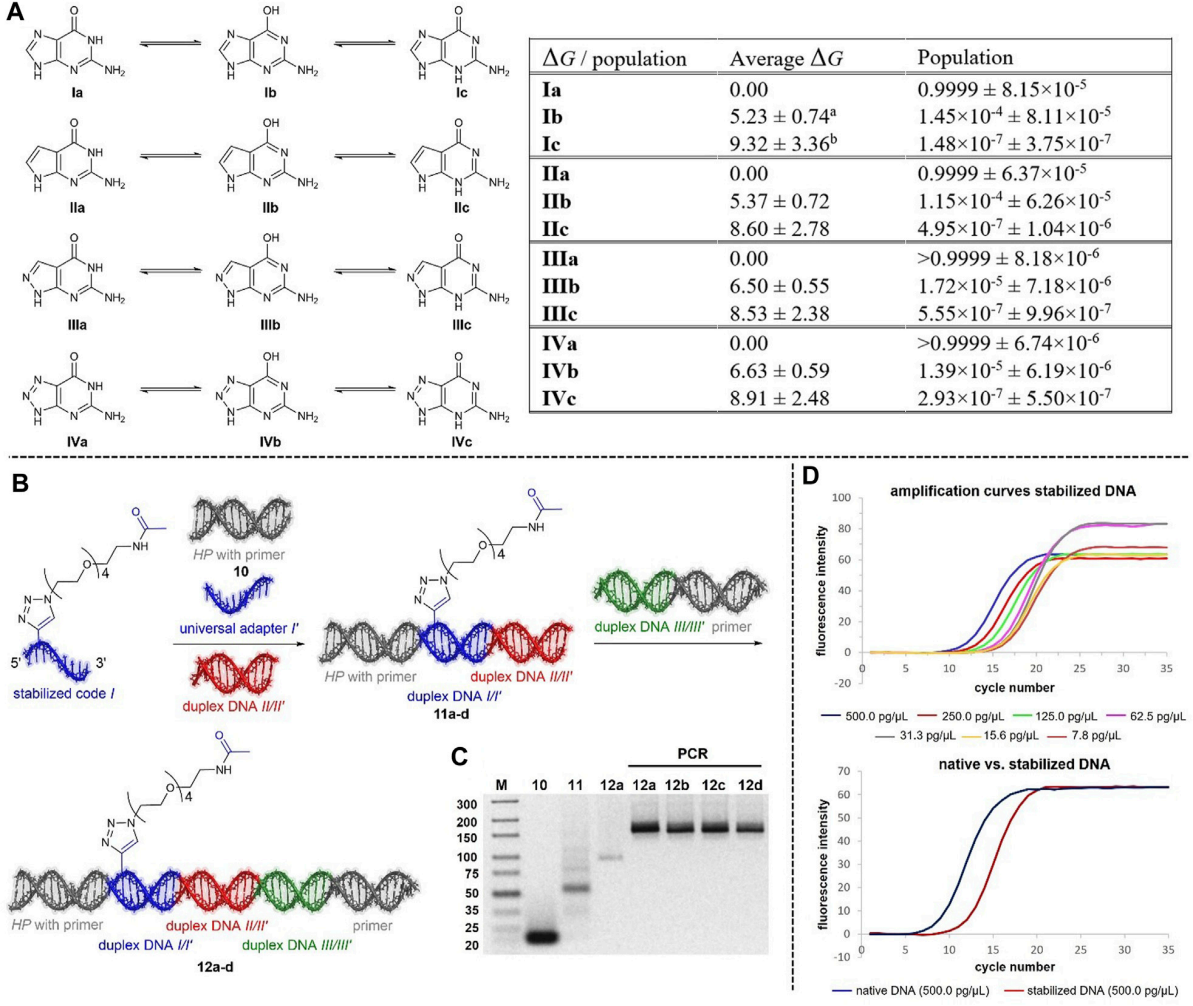
**(A)** Calculated standard reaction free energies Δ*G* (kcalmol^−1^) and populations for selected tautomeric forms of guanine derivatives **I-IV** relative to the Watson-Crick tautomers [**I**-**IVa**] accompanied by uncertainties from averaging over a range of methods^c^. See SI for computational details. ^a^ Eberlein, 2020: 6.6 ± 0.7 kcal mol^−1^, ^b^ Eberlein, 2020: 7.5 ± 1.5 kcal mol^−1^, ^c^ Note that the uncertainties provided in Eberlein, 2020 and Potowski, 2021 were erroneously reported to be too small by a factor of 5^1/2^ = 2.236. Uncertainties are here correct, and the corrected values are also given for reference calculations (Eberlein, 2020) on canonical guanine **I** ^a,b^. This correction has no impact on energetic rankings and discussion of tautomer relevance. **(B)** Encoding strategy using stabilized DNA barcodes. **(C)** Results of the T4 ligation and PCR amplification, ligation carried out with 100 pmol of each oligonucleotide, 600 units of T4 DNA Ligase, gel electrophoresis performed with a 4% agarose gel. **(D)** Amplification efficiencies of a chemically stabilized DNA template and the corresponding native DNA template strand.

The nucleobase tautomer stability was investigated here for guanine derivatives, similar to our investigations of adenine derivatives (Potowski et al., 2021) and the expanded Hachimoji genetic alphabet (Eberlein et al., 2020), since it is a precondition for unambiguously reading the DNA barcode. Applying the same computational methodology as in Potowski et al., 2021, which is a refined protocol compared to our earlier Hachimoji investigations (Eberlein et al., 2020), on other guanine derivatives than presented in this work, we calculated all possible tautomers of guanine **Ia-c**, 7-deazaguanine **IIa-c**, 8-aza-7-deazaguanine **IIIa-c** and 8-azaguanine **IVa-c** (Figure 2A, Supplementary Tables S1-S3) with respect to their thermodynamic stability. According to this analysis, the reaction free energy between the Watson-Crick tautomers (**I-IVa**) and both alternate tautomers (**I-IVb,c**, Figure 2A, Supplementary Tables S1-S3), which translates into tautomer populations, is increased in **II-IVb** and reduced in **II**-**IVc** compared to natural guanine. With the smallest energy difference of more than 5 kcalmol^−1^ being observed for the tautomer **Ib** of the natural guanine, the resulting mismatching tautomer fractions are negligible for all non-natural species as their free energies are even larger. This is a remarkable result as the Hachimoji guanine derivatives are by far less stable from the tautomer perspective (Eberlein et al., 2020). Therefore, 7-deazaguanine, 8-aza-7-deazaguanine or 8-azaguanine containing oligonucleotides should be well suited for DNA encoding chemistry with Watson-Crick tautomer fractions of at least 99.99%. Thus, the choice of a specific nucleobase for encoding chemistry depends on (commercial) nucleoside availability and chemical stability.

### Investigations on Ligation, PCR and Sequencing Using 7De8a-dG-Containing DNA Sequences

Ligation of chemically modified 7De8a-dG-containing DNA barcodes by T4 ligase and correct reading of the resulting DNA template by polymerases are essential for DEL synthesis. We performed test ligations with T4 ligase based on our previously published coding strategy (Supplementry Figures S2B,S1) that uses an adapter oligonucleotide composed of inosine and stable abasic sites opposite the chemically stabilized code, followed by PCR amplification and DNA sequencing. The sequences of all oligonucleotides used in ligation and amplification experiments are given in Supplementary Table S5. Agarose gel electrophoresis using a 3% or 4% agarose gel was used to analyze the ligation experiments (Supplementary Figure S2). Amplicon sequencing of the fully encoded DNA-conjugates showed that the Taq polymerase correctly copied the nucleotides (Figure 2C, Supplementary Table S6). We then compared the amplification efficiency of 7De8a-dG-containing ligation products with one native DNA ligation product by qPCR (Figures 2D, Supplementary Figures S3–S9). Compared to the native DNA, the chemically modified template required a total of two to four cycles more to reach the log-linear phase of amplification. Lower amplification efficiency, especially prior reaching the log-linear phase, may at least partially be explained by the use of a universal adapter sequence opposite the chemically stabilized code. Previously, introduction of abasic sites into DNA templates has been shown to lead to much reduced amplification efficiency (Sikorsky et al., 2007).

### Chemical Stability Screen of Chemically Modified and Native DNA Oligonucleotides

DNA stability under diverse reaction conditions is an essential precondition for the synthesis of functional DELs. For that reason, we recently investigated the stability profile of different CPG-bound pyrimidine DNA oligonucleotides and mixed pyrimidine/purine DNA oligonucleotides in the presence of acidic solutions, diverse metal salts and organic reagents (Table 1) (Potowski et al., 2019). Especially purine containing sequence **3** revealed low stabilities under various conditions e.g. acids or oxidants. Based on these findings and inspired by an early account describing encoded solid-phase peptide chemistry (Needels et al., 1993), we proposed that the substitution of native purine nucleobases in a given sequence by 7-deazaadenine would lead to a three-letter DNA barcode **4** with enhanced chemical stability. Indeed, exchanging 2′-deoxyadenosine with 7De-dA **1** (oligonucleotide **4**) led to a much-improved stability profile which was comparable with the stability of pyrimidine DNA sequences (Potowski et al., 2021). Here, we aimed at going a step further to arrive at a chemically stabilized four-letter DNA barcode **6**. Therefore, we kept the 7De-dA **1** and replaced in addition the native 2′-deoxyguanosine by the modified 7De8a-dG **2**. To our delight, this chemically modified four-letter oligonucleotide **6** showed a higher stability against the tested conditions as compared to the native DNA **3**, too, see Table 1 for the screening results. Especially metal ions were much better tolerated by sequence **six** than by the native DNA **3** (Table 1, entries 2,9,12). However, with the modifications of the 2′-deoxyguanosine we did not reach the same overall level of stability as observed with the previously described 7De-dATC sequence **4**. The stability screen on both 7De8a-dG-containing sequences **5** and **6** revealed that still some DNA degradation occurred under acidic or oxidative conditions. For instance, the incubation of these sequences with a 3.7% aqueous hydrochloric acid solution for 22 h at room temperature led to ca. 50% depurination, while 7De-dA-containing DNA **4** was left completely intact under these conditions, yet, native DNA **3** suffered almost complete degradation by depurination (Table 1, entry 1).

**TABLE 1:** Stability screening of DNA barcodes **5** and **6** in presence of protic acids, metal salts and organic reagents.^a^

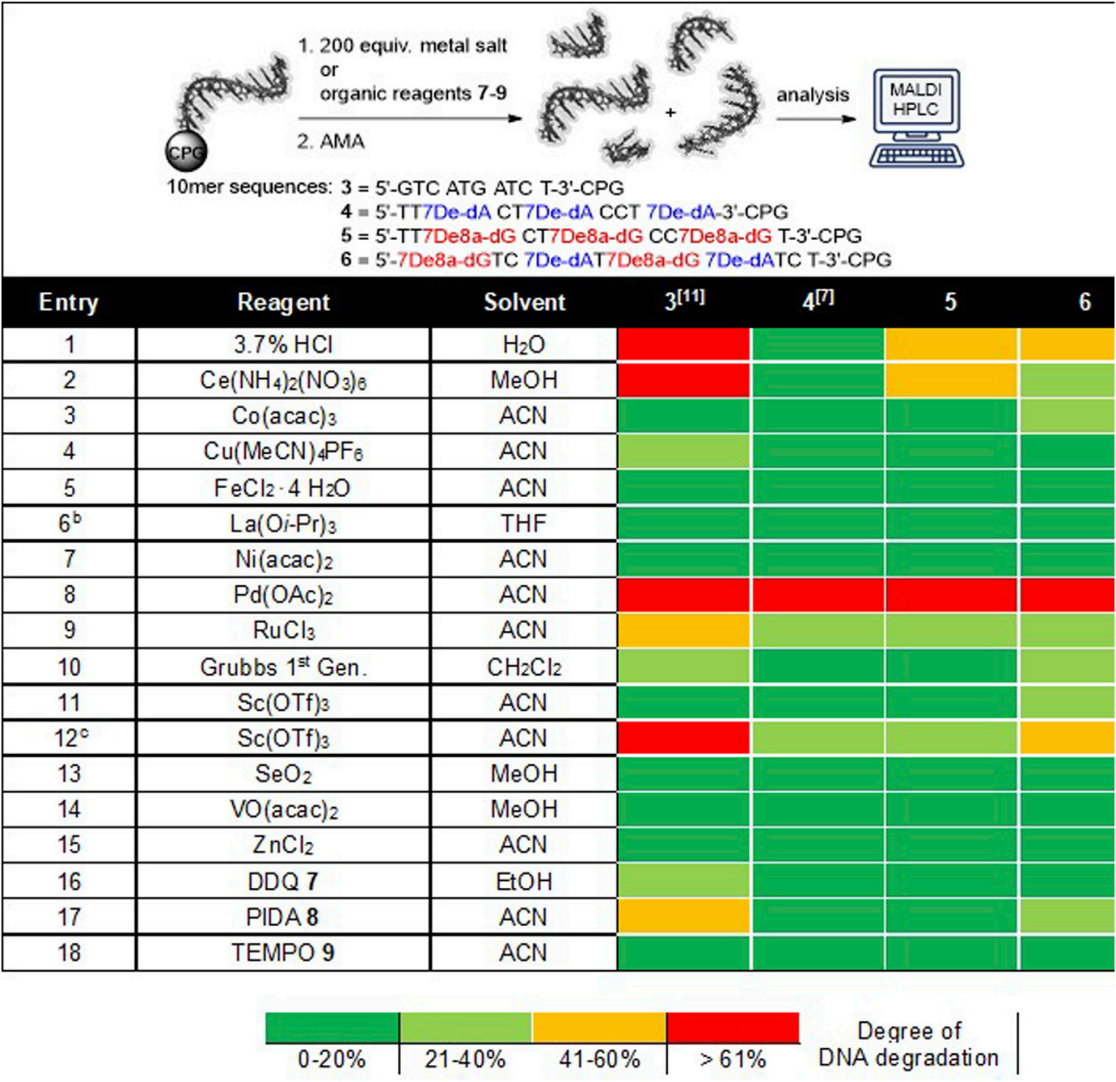							
Entry	Reagent	Solvent	3 [11]	4 [7]	5	6	
1	3.7% HCl	H2O					
2	Ce(NH_4_)_2_(NO_3_)_6_	MeOH					
3	Co(acac)_3_	ACN					
4	Cu(MeCN)_4_PF_6_	ACN					
5	FeCl_2_ ∙ 4 H_2_O	ACN					
6b	La(O*i*-Pr)_3_	THF					
7	Ni(acac)_2_	ACN					
8	Pd(OAc)_2_	ACN					
9	RuCl_3_	ACN					
10	Grubbs 1^st^ Gen	CH_2_Cl_2_					
11	Sc(OTf)_3_	ACN					
12c	Sc(OTf)_3_	ACN					
13	SeO_2_	MeOH					
14	VO(acac)_2_	MeOH					
15	ZnCl_2_	ACN					
16	DDQ **7**	EtOH					
17	PIDA **8**	ACN					
18	TEMPO **9**	ACN					
							
				Degree of DNA degradation			
0–20%	21–40%	41–60%	>61%								
							
							

^a^ For each: 20 nmol DNA, aqueous acids or 200 equiv. transition metal salt or 200 equiv. organic reagent, 50 µL solvent, rt, 22 h ^b^ Poor solubility, added as suspension. ^c^ Experiment was performed at 40 °C. AMA, aq. NH_3_/MeNH_2_, ACN, acetonitrile, MeOH, methanol.

### Translation of Reactions to CPG-Bound Chemically Stabilized ATGC-DNA-Starting Materials

**FIGURE 3:**
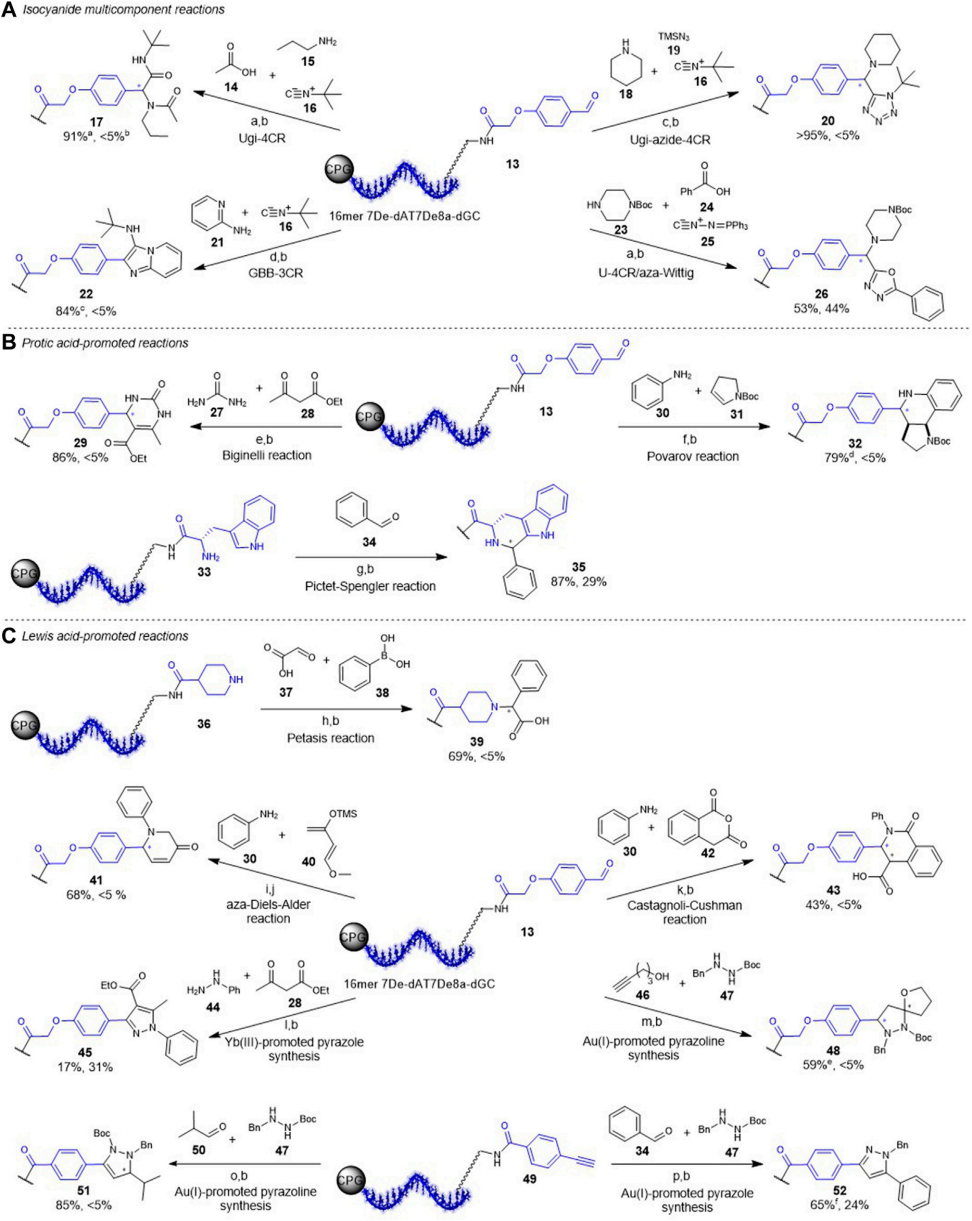
Translation of reactions to CPG-bound DNA-starting materials. **(A)** Isocyanide MCR chemistries; a) MeOH, 50 °C, b) aq. NH3/MeNH2 (AMA), c) MeOH, 50 °C, d) 1% acetic acid/MeOH, 50 °C. **(B)** Protic acid-promoted reactions; e) *(R)*‐(‐)‐BNDHP, EtOH 50 °C, f) *(R)*‐(‐)‐ BNDHP, EtOH/TEOF, 50 °C, g) 5% TFA, CH_2_Cl_2_, rt. **(C)** Metal ion-promoted reactions; h) CuCl/bpy, DMF/TEOF, 50 °C, i) ZnCl_2_, ACN/TEOF, rt, j) aq. NH_3_, 50 °C, 6 h, k) Yb(OTf)_3_, CH_2_Cl_2_/TEOF, rt, l) Yb(PFO)_3_, toluene, 50 °C, m) AuI/AgSbF_6_, THF, rt, n) Ipr AuCl/AgOTf, ACN, 50 °C, o) AuI/AgOTf, glacial acetic acid, 60 °C. p) 10% TFA in H_2_O, 4 h. ^a,b^Conversion and DNA degradation determined by HPLC. ^c^additional 11% of undefined byproduct. ^d^additional 12% of undefined byproduct. ^e^additional 15% of undefined byproducts. ^f^additional 20% of undefined byproducts. AuI=[Tris(2,4‐di‐tert‐butylphenyl)phosphite]-gold chloride. Sequence of all DNA oligonucleotides used in the reactions: CT*C TCT 7De8a‐dGTC T7De8a‐dGT 7De‐dACC T.

Encouraged by the above shown results regarding stability and biological compatibility we started to transfer the previously established reactions on CPG-bound DNA to the chemically stabilized four-letter barcode containing 7De-dA as well as 7De8a-dG (Figure 3, Supplementary Table S7) (Potowski et al., 2021). The first set of reactions belongs to the class of isocyanide multi-component chemistries (Figure 3A). The Ugi four-component reaction (U-4CR), the Ugi-azide four-component reaction (UA-4CR), and the Groebke-Blackburn-Bienaymé three-component reaction (GBB-3CR) were readily performed on a CPG-bound 16mer 7De-dATC7De8a-dG-DNA-encoded aldehyde **13**, resulting in near-quantitative product conversions to target conjugates **17**, **20**, and **22** with less than 5% DNA degradation (Figure 3A). However, translation of the Ugi-aza-Wittig four-component (U-4CR/aza-Wittig) resulted in a high degree of DNA degradation (44%) and a conversion of 53% to the desired product **26** (Figure 3A). Next, we explored protic acid-promoted reactions like the (*R*)-(-)-BNDHP promoted Biginelli reaction (Figure 3B). This reaction proceeded well with urea to the desired product **29** with a high conversion of 86% and no detectable DNA degradation. Notably, also the more challenging phenyl urea that requires higher amounts of the acid worked smoothly without DNA damage (see SI). The (*R*)-(-)-BNDHP promoted Povarov reaction led to the desired product **32**, too. However, in this case a low degree of DNA degradation was observed. The trifluoroacetic acid-promoted Pictet-Spengler reaction allowed for synthesis of the desired β-carboline **35** from a DNA-tryptophane conjugate **33** with 85% conversion. As expected after the stability screen, 40% of the DNA were degraded during the reaction. The intact DNA-Pictet-Spengler conjugate **34** could be isolated by preparative RP-HPLC. Last, we investigated the usability of Lewis acid-promoted reactions on the chemically stabilized barcode (Figure 3C). The Cu(I)/bipyridine-promoted Petasis reaction, the Zn(II)-promoted aza-Diels-Alder reaction as well as the Yb(III)-promoted Castagnoli-Cushman reaction worked smoothly without any DNA degradation and led to the desired DNA-coupled products **39**, **41** and **44** with moderate to good conversions. The Yb(III)-mediated pyrazole synthesis using aryl hydrazines resulted in the desired product **45** with a conversion of 17% and DNA degradation of 31% (Figure 3C). The Au(I)-promoted pyrazoline containing spiroheterocycle **48** synthesis starting from DNA-aldehyde conjugate **13** with alkynol **46** and hydrazide **47** was successful with moderate conversion and no detectable DNA damage. Also, the Au(I)-promoted pyrazoline synthesis from 16mer 7De-dAT7De8a-dGC-alkyne conjugate **49** with isobutyraldehyde **50** and hydrazide **47** led to the desired product **51** with an excellent conversion, and without DNA degradation. However, the more demanding Au(I)-promoted pyrazole synthesis in glacial acetic acid at 60 °C resulted in a higher degree of DNA damage (32%). But still the isolation of intact DNA-pyrazole conjugate **52** was possible by preparative RP-HPLC.

Beside the synthesis of CPG-bound DNA conjugates, the protective group removal in aqueous solution plays an important role in DEL synthesis, too. Therefore, we explored the Boc deprotection of an isolated 16mer 7De-dAT7De8a-dGC-tetrahydroisoquinoline conjugate **32** with 10% TFA in water. However, the reaction conditions led to DNA degradation by exclusive depurination of the 7-deaza-8-aza-2′deoxyguanosine, as analyzed by MALDI MS, while the 7-deazaadenine remained intact. This clear-cut result was in line with the substantial amounts of depurination already observed in the Pictet-Spengler reaction that was performed with 5% of TFA for 20 h in an organic solvent on a CPG-bound and fully nucleobase-protected oligonucleotide.

## Discussion

**FIGURE 4:**
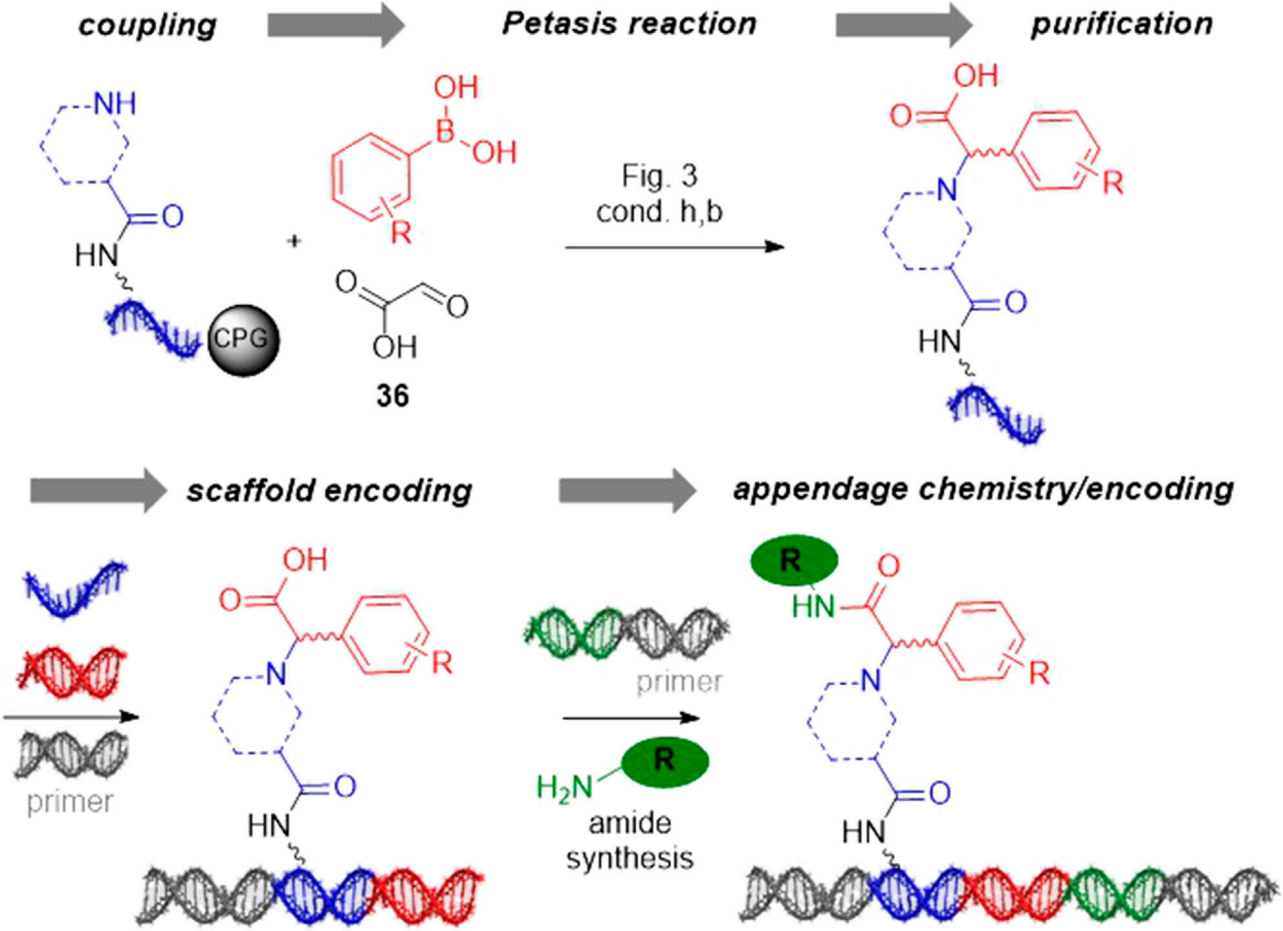
Exemplary DNA-encoded library design based on the Petasis reaction. DEL synthesis is initiated with coupling of secondary amines to the stabilized barcode. Petasis reaction on encoded amines gives for instance diverse N-substituted arylglycines. These are purified, encoded and substituted by amide bond formation in the third reaction step giving a library of diverse N-substituted arylglycine amides.

Here, we investigated the suitability of a chemically modified G, namely 7-deaza-8-aza-2′deoxyguanosine for use in DNA-encoded chemistry. Heavily modified DNA sequences that contained two chemically modified nucleobases are tautomerization-wise stable, could be ligated to other DNA barcodes, and served as functional templates for PCR amplification. Thus, they were suitable for encoded library synthesis. Furthermore, they displayed much increased resistance to chemical degradation by metal ions, and also some level of stability to acid-promoted reaction conditions, though the latter was much less pronounced, as compared to the previously developed three-letter code (Potowski et al., 2021). Synthesis of a pilot library as exemplified by Figure 4 will be shown in due time.

## Data Availability

The original contributions presented in the study are included in the article/Supplementary Material, further inquiries can be directed to the corresponding authors.
