# Supplementary material for: Investigations Into Chemically Stabilized Four-Letter DNA for DNA-Encoded Chemistry
Source: Front Chem. 2022 Jun 9;10:894563. doi: 10.3389/fchem.2022.894563 (PMC9218945; doi:10.3389/fchem.2022.894563)
Supplement: Supplementary file 1 [file DataSheet1.pdf]

Supporting Information

Investigations into chemically stabilized four-letter DNA for DNA-encoded chemistry

Marco Potowski,[1†] Verena B. K. Kunig,[1†] Lukas Eberlein[2], Mateja Klika Škopić[1], Alexandros Vakalopoulos[3], Stefan M. Kast[2], Andreas Brunschweiger[1]

[1]TU Dortmund University, Department of Chemistry and Chemical Biology, Medicinal Chemistry, Otto-Hahn-Str. 6, 44227 Dortmund, Germany

[2]TU Dortmund University, Department of Chemistry and Chemical Biology, Physical Chemistry, Otto-Hahn-Str. 4a, 44227 Dortmund, Germany

[3] Bayer AG, Pharmaceuticals, Research and Development, Synthetic Modalities, Aprather Weg 18a, 42113 Wuppertal, Germany

[†] These authors contributed equally

Table of Contents	Page

General methods and materials S4

Computational details S6
Calculation of tautomer populations S7

Synthesis of chemically stabilized nucleoside phosphoramidites A-C S10
NMR spectra S12

Chemical stability screening of DNA barcodes S15
Representative procedures S15
HPLC traces and MALDI-MS spectra S17

Biological experiments S35
T4 ligation and amplification by PCR S35
Results from Sanger sequencing S38
Results from qPCR S40

DNA-encoded chemistries S43
Representative procedures S43
HPLC traces and MALDI-MS spectra S60
- CPG-bound DNA-starting material conjugates S60
- Isocyanide multicomponent reactions S65
  - Ugi four-component reaction S65
  - Ugi-azide four-component reaction S66
  - Groebke-Blackburn-Bienaymé three-component reaction S67
  - Ugi four-component/aza-Wittig reaction S68
- Brønsted acid-mediated reactions S69
  - Biginelli reaction S69
  - Povarov reaction S71
  - Pictet-Spengler reaction S72
- Lewis acid-promoted reactions S73
  - Petasis reaction S73
  - aza-Diels-Alder reaction S74
  - Castagnoli-Cushman reaction S75
  - Yb(III)-mediated three-component synthesis of pyrazoles S76
  - Au(I)/Ag(I)-promoted pyrazoline-containing spiroheterocycle synthesis S77
  - Au(I)/Ag(I)-promoted pyrazoline synthesis S78
  - Au(I)/Ag(I)-promoted pyrazole synthesis S79
- Boc cleavage in aqueous solution S80

References S81

Supplementary Figures
Figure S1 – Encoding scheme for ligations with chemically stabilized barcodes S37
Figure S2 – Agarose gel S37

Figure S3 – Amplification curves (qPCR) of DNA sequence containing barcode *Ia* S40
Figure S4 – Standard curves (qPCR) of DNA sequence containing barcode *Ia* S40
Figure S5 – Melting curves (qPCR) of DNA sequence containing barcode *Ia* S41
Figure S6 – Amplification and melting curves (qPCR) of DNA sequence containing barcode *Ib* S41
Figure S7 – Amplification and melting curves (qPCR) of DNA sequence containing barcode *Ic* S41
Figure S8 – Amplification and melting curves (qPCR) of DNA sequence containing barcode *Id* S42
Figure S9 – Differences in the amplification rate of different DNA sequences containing stabilized DNA barcodes (*Ia*-*d*) and native DNA (*Ia*) S42

Supplementary Tables

Table S1 – Calculated Δ*G* and populations for selected tautomeric forms of guanine derivatives S7
Table S2 – Results (in kcal mol^{-1} for energies) of EC-RISM and vacuum calculations S8
Table S3 – Results (in kcal mol^{-1}) of PCM (MP2/6-311+G(d,p)) and TI calculations S9
Table S4 – Stability of chemically modified oligonucleotides **5** and **6** S16
Table S5 – Sequences of DNA oligonucleotides *I* – *IV*/*IV'* S35
Table S6 – Sanger sequencing results S38
Table S7 – Overview of diverse chemical reactions on CPG-bound stabilized barcode S57

General methods and materials

Unless otherwise noted, chemicals were purchased from *abcr, Acros Organics, Alfa Aesar, Fisher Scientific, Merck, Sigma Aldrich, TCI* and *VWR* and were used as provided without further purifications. Dry solvents (ACN, CH_2Cl_2, DCE, DMF, EtOH, MeOH, THF, toluene) were used as commercially available.

5'-Aminolinker-modified DNA oligonucleotides on controlled pore glass solid support (CPG, 1000 Å porosity) were synthesized by *IBA* (Göttingen, Germany). The 10mer T7De8a-dGC- and 10mer 7De-dAT7De8a-dGC–oligonucleotides as well as the branched 16mer 7De-dAT7De8a-dGC-alkyne conjugates on controlled pore glass solid support (CPG, 1000 Å porosity) were synthesized by *Ella Biotech GmBH* (Planegg, Germany). DNA hairpin and barcodes for ligation experiments were purchased from *Integrated DNA Technologies* (IDT).

CPG with oligonucleotide-small molecule conjugates were filtered and washed through synthesis columns using a vacuum manifold (Vac-Man®) from *Sigma Aldrich*.

Oligonucleotide concentrations. Concentrations were determined by UV spectroscopy using a NanoDrop 2000 spectrophotometer from *Thermo Fisher Scientific*.

Semi-preparative ion pair RP-HPLC. Compound purification was performed on a *Shimadzu Prominence* HPLC System equipped with a C_{18} stationary phase (*Phenomenex*, Gemini, 5 µm, C_{18}, 110 Å, 100 x 4.6 mm). A gradient from 100 mM aqueous triethylammonium acetate (pH = 8.0, eluent A) to MeOH (eluent B) was used at a flow rate of 5 mL/min. Fractions containing the desired product were pooled and concentrated.

> **Method:** Step gradient of 20% to 70% B within 13 min, then 70% to 100% B within 1 min followed by 100% B for 3 min using 100 mM aqueous triethylammonium acetate (pH = 8.0, eluent A) and MeOH (eluent B) at a flow rate of 5 mL/min.

Analytical RP-HPLC (I). HPLC analysis was performed on an *Agilent* 1100 series chromatograph equipped with 1100 Quaternary Pump (*G1311A*), a 1100 Multi-Wavelength Detector (G1365B) and an *Agilent* Eclipse Plus C_{18} (4.6 x 100 mm, 3.5 µm) column. The conversion and purity of DNA conjugates were determined by integration of peaks recorded at 254 nm wavelength.

> **Method:** Step gradient of 10% to 70% B within 10 min, then 70% to 100% B within 2 min followed by 100% B for 2 min using 10 mM aqueous triethylammonium acetate (pH = 8.0, eluent A) and MeOH (eluent B) at a flow rate of 0.6 mL/min.

Analytical RP-HPLC (II). HPLC analysis was performed on a *Shimadzu Prominence* equipped with an *Agilent* Eclipse Plus C_{18} (4.6 x 100 mm, 3.5 µm) column. The conversion and purity of DNA conjugates were determined by integration of peaks recorded at 254 nm wavelength.

Method: Step gradient of 10% to 60% B within 10 min, then 60% to 100% B within 2 min followed by 100% B for 2 min using 10 mM aqueous triethylammonium acetate (pH = 8.0, eluent A) and MeOH (eluent B) at a flow rate of 0.6 mL/min.

MALDI-TOF. Mass analysis was performed on a MALDI TOF/TOF MS from *Bruker Daltonics* using 2',4',6'-trihydroxyacetophenone (THAP) matrix (*Dichrom*).

^{1}H-NMR and **^{13}C-NMR** Bruker AVANCE 700 spectrometer (^{1}H NMR, 700 MHz; ^{13}C NMR, 176 MHz). Data are reported in the following order: chemical shift (δ) values are reported in ppm with the solvent resonance as internal standard (DMSO-D6: δ = 3.33 ppm for ^{1}H, δ = 39.52 ppm for ^{13}C) or relative to TMS (δ = 0 ppm); multiplicities are indicated s (singlet), d (doublet), t (triplet), q (quartet) m (multiplet); coupling constants (J) are given in Hertz (Hz).

Computational details

The general computational approach is nearly identical to the refined procedure applied in Ref.[1] and was outlined and validated in Ref.[2] . The solution-phase structures were generated by manual construction of an exhaustive set of OH rotamers and optimized at the B3LYP/6-311+G(d,p)/IEFPCM level of theory with the default parameters for water as implemented in Gaussian 16 rev. B.01[3] (used for all calculations unless explicitly stated otherwise). These structures were reoptimized in vacuum using B3LYP/6-311+G(d,p), using frequency calculations to confirm the structures as local minima and providing data for thermal corrections to yield gas-phase free energies. The vacuum-optimized structures were employed in MP2/6-311+G(d,p) calculations for determining the gas-phase leg of the solvation free energy and in CCSD(T)/cc-pVQZ calculations using the ORCA software (version 4.0.1) within the R1-F12 approximations for the gas-phase reaction energy baseline.[4,5,6] The PCM-optimized structures were submitted to MP2/6-311+G(d,p)/IEFPCM, MP2/6-311+G(d,p)/EC-RISM[7,8] for computing the solvation free energy relative to MP2/6-311+G(d,p) in the gas phase, and to rigid-body thermodynamic integration (TI) calculations in order to provide an alternative, molecular dynamics-based approach to the solvation free energy. EC-RISM calculations were performed using the computational setup developed during the SAMPL6 blind prediction challenge[9] (140^3 grid points with 0.3 Å spacing, PSE-2 closure,[10] modified SPC/E water model, GAFF force field (version 1.7)[11,12] with Lorentz−Berthelot mixing rules for Lennard-Jones (LJ) interactions, and exact periodicity-corrected solute-solvent electrostatics) on the MP2/6-311+G(d,p) level of theory in Gaussian 09 rev. E.01.[13] For the TI calculations, 4167 SPC/E[14] water molecules were placed in a 50^3 Å cube around the molecule using packmol 1.1.2.023.[15] The NAMD 2.11[16] software was used for the simulations together with AM1-BCC charges, GAFF 1.7[11,12] parameters for LJ interactions, and a timestep of 2.0 fs. Each setup was minimized followed by 0.4 ns equilibration. The TI coupling parameter λ was scaled equidistantly in steps of 0.1 between 0 and 1 first for the LJ terms using soft-core scaling and afterwards linearly, using the same step size, for the electrostatic interactions, followed by a hysteresis estimation in the reverse order. For each λ step the system was equilibrated for 60 ps simulated for 0.4 ns. Langevin temperature and pressure control was used for setting the temperature to 298.15 K and the pressure to 1 bar. A smooth cutoff switching scheme for LJ interactions between 10 and 12 Å and a 4th order particle mesh Ewald algorithm (1.0 Å grid spacing) for the electrostatic interactions were employed. The water geometry was constrained using the SETTLE algorithm as implemented in NAMD.

Calculation of tautomer populations

The strategy used for the calculation of the tautomer populations of guanine and its derivatives followed closely Refs.[1,2] on the basis of the thermodynamic cycle shown in Figure 4 of Ref.[2] There are multiple routes to calculate the reaction free energies, two "direct" routes were the free energy differences of the species in solution only are considered (PCM and EC-RISM), and three "indirect" routes were the solvation free energies per species are calculated (PCM, EC-RISM, TI), and the cycle is completed with the gas-phase reaction free energies taken from CCSD(T) calculations including thermal corrections on the B3LYP/6-311+G(d,p) level. The results for the guanine, 7-deazaguanine, 8-aza-7-deazaguanine and 8-azaguanine tautomers are presented in Table S1, all referenced to the Watson-Crick tautomer. Since all five approaches revealed similar trends, the average reaction free energies and resulting tautomer populations over all methods were calculated. The individual free energy components are given in Table S2 and S3; the structures are provided in machine-readable format in the accompanying zip file. As also mentioned in the main text, the uncertainties provided in Refs.[1,2] were erroneously reported to be too small by a factor of $5^{1/2}$ = 2.236. Uncertainties are here correct, and the corrected values are also given for reference calculations[2] on canonical guanine **I**. This correction has no impact on energetic rankings and discussion of tautomer relevance.

Table S1 – Calculated standard reaction free energies Δ*G* (kcal mol^{-1}) and populations for selected tautomeric forms of guanine derivatives **I-IV** relative to the Watson-Crick tautomers [**I-IVa**] from direct MP2/6-311+G(d,p)/PCM calculations (column 2), MP2/6-311+G(d,p)/PCM hydration free energy differences and CCSD(T)/cc-pVTZ gas phase reaction free energies (column 3), direct MP2/6-311+G(d,p)/EC-RISM (column 4), MP2/6-311+G(d,p)/EC-RISM hydration free energy differences and CCSD(T)/cc-pVTZ gas phase reaction free energies (column 5), and from TI hydration free energy differences and CCSD(T)/cc-pVTZ gas phase reaction free energy (column 6); resulting averaged free energies and populations are shown in columns 7 and 8. The averages from Ref.[2] are presented in columns 9 and 10 and the ones calculated without using the TI results in columns 11 and 12.

Cmpd.	PCM	PCM CCSD(T)	EC-RISM	EC-RISM CCSD(T)	TI CCSD(T)	Average Δ*G*	Population	Average Δ*G*[2]	Population[2]	Average Δ*G* (without TI)	Population (without TI)
**Ia**	0.00	0.00	0.00	0.00	0.00	0.00	0.9999 ± $1.82 \cdot 10^{-4}$	0.0	>0.9999 ± <0.0001	0.0	0.9999 ± $1.45 \cdot 10^{-4}$
**Ib**	4.72	4.58	6.20	6.07	4.60	5.23 ± 0.74	$1.45 \cdot 10^{-4}$ ± $1.81 \cdot 10^{-4}$	6.6 ± 0.7	<0.0001 ± <0.0001	5.39 ± 0.75	0.0001 ± $1.40 \cdot 10^{-4}$
**Ic**	9.91	8.65	6.95	5.69	15.39	9.32 ± 3.36	$1.48 \cdot 10^{-7}$ ± $8.39 \cdot 10^{-7}$	7.5 ± 1.5	<10^{-5} ± <10^{-5}	7.80 ± 1.61	$1.92 \cdot 10^{-6}$ ± $5.20 \cdot 10^{-6}$
**IIa**	0.00	0.00	0.00	0.00	0.00	0.00	0.9999 ± $1.42 \cdot 10^{-4}$	-	-	0.0	0.9998 ± $1.91 \cdot 10^{-4}$
**IIb**	4.52	4.50	5.85	5.75	6.24	5.37 ± 0.72	$1.15 \cdot 10^{-4}$ ± $1.40 \cdot 10^{-4}$	-	-	5.16 ± 0.65	0.0002 ± $1.81 \cdot 10^{-4}$
**IIc**	9.62	8.34	6.52	5.24	13.29	8.60 ± 2.78	$4.95 \cdot 10^{-7}$ ± $2.33 \cdot 10^{-6}$	-	-	7.43 ± 1.68	$3.58 \cdot 10^{-6}$ ± $1.02 \cdot 10^{-5}$
**IIIa**	0.00	0.00	0.00	0.00	0.00	0.00	>0.9999 ± $1.83 \cdot 10^{-5}$	-	-	0.0	>0.9999 ± $2.70 \cdot 10^{-5}$
**IIIb**	6.02	5.69	7.19	6.80	6.81	6.50 ± 0.55	$1.72 \cdot 10^{-5}$ ± $1.61 \cdot 10^{-5}$	-	-	6.42 ± 0.60	$1.95 \cdot 10^{-5}$ ± $1.96 \cdot 10^{-5}$
**IIIc**	9.44	8.36	6.72	5.64	12.51	8.53 ± 2.34	$5.55 \cdot 10^{-7}$ ± $2.23 \cdot 10^{-6}$	-	-	7.54 ± 1.46	$2.97 \cdot 10^{-6}$ ± $7.32 \cdot 10^{-6}$
**IVa**	0.00	0.00	0.00	0.00	0.00	0.00	>0.9999 ± $1.51 \cdot 10^{-5}$	-	-	0.0	>0.9999 ± $1.66 \cdot 10^{-5}$
**IVb**	6.59	5.85	7.60	6.86	6.23	6.63 ± 0.59	$1.39 \cdot 10^{-5}$ ± $1.38 \cdot 10^{-5}$	-	-	6.73 ± 0.62	$1.18 \cdot 10^{-5}$ ± $1.24 \cdot 10^{-5}$
**IVc**	9.65	8.52	7.15	6.01	13.23	8.91 ± 2.48	$2.93 \cdot 10^{-7}$ ± $1.23 \cdot 10^{-6}$	-	-	7.83 ± 1.37	$1.81 \cdot 10^{-6}$ ± $4.21 \cdot 10^{-6}$

Table S2 – Results (in kcal mol^{-1} for energies) of EC-RISM and vacuum calculations. Electronic solute energy (E_{sol}, column 2), corrected and uncorrected excess chemical potential (μ^{ex}, $\mu^{ex,corr}$, columns 3 and 5), infinite dilution partial molar volume (V_m in Å^3, column 4), vacuum energies (MP2/6-311+G(d,p) and RI-F12-CCSD(T)/cc-pVTZ results, columns 6 and 7), and thermal corrections (B3LYP/6-311+G(d,p), column 8). “b1” and “b2” denote different OH rotamers, the lines “b” without index show results from a discrete partition function which enter the full reaction free energy. Physically unreasonable partition function estimates are left blank.

Cmpd.	E_{sol}	μ^{ex}	V_m	$\mu^{ex,corr}$	E_{vac}(MP2/ 6-311+G(d,p))	E_{vac}(CCSD(T)/ cc-pVTZ)	TC(B3LYP/ 6-311+G(d,p))	E_{sol}+$\mu^{ex,corr}$
**Ia**	-339755.88	-41.25	115.33	-53.03	-339781.01	-340022.99	52.22	-339808.92
**Ib,1**	-339766.68	-23.95	117.46	-35.95	-339780.89	-340023.04	52.32	-339802.63
**Ib,2**	-339766.24	-23.31	117.47	-35.31	-339780.29	-340022.71	52.29	-339801.55
**Ib**	-	-	-	-	-	-	-	-339802.72
**Ic**	-339717.64	-72.98	111.10	-84.33	-339762.22	-340004.61	51.38	-339801.97
**IIa**	-329703.15	-25.98	130.07	-39.27	-329720.81	-329958.48	59.42	-329742.42
**IIb,1**	-329710.85	-12.15	132.41	-25.68	-329719.99	-329957.67	59.46	-329736.53
**IIb,2**	-329702.80	-18.68	132.10	-32.17	-329715.14	-329953.39	59.09	-329734.98
**IIb**	-	-	-	-	-	-	-	-329736.57
**IIc**	-329668.10	-54.90	126.26	-67.80	-329703.09	-329941.20	58.58	-329735.90
**IIIa**	-339751.41	-29.24	117.80	-41.27	-339770.07	-340012.90	52.28	-339792.68
**IIIb,1**	-339756.84	-16.36	120.00	-28.62	-339768.02	-340011.08	52.21	-339785.47
**IIIb,2**	-339748.69	-22.88	118.63	-35.00	-339762.97	-340006.55	51.81	-339783.69
**IIIb**	-	-	-	-	-	-	-	-339785.50
**IIIc**	-339719.71	-54.64	113.53	-66.24	-339753.11	-339996.25	51.50	-339785.96
**IVa**	-349795.99	-34.69	108.59	-45.79	-349814.07	-350061.10	44.41	-349841.78
**IVb,1**	-349803.64	-19.20	110.56	-30.49	-349812.35	-350059.93	44.28	-349834.14
**IVb,1**	-349802.29	-19.12	109.91	-30.35	-349811.40	-350059.08	43.99	-349832.64
**IVb**	-	-	-	-	-	-	-	-349834.18
**IVc**	-349760.57	-63.45	103.88	-74.06	-349796.28	-350043.61	43.58	-349834.63

Table S3 – *Results (in kcal mol^{-1}) of PCM (MP2/6-311+G(d,p), column 2) and TI (column 5) calculations. Additionally, the solvation free energies* $\Delta_{solv}G$ *calculated using MP2 vacuum energies (columns 3 and 4), the sum of solvation free energies, CCSD(T) and TC (columns 6-8), and the free energies relative to the Watson-Crick tautomer [**I-IVa**] (columns 9-13) are given. "b1" and "b2" denote different OH rotamers, the lines "b" without index show results from a discrete partition function which enter the full reaction free energy. Physically unreasonable partition function estimates are left blank.*

Cmpd.	PCM	$\Delta_{solv}G$ PCM	$\Delta_{solv}G$ EC-RISM	$\Delta_{solv}G$ TI	PCM+ CCSD(T)	EC-RISM+ CCSD(T)	TI+ CCSD(T)	$\Delta\Delta_{solv}G$ PCM	$\Delta\Delta_{solv}G$ PCM+ CCSD(T)	$\Delta\Delta_{solv}G$ EC-RISM	$\Delta\Delta_{solv}G$ EC-RISM+ CCSD(T)	$\Delta\Delta_{solv}G$ TI+ CCSD(T)
**Ia**	-339796.76	-15.75	-27.91	-25.30±0.22	-339986.53	-339998.69	-340048.29	0.00	0.00	0.00	0.00	0.00
**Ib,1**	-339791.93	-11.04	-21.74	-20.34±0.22	-339981.77	-339992.47	-340043.38	-	-	-	-	-
**Ib,2**	-339791.00	-10.72	-21.27	-20.44±0.23	-339981.14	-339991.69	-340043.15	-	-	-	-	-
**Ib**	-339792.04	-	-	-	-339981.94	-339992.61	-340043.69	4.72	4.58	6.20	6.07	4.60
**Ic**	-339786.85	-24.64	-39.75	-28.29±0.24	-339977.87	-339992.99	-340032.91	9.91	8.65	6.95	5.69	15.39
**IIa**	-329732.55	-11.73	-21.61	-21.09±0.21	-329910.80	-329920.67	-329979.58	0.00	0.00	0.00	0.00	0.00
**IIb,1**	-329728.01	-8.01	-16.53	-15.46±0.22	-329906.22	-329914.74	-329973.12	-	-	-	-	-
**IIb,2**	-329725.89	-10.76	-19.84	-19.25±0.23	-329905.05	-329914.13	-329972.64	-	-	-	-	-
**IIb**	-329728.02	-	-	-	-329906.30	-329914.92	-329973.34	4.52	4.50	5.85	5.75	6.24
**IIc**	-329722.92	-19.84	-32.82	-25.09±0.24	-329902.46	-329915.44	-329966.29	9.62	8.34	6.52	5.24	13.29
**IIIa**	-339781.98	-11.91	-22.62	-21.90±0.23	-339972.54	-339983.24	-340034.80	0.00	0.00	0.00	0.00	0.00
**IIIb,1**	-339775.95	-7.93	-17.45	-16.43±0.23	-339966.80	-339976.32	-340027.51	-	-	-	-	-
**IIIb,2**	-339773.49	-10.52	-20.72	-21.09±0.24	-339965.26	-339975.46	-340027.64	-	-	-	-	-
**IIIb**	-339775.96	-	-	-	-339966.85	-339976.44	-340027.99	6.02	5.69	7.19	6.80	6.81
**IIIc**	-339772.54	-19.43	-32.85	-26.04±0.23	-339964.18	-339977.60	-340022.29	9.44	8.36	6.72	5.64	12.51
**IVa**	-349830.00	-15.93	-27.70	-24.29±0.24	-350032.61	-350044.38	-350085.38	0.00	0.00	0.00	0.00	0.00
**IVb,1**	-349823.37	-11.02	-21.79	-18.46±0.24	-350026.68	-350037.44	-350078.39	-	-	-	-	-
**IVb,2**	-349821.84	-10.44	-21.24	-19.88±0.22	-350025.53	-350036.32	-350078.95	-	-	-	-	-
**IVb**	-349823.41	-	-	-	-350026.76	-350037.53	-350079.15	6.59	5.85	7.60	6.86	6.23
**IVc**	-349820.35	-24.07	-38.34	-28.54±0.21	-350024.09	-350038.37	-350072.15	9.65	8.52	7.15	6.01	13.23

Synthesis of chemically stabilized nucleoside phosphoramidite A-C

The synthesis of nucleoside N^6-Benzoyl-2'-deoxy-5'-O-DMT-7-deaza-2'-deoxyadenosine 3'-CE phosphoramidite **A** followed the published procedure.[1] ^{1}H, ^{13}C and ^{31}P NMR spectra are consistent with the published data.[1] The synthesis of nucleoside **B** followed the published procedure.[17]

Synthesis of *N*^{2}-DMF-2'-deoxy-5'-O-DMT-7-deaza-8-aza-2'-deoxyguanosine B

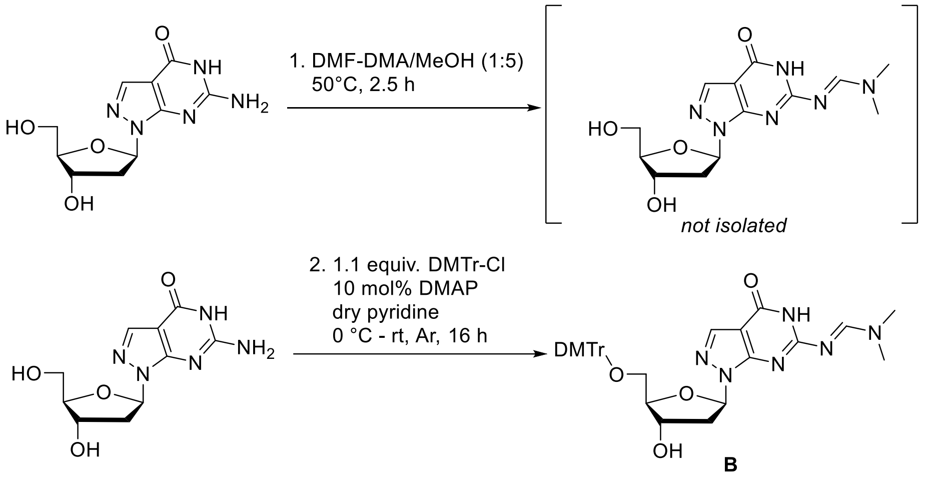

7-deaza-8-aza-2'-deoxyguanosine was dried in high vacuum overnight before setting up the reaction. Step 1: The solution of 7-deaza-8-aza-2'-deoxyguanosine (300 mg, 1.12 mmol, 1.0 eq.) in dry methanol (6 mL) and DMF-DMA (DMF-dimethyl acetale, 1.2 mL) was stirred at 50°C for 2.5 hours. Then, the reaction mixture was concentrated under vacuo, and the crude material was co-evaporated twice with each 3 mL dry methanol and 3 mL diethyl ether, dried under vacuum, and immediately used in the next step without further purification.

Step 2: To the solution of *N*^{6}-DMF-2'-deoxy-7-deaza-8-azaguanosine (355 mg, 1.10 mmol, 1.0 eq.) in dry pyridine (4 mL) at 0°C, DMAP (13 mg, 0.11 mmol, 0.1 eq.) and DMTr-Cl (4,4´-dimethoxytrityl chloride, 411 mg, 1.21 mmol, 1.1 eq.) were added under argon. The reaction mixture was stirred at room temperature overnight. Then, the reaction mixture was concentrated under reduced pressure. To the crude material, CH_2Cl_2 (60 mL) was added and then it was washed with ice-cold brine (3 x 30 mL) and ice-cold water (30 mL). The organic layer was dried over anhydrous Na_2SO_4, filtered, and concentrated *in vacuo*. The crude material was purified by column chromatography (silica gel, solvent system: CH_2Cl_2/ MeOH 100:0 to 90:10) to provide the compound **B** (550 mg, 80% yield). ^{1}H NMR (700 MHz, DMSO): δ = 8.71 (s, 1H), 7.84 – 7.81 (m, 1H), 7.34 – 7.29 (m, 2H), 7.23 – 7.14 (m, 7H), 6.81 – 6.73 (m, 4H), 6.51 – 6.47 (m, 1H), 5.28 (d, J = 4.8 Hz, 1H), 4.52 – 4.46 (m, 1H), 3.91 – 3.87

(m, 1H), 3.70 (d, J = 4.8 Hz, 6H), 3.18 (s, 3H), 3.05 (s, 3H), 3.05 – 2.99 (m, 2H), 2.71 – 2.65 (m, 1H), 2.27 – 2.21 ppm (m, 1H).

^{13}C NMR (176 MHz, DMSO): δ = 159.04, 158.59, 158.53, 157.96, 157.91, 154.52, 145.04, 135.68, 135.67, 135.05, 129.70, 129.63, 127.71, 127.64, 126.50, 113.03, 112.99, 102.24, 85.23, 85.21, 82.91, 70.88, 64.51, 54.97, 54.95, 54.91, 40.80, 40.02, 38.26, 34.81 ppm.

Synthesis of *N*^{2}-DMF-2'-deoxy-5'-O-DMT-2'-7-deaza-8-aza-2'-deoxyadenosine 3'-CE phosphoramidite C

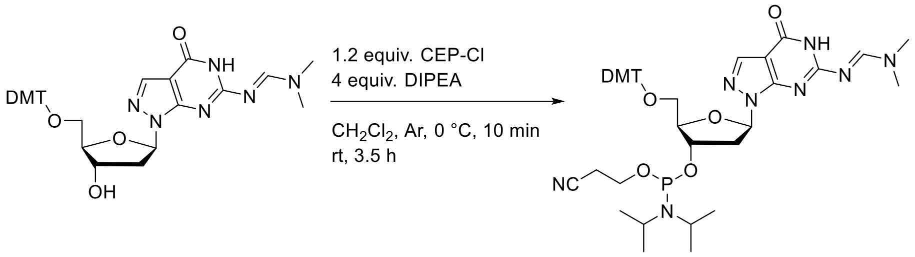

B **C**

N^6-DMF-2'-deoxy-5'-O-DMT-2'-7-deaza-8-aza-2'-deoxyguanosine **B** was dried in high vacuum overnight before setting up the reaction. To the solution of compound **B** (350 mg, 0.56 mmol, 1.0 eq.) in dry CH_2Cl_2 (5.5 mL) at 0°C, DIPEA (390 µL, 2.24 mmol, 4.0 eq.) and CEP-Cl (150 µL, 0.67 mmol, 1.2 eq.) were added under argon. The cooling bath was removed after 10 minutes, and the solution was stirred at room temperature for 3.5 hours. The solution was filtered through a stringe filter and diluted with CH_2Cl_2 (10 mL). The organic phase was washed with saturated aq. $NaHCO_3$ (2 x 20 mL) and brine (20 mL), then dried over anhydrous Na_2SO_4, filtered, and concentrated *in vacuo*. The product **C** was obtained as a colorless foam and as a diastereoisomeric mixture (448 mg, 97% yield). It was used without further purification for solid-phase oligonucleotide synthesis. ^{1}H NMR (700 MHz, DMSO): δ = 8.72 – 8.68 (m, 2H), 7.84 – 7.81 (m, 2H), 7.34 – 7.28 (m, 4H), 7.22 – 7.13 (m, 14H), 6.81 – 6.72 (m, 8H), 6.54 – 6.46 (m, 2H), 4.84 – 4.75 (m, 2H), 4.05 – 3.98 (m, 2H), 3.76 – 3.72 (m, 1H), 3.72 – 3.67 (m, 12H), 3.61 – 3.44 (m, 6H), 3.40 – 3.35 (m, 2H), 3.19 – 3.16 (m, 6H), 3.16 – 3.07 (m, 2H), 3.05 (s, 6H), 3.04 – 2.98 (m, 2H), 2.89 – 2.78 (m, 2H), 2.76 – 2.73 (m, 2H), 2.64 – 2.60 (m, 2H), 2.47 – 2.36 (m, 2H), 1.22 – 1.13 (m, 6H), 1.13 – 1.06 (m, 18H), 0.99 – 0.95 ppm (m, 6H). ^{13}C NMR (176 MHz, DMSO): δ = 159.12, 159.10, 158.57, 158.53, 158.00, 157.96, 154.59, 154.55, 144.92, 135.67, 135.60, 135.57, 135.53, 135.51, 135.22, 129.67, 129.63, 129.56, 127.71, 127.64, 126.54, 118.94, 118.73, 113.00, 112.97, 102.35, 102.33, 102.24, 85.35, 85.32, 85.21, 84.22, 83.99, 83.03, 82.99, 73.51, 73.40, 72.86, 72.76, 64.92, 64.00, 63.78, 58.54, 58.42, 58.31, 54.98, 54.97, 54.95, 42.63, 42.59, 42.56, 42.52, 40.81, 40.02, 34.81, 24.36, 24.32, 24.28, 24.27, 24.23, 24.18, 22.62, 19.83, 19.79, 19.76, 19.72, 15.16 ppm. ^{31}P NMR (283 MHz, DMSO): δ = 147.97, 147.23 ppm.

NMR spectra

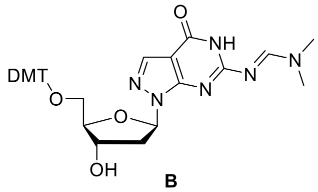

B

^{1}H NMR

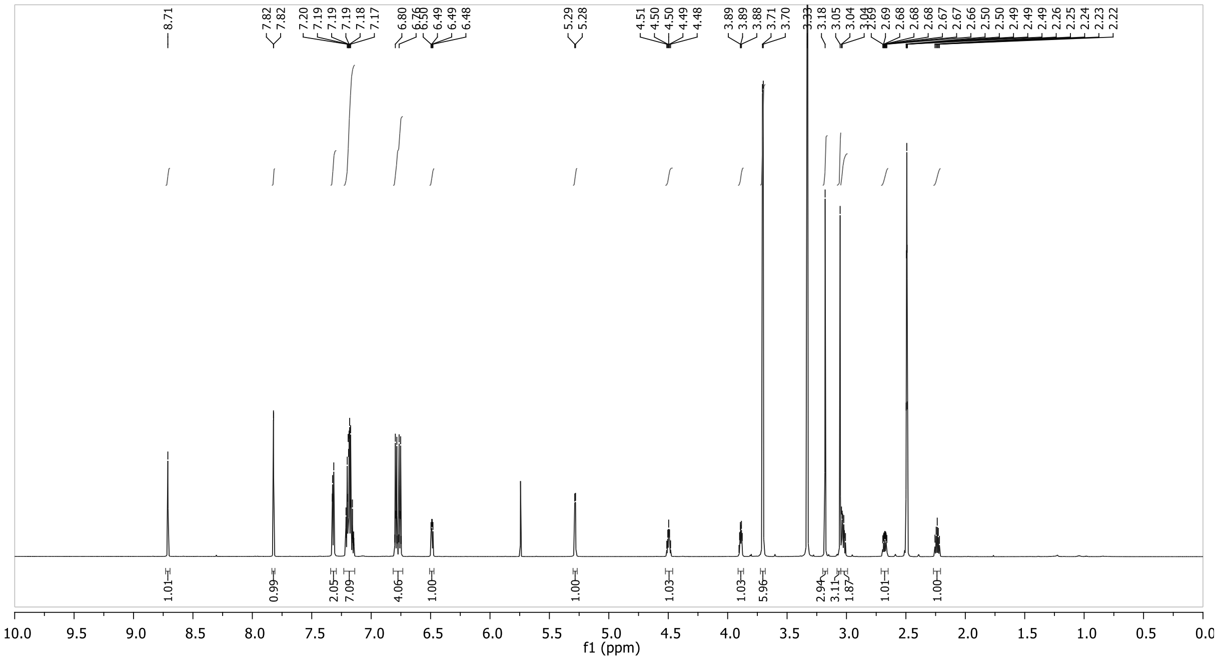

^{13}C NMR

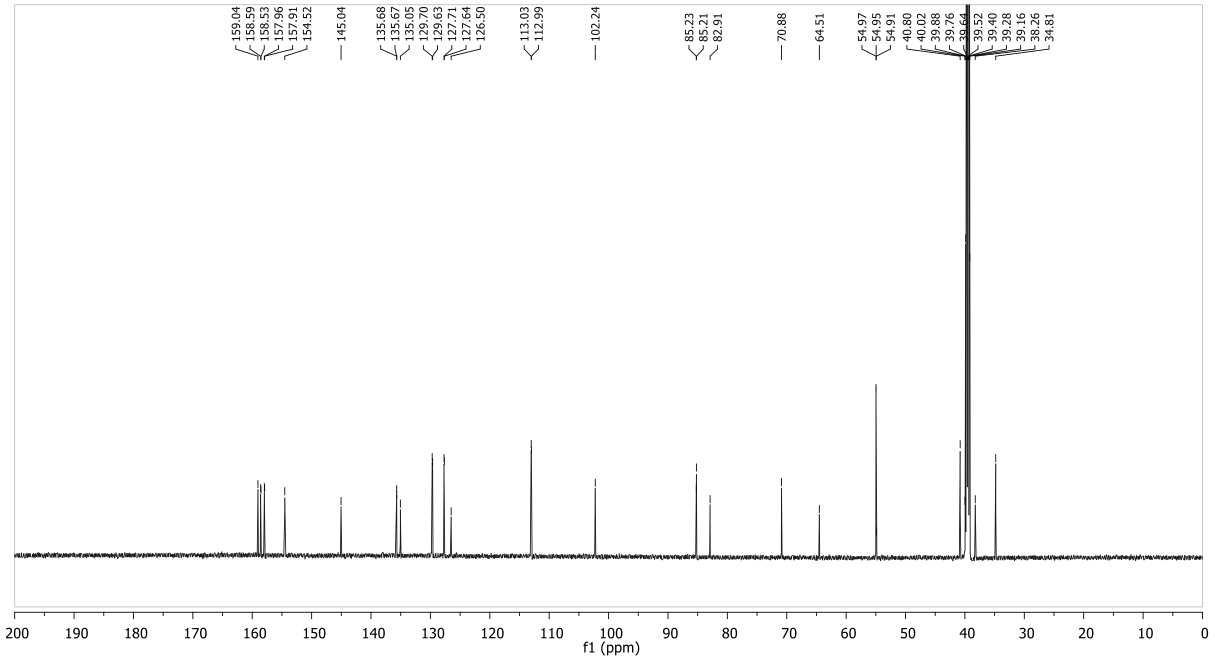

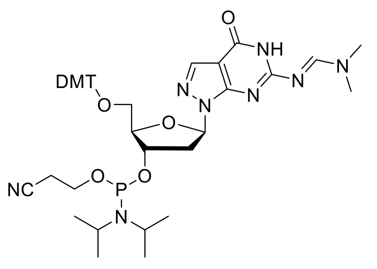

C

^{1}H NMR

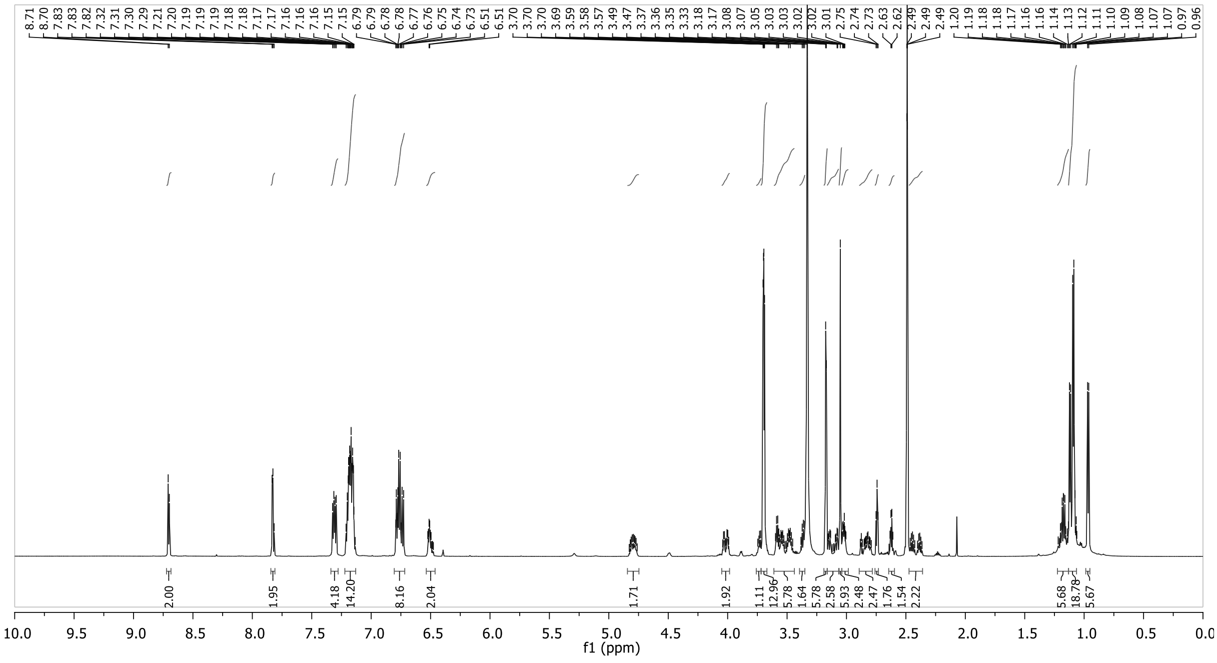

^{13}C NMR

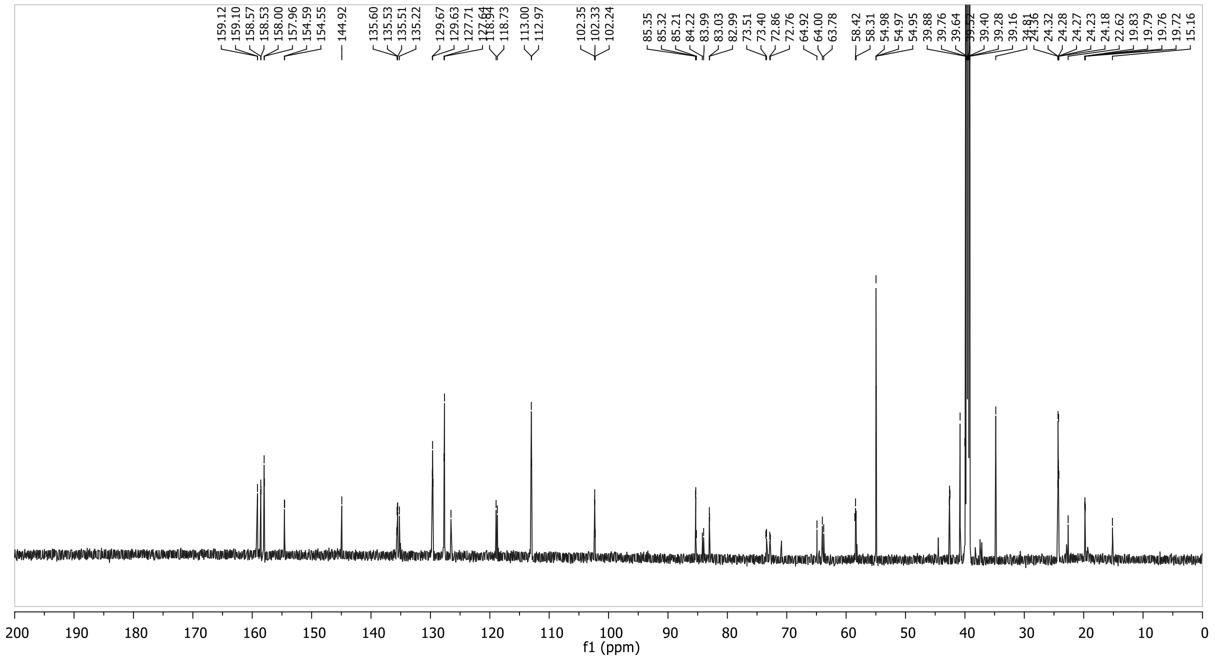

^{31}P NMR

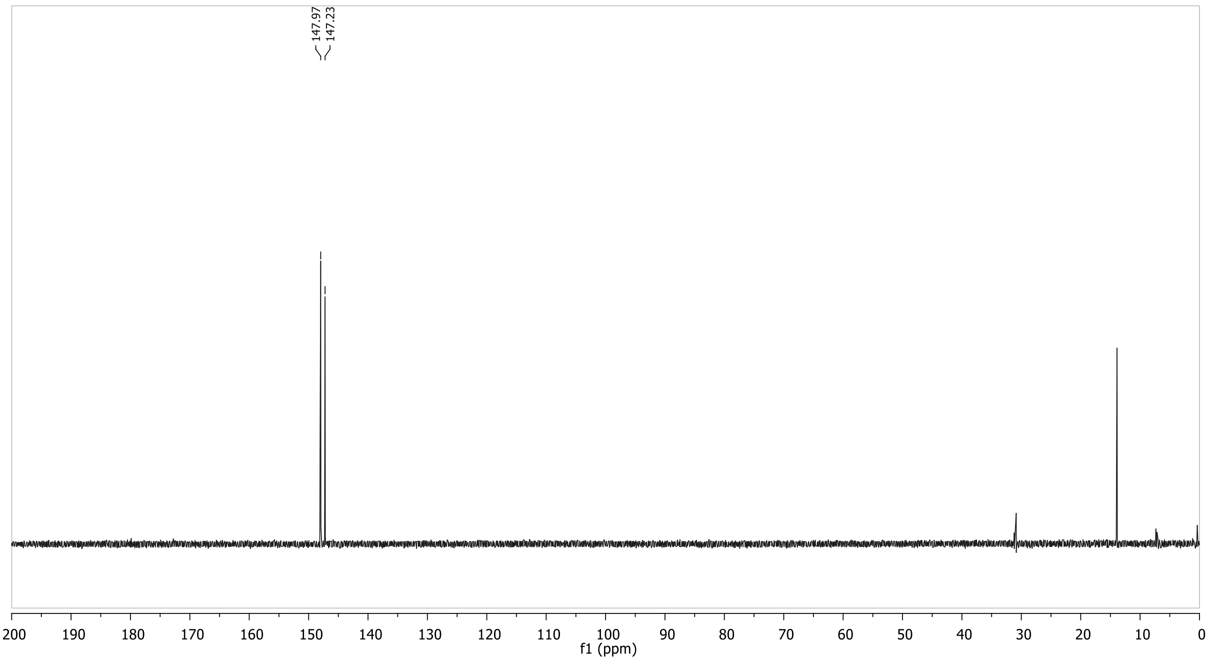
147.97
147.23
200 190 180 170 160 150 140 130 120 110 100 90 80 70 60 50 40 30 20 10 0
f1 (ppm)

Chemical stability screening of DNA barcodes

Representative Procedures

Treatment of solid support-bound stabilized oligonucleotide with aqueous acids (RP-01)

DMT-cleavage: The DMT-protecting group of DNA strand bound to 1000 Å controlled pore glass (CPG) solid support (1 µmol, ~40 mg of 10mer T7De8a-dGC- and 10mer 7De-dAT7De8a-dGC -sequence **5** and **6**) was cleaved by addition of 200 µL 3% trichloroacetic acid in CH_2Cl_2 for 1 min. Orange coloring of the solution indicated successful removal of protecting group. The deprotection was repeated 3-5 times until no further coloring of the solution was observed. CPG-bound deprotected DNA was washed three times with each 200 µL of 1% TEA in ACN, DMF, MeOH, ACN and CH_2Cl_2 and dried *in vacuo*.

Investigation of stability: 20 nmol of CPG-bound stabilized oligonucleotide (0.75 mg) was treated with 50 µL aqueous acid. The suspension was shaken at ambient temperature for 22 h. Afterwards solution was removed under vacuum filtration, CPG was washed with excess of 1% TEA and three times with each 200 µL of 0.1 M $MgCl_2$ solution, water, DMF, MeOH, ACN and CH_2Cl_2 and dried *in vacuo*.

Cleavage and Analysis: DNA was deprotected and cleaved from CPG by shaking in 500 µL of an AMA solution (AMA = aqueous ammonia (30%)/ aqueous methylamine (40%), 1:1, vol/vol) for 4 h at room temperature. Afterwards 20 µL of 1 M Tris buffer (pH = 7.5) were added, the mixture was dried under reduced pressure (SpeedVac) and dissolved in 200 µL of distilled water. The product was analyzed by analytical RP-HPLC and MALDI-TOF-MS.

Treatment of solid support-bound stabilized oligonucleotides with metal salts or organic reagents (RP-02)

DMT-cleavage: DMT-protecting group of DNA strand bound to 1000 Å controlled pore glass (CPG) solid support (1 µmol, ~40 mg of 10mer T7De8a-dGC- and 10mer 7De-dAT7De8a-dGC-sequence) was cleaved by addition of 200 µL 3% trichloroacetic acid in CH_2Cl_2 for 1 min. Orange coloring of the solution indicated successful removal of protecting group. The deprotection was repeated 3-5 times until no further coloring of the solution was observed. CPG-bound deprotected DNA was washed three times with each 200 µL of 1% TEA in ACN, DMF, MeOH, ACN and CH_2Cl_2 and dried *in vacuo*.

Investigation of stability: 20 nmol of CPG-bound stabilized oligonucleotide (0.75 mg) were treated with 200 equiv. of metal salt/organic reagent (4 µmol) solved in 50 µL dry solvent. The suspension was shaken at ambient temperature for 22 h. Afterwards the solvent was removed under vacuum filtration, CPG was washed three times with each 200 µL of 0.1 M

EDTA solution, 0.1 M $MgCl_2$ solution, water, DMF, MeOH, ACN and CH_2Cl_2 and dried *in vacuo*.

Cleavage and Analysis: DNA was deprotected and cleaved from CPG by shaking in 500 µL of an AMA solution (AMA = aqueous ammonia (30%)/ aqueous methylamine (40%), 1:1, vol/vol) for 4 h at room temperature. Afterwards 20 µL of 1 M Tris buffer (pH = 7.5) were added, the mixture was dried under reduced pressure (SpeedVac) and DNA was dissolved in 200 µL distilled water. The product was analyzed by analytical RP-HPLC and MALDI-TOF-MS.

Table S4 – Stability of chemically modified oligonucleotides **5** and **6** in the presence of different metal salts, organocatalysts and acids [a]

Entry	Reagent	Solvent	T7De8a-dGC **5**	7De-dAT7De8a-dGC **6**
1	3.7% HCl	H_2O	(orange)	(orange)
2	$Ce(NH_4)_2(NO_3)_6$	MeOH	(orange)	(light green)
3	$Co(acac)_3$	ACN	(green)	(light green)
4	$Cu(MeCN)_4PF_6$	ACN	(green)	(green)
5	$FeCl_2 \cdot 4\,H_2O$	ACN	(green)	(green)
6[b]	La(O*i*-Pr)$_3$	THF	(green)	(green)
7	$Ni(acac)_2$	ACN	(green)	(green)
8	$Pd(OAc)_2$	ACN	(red)	(red)
9	$RuCl_3$	ACN	(light green)	(light green)
10	Grubbs 1st Gen.	CH_2Cl_2	(green)	(light green)
11	$Sc(OTf)_3$	ACN	(green)	(light green)
12[c]	$Sc(OTf)_3$	ACN	(light green)	(orange)
13	SeO_2	MeOH	(green)	(green)
14	$VO(acac)_2$	MeOH	(green)	(green)
15	$ZnCl_2$	ACN	(green)	(green)
16	DDQ **7**	EtOH	(green)	(green)
17	PIDA **8**	ACN	(green)	(light green)
18	TEMPO **9**	ACN	(green)	(green)

[a] For each: 20 nmol DNA, aqueous acids or 200 equiv. transition metal salt or 200 equiv. organic reagent, 50 µL solvent, rt, 22 h. [b] Poor solubility, added as suspension. [c] Experiment was performed at 40 °C. ACN = acetonitrile, MeOH = methanol.

5 = 5'-TT7De8a-dG CT7De8a-dG CC7De8a-dG T-3'-CPG

6 = 5'-7De8a-dGTC 7De-dAT7De8a-dG 7De-dATC T-3'-CPG

(green)	(light green)	(orange)	(red)	degree of
0-20%	21-40%	41-60%	> 61%	DNA degradation

HPLC traces and MALDI-MS spectra of metal ion screens

CPG-oligonucleotide + 3.7% HCl

According to the representative procedure RP-01 solid support-coupled oligonucleotide (20 nmol) was treated with 3.7% HCl.

<table>
<tr><th>CPG-oligonucleotide</th><th>Analytical data</th></tr>
<tr><td>10mer T7De8a-dGC

Analytical RP-HPLC (I) trace</td><td>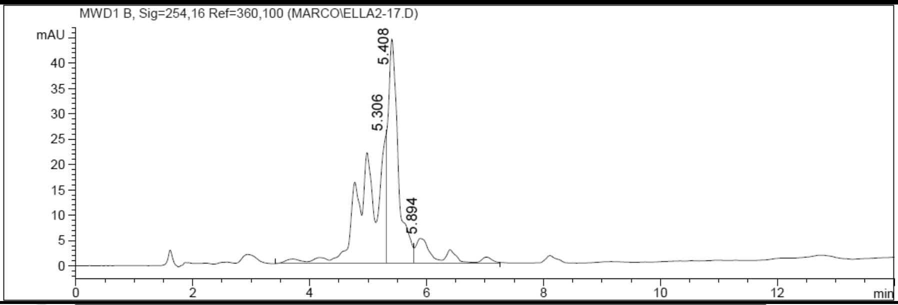
</td></tr>
<tr><td>MALDI-MS spectrum</td><td>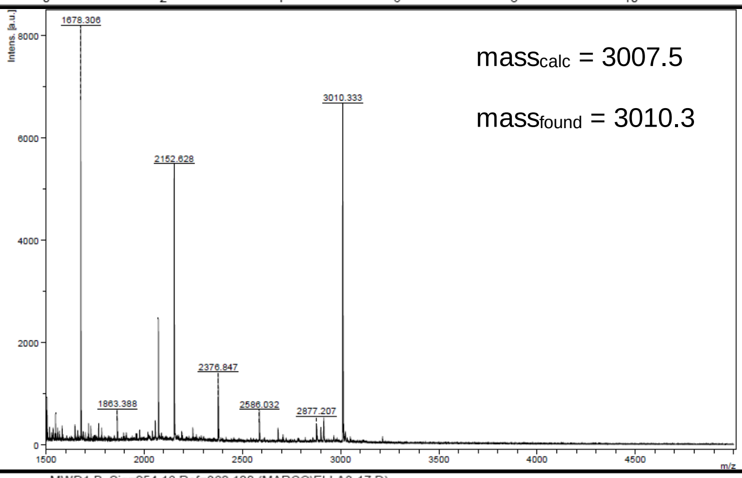

$mass_{calc}$ = 3007.5
$mass_{found}$ = 3010.3</td></tr>
<tr><td>10mer 7De-dAT7De8a-dGC

Analytical RP-HPLC (I) trace</td><td>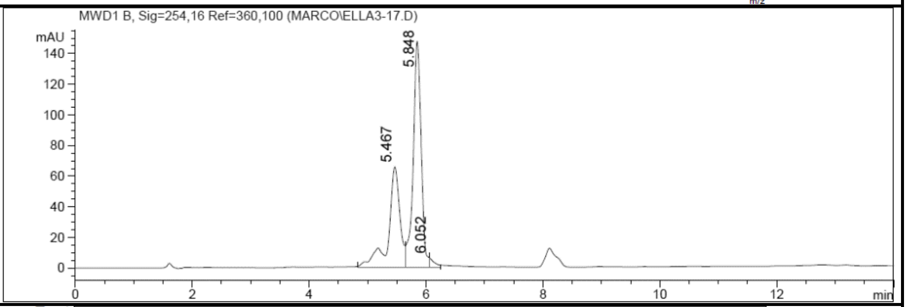
</td></tr>
<tr><td>MALDI-MS spectrum</td><td>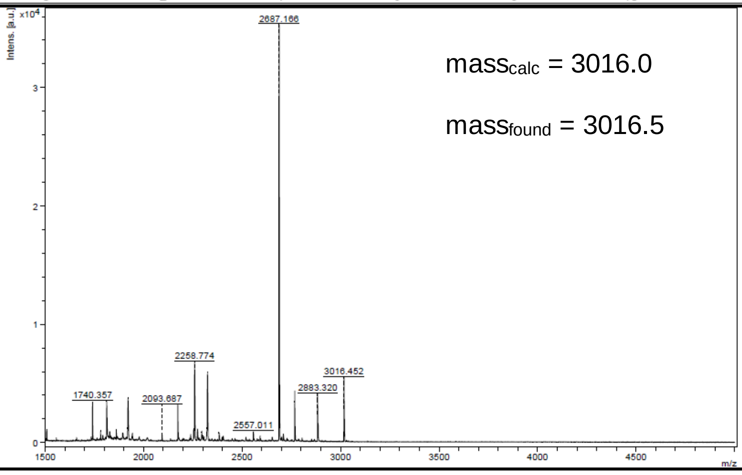

$mass_{calc}$ = 3016.0
$mass_{found}$ = 3016.5</td></tr>
</table>

CPG-oligonucleotide + $Ce(NH_4)_2(NO_3)_6$

According to the representative procedure RP-02 solid support-coupled oligonucleotide (20 nmol) was treated with $Ce(NH_4)_2(NO_3)_6$.

CPG-oligonucleotide	Analytical data
10mer T7De8a-dGC<br><br>Analytical RP-HPLC (I) trace	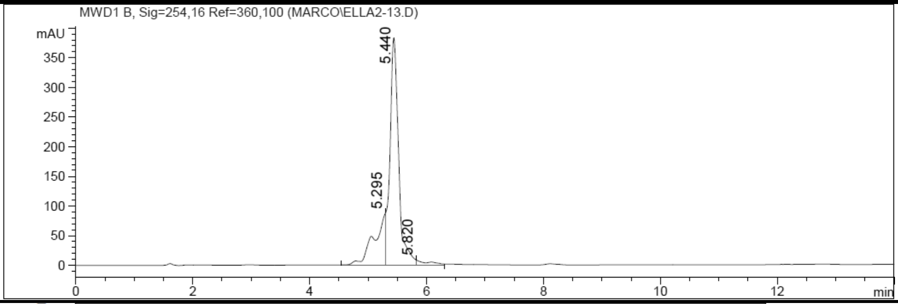
MALDI-MS spectrum	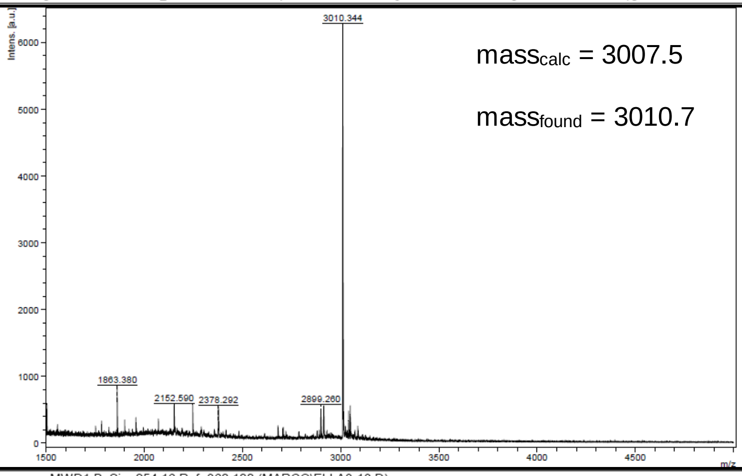
10mer 7De-dAT7De8a-dGC<br><br>Analytical RP-HPLC (I) trace	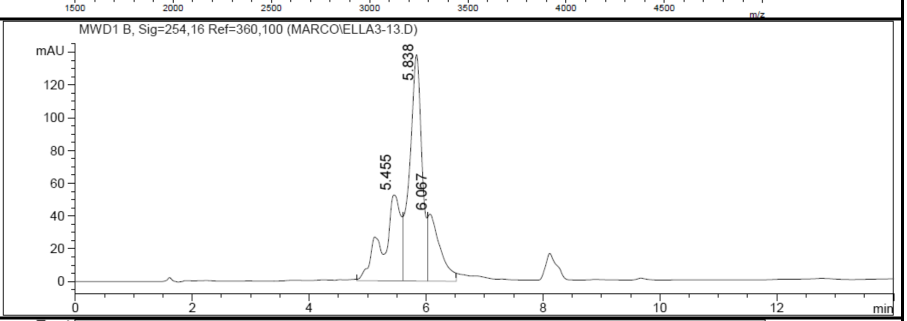
MALDI-MS spectrum	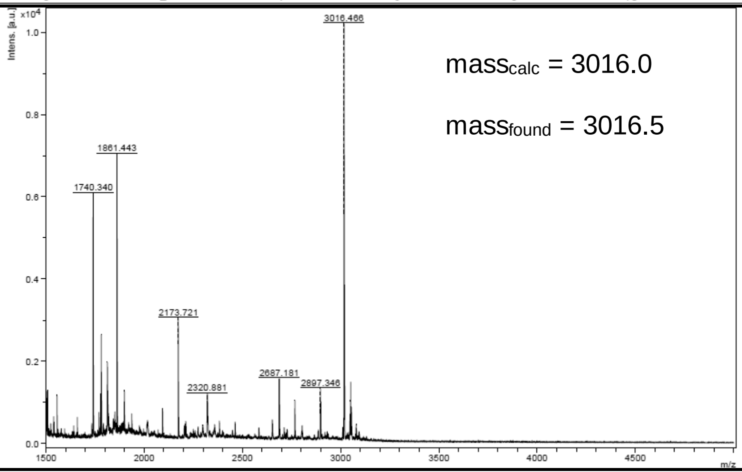

CPG-oligonucleotide + Co(acac)$_3$

According to the representative procedure RP-02 solid support-coupled oligonucleotide (20 nmol) was treated with Co(acac)$_3$.

CPG-oligonucleotide	Analytical data
10mer T7De8a-dGC<br><br>Analytical RP-HPLC (I) trace	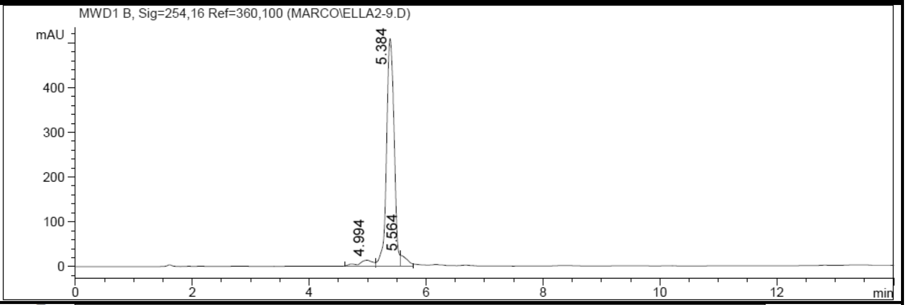
MALDI-MS spectrum	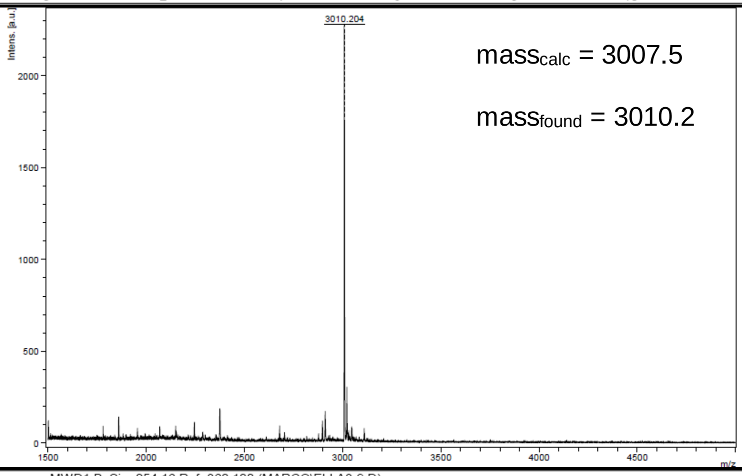<br>mass$_{calc}$ = 3007.5<br>mass$_{found}$ = 3010.2
10mer 7De-dAT7De8a-dGC<br><br>Analytical RP-HPLC (I) trace	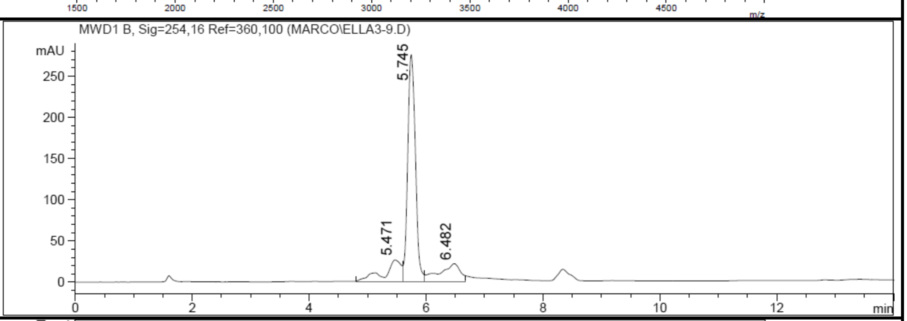
MALDI-MS spectrum	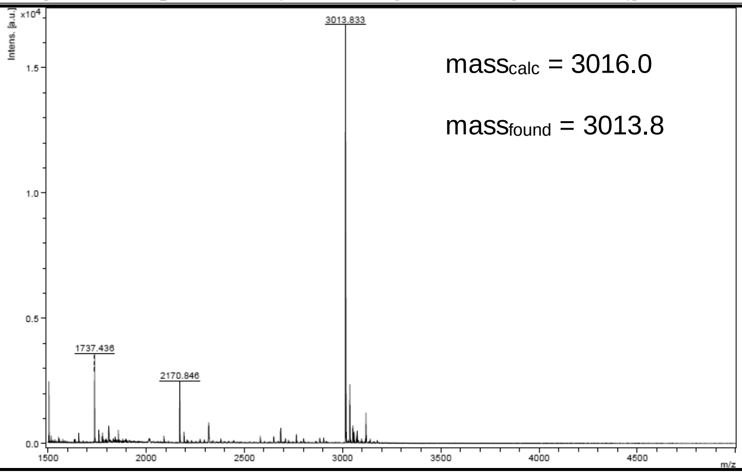<br>mass$_{calc}$ = 3016.0<br>mass$_{found}$ = 3013.8

CPG-oligonucleotide + $Cu(CH_3CN)_4PF_6$

According to the representative procedure RP-02 solid support-coupled oligonucleotide (20 nmol) was treated with $Cu(CH_3CN)_4PF_6$.

CPG-oligonucleotide	Analytical data
10mer T7De8a-dGC<br><br>Analytical RP-HPLC (I) trace	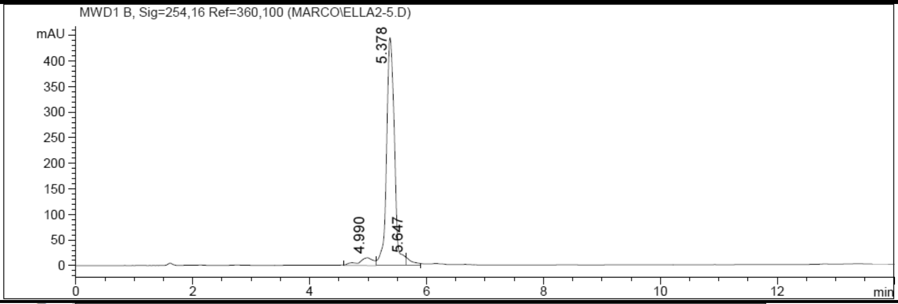
MALDI-MS spectrum	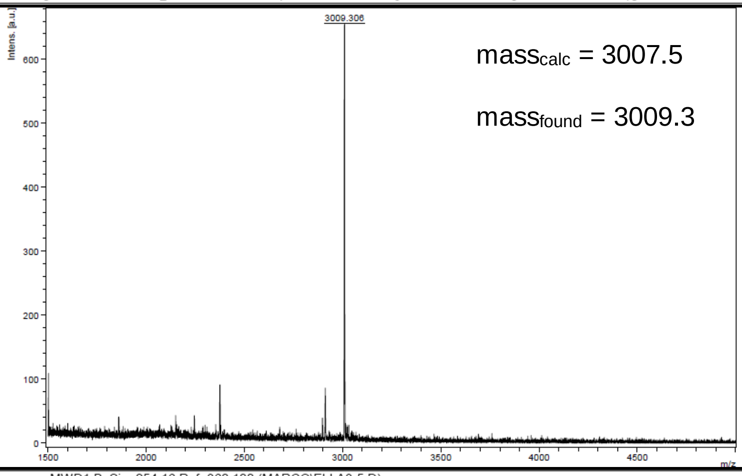<br>$mass_{calc}$ = 3007.5<br>$mass_{found}$ = 3009.3
10mer 7De-dAT7De8a-dGC<br><br>Analytical RP-HPLC (I) trace	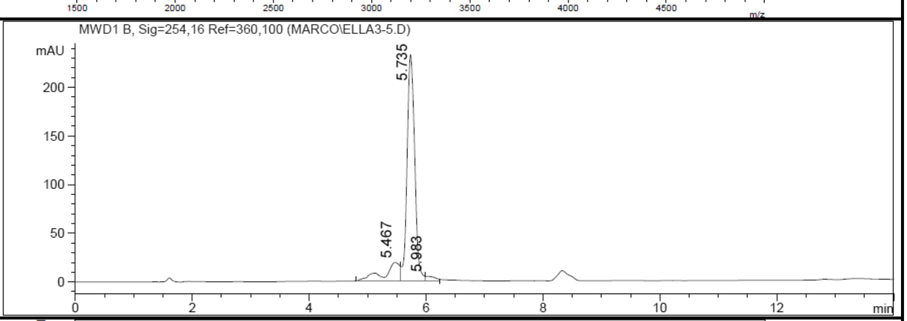
MALDI-MS spectrum	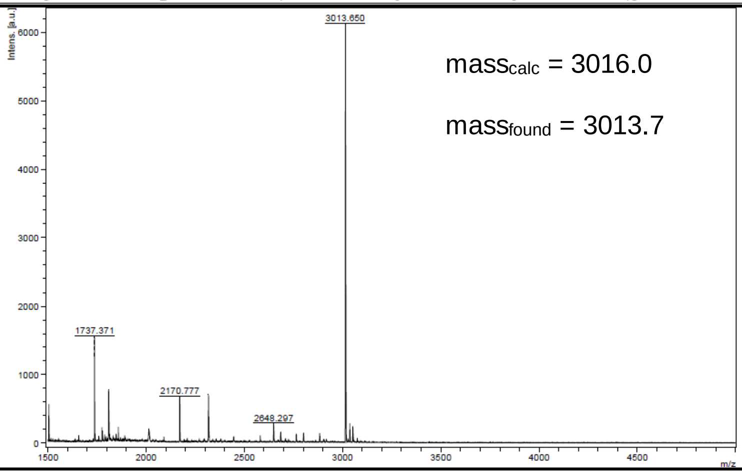<br>$mass_{calc}$ = 3016.0<br>$mass_{found}$ = 3013.7

CPG-oligonucleotide + $FeCl_2 \cdot 4\ H_2O$

According to the representative procedure RP-02 solid support-coupled oligonucleotide (20 nmol) was treated with $FeCl_2 \cdot 4\ H_2O$.

CPG-oligonucleotide	Analytical data
10mer T7De8a-dGC<br><br>Analytical RP-HPLC (I) trace	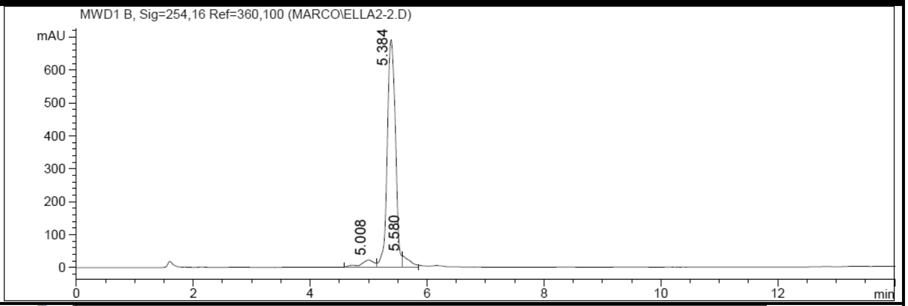
MALDI-MS spectrum	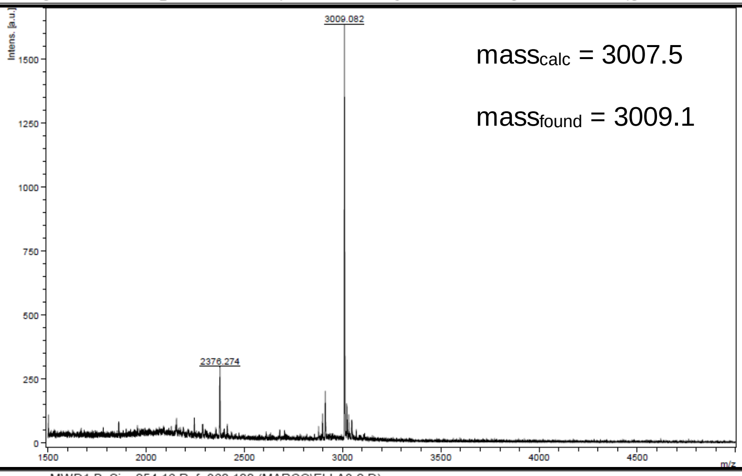<br>$mass_{calc}$ = 3007.5<br>$mass_{found}$ = 3009.1
10mer 7De-dAT7De8a-dGC<br><br>Analytical RP-HPLC (I) trace	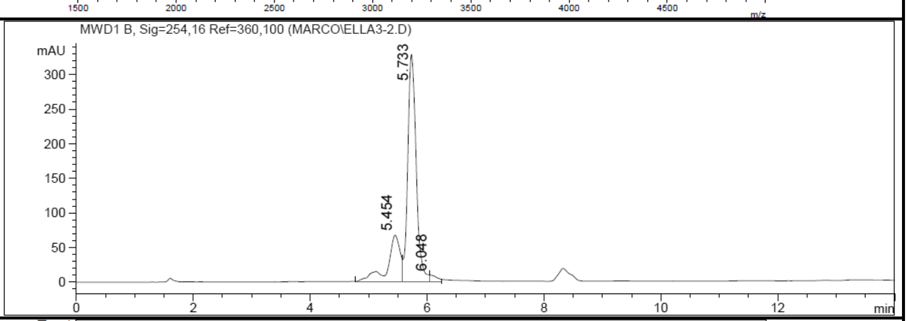
MALDI-MS spectrum	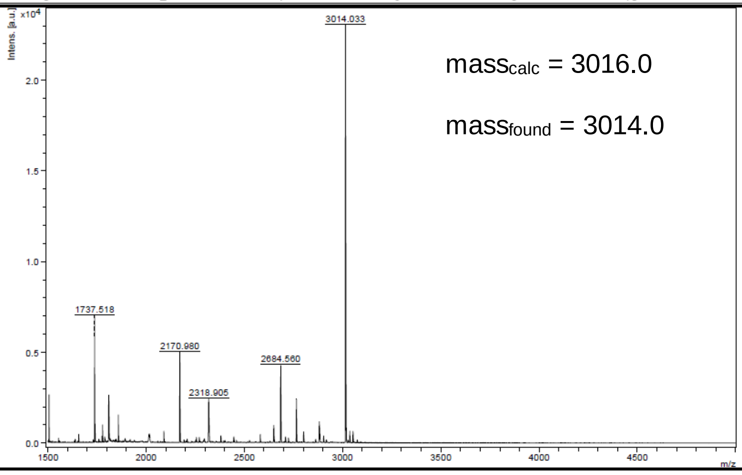<br>$mass_{calc}$ = 3016.0<br>$mass_{found}$ = 3014.0

CPG-oligonucleotide + La(O*i*-Pr)$_3$

According to the representative procedure RP-02 solid support-coupled oligonucleotide (20 nmol) was treated with La(O*i*-Pr)$_3$.

CPG-oligonucleotide	Analytical data
10mer T7De8a-dGC<br><br>Analytical RP-HPLC (I) trace	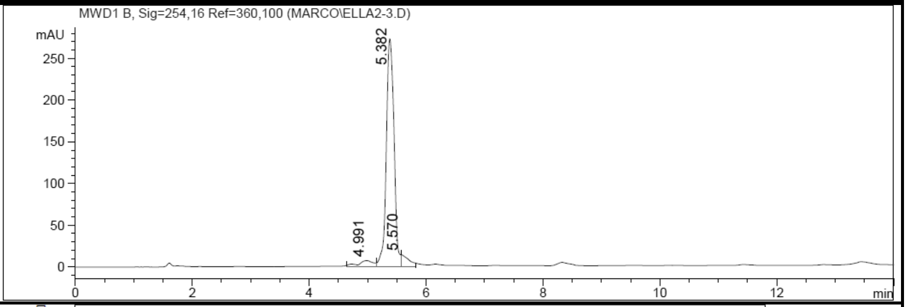
MALDI-MS spectrum	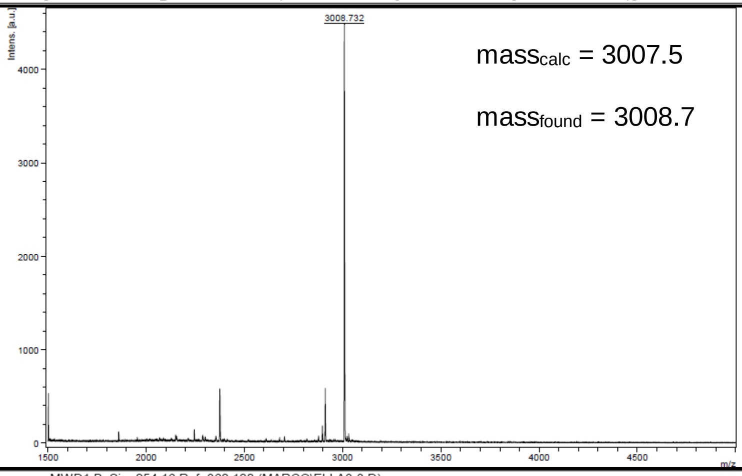<br>mass$_{calc}$ = 3007.5<br>mass$_{found}$ = 3008.7
10mer 7De-dAT7De8a-dGC<br><br>Analytical RP-HPLC (I) trace	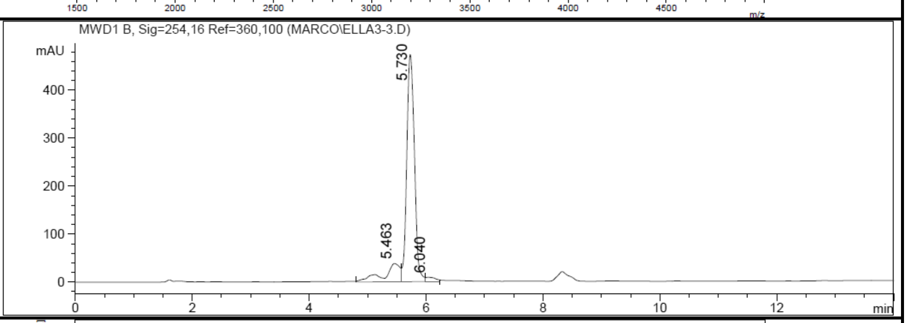
MALDI-MS spectrum	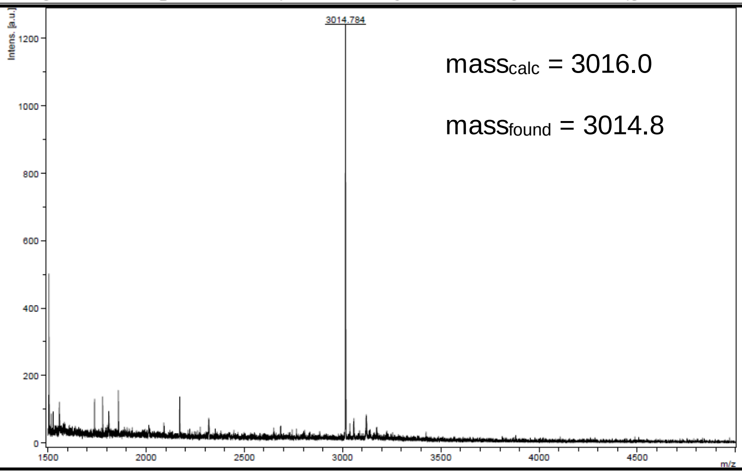<br>mass$_{calc}$ = 3016.0<br>mass$_{found}$ = 3014.8

CPG-oligonucleotide + $Ni(acac)_2$

According to the representative procedure RP-02 solid support-coupled oligonucleotide (20 nmol) was treated with $Ni(acac)_2$.

<table>
<tr><th>CPG-oligonucleotide</th><th>Analytical data</th></tr>
<tr><td>10mer T7De8a-dGC

Analytical RP-HPLC (I) trace</td><td>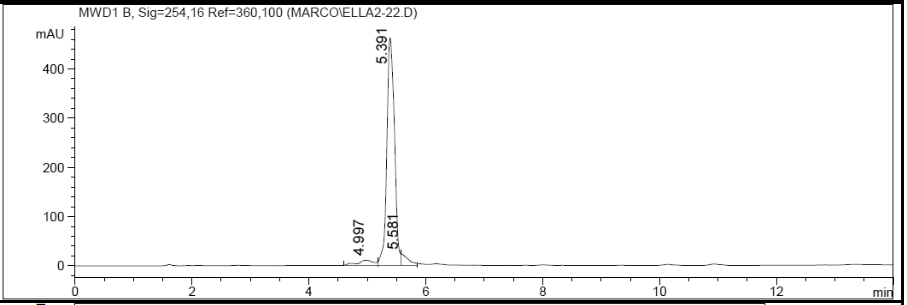
</td></tr>
<tr><td>MALDI-MS spectrum</td><td>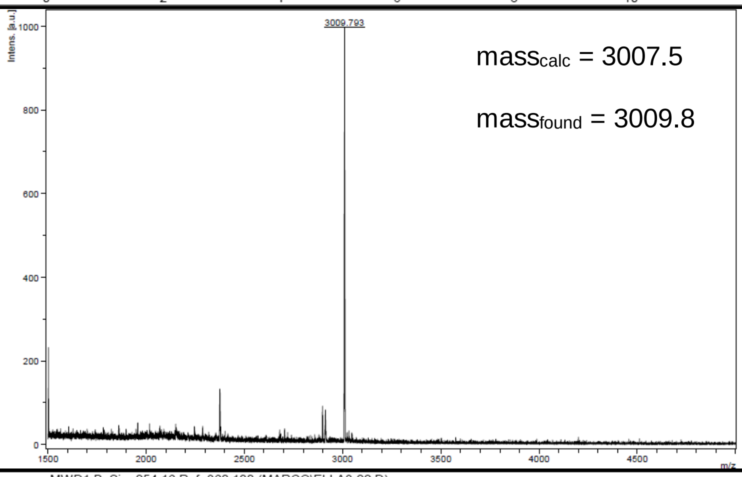
</td></tr>
<tr><td>10mer 7De-dAT7De8a-dGC

Analytical RP-HPLC (I) trace</td><td>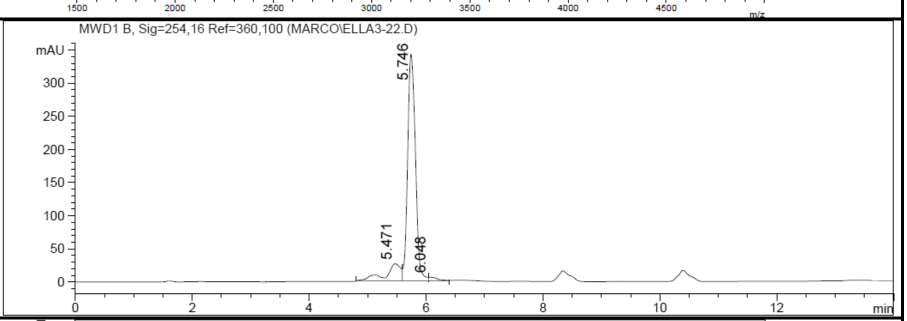
</td></tr>
<tr><td>MALDI-MS spectrum</td><td>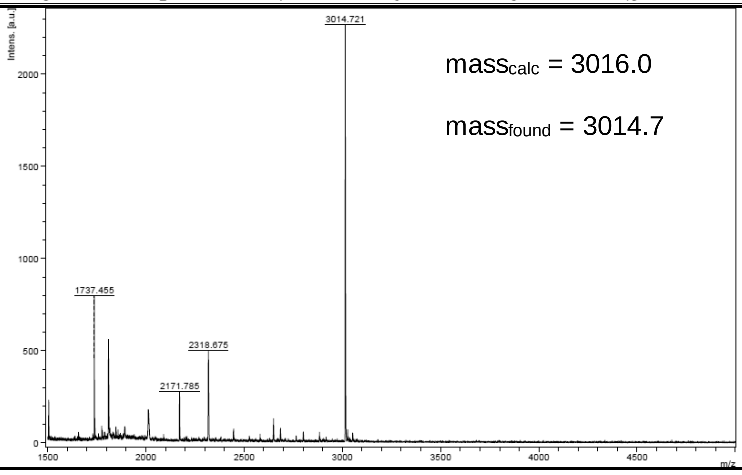
</td></tr>
</table>

CPG-oligonucleotide + $PdOAc_2$

According to the representative procedure RP-02 solid support-coupled oligonucleotide (20 nmol) was treated with $PdOAc_2$.

<table>
<tr><th>CPG-oligonucleotide</th><th>Analytical data</th></tr>
<tr><td>10mer T7De8a-dGC

Analytical RP-HPLC (I) trace</td><td>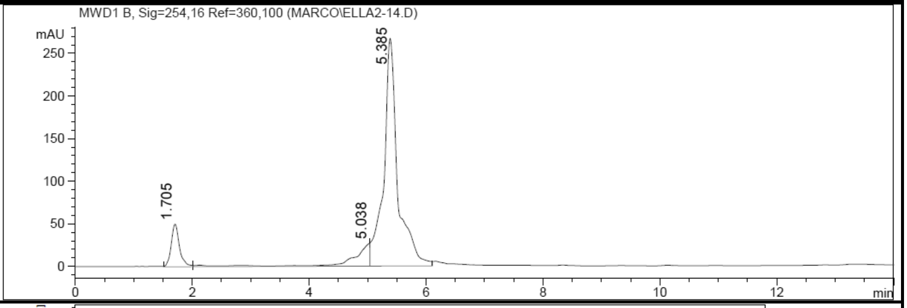
</td></tr>
<tr><td>MALDI-MS spectrum</td><td>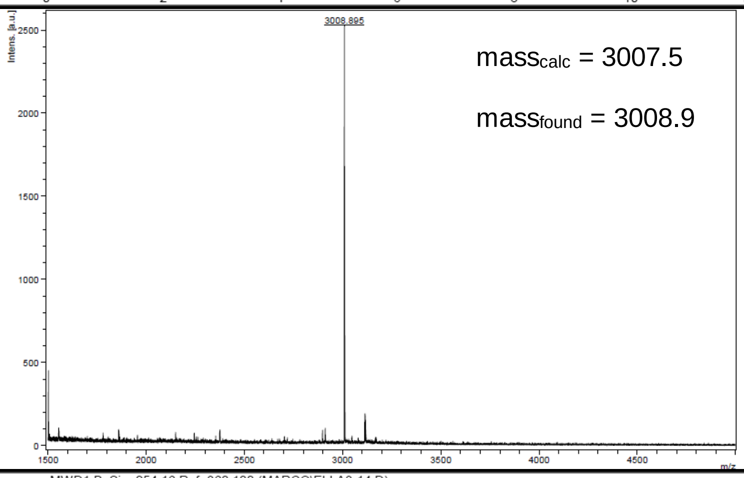
</td></tr>
<tr><td>10mer 7De-dAT7De8a-dGC

Analytical RP-HPLC (I) trace</td><td>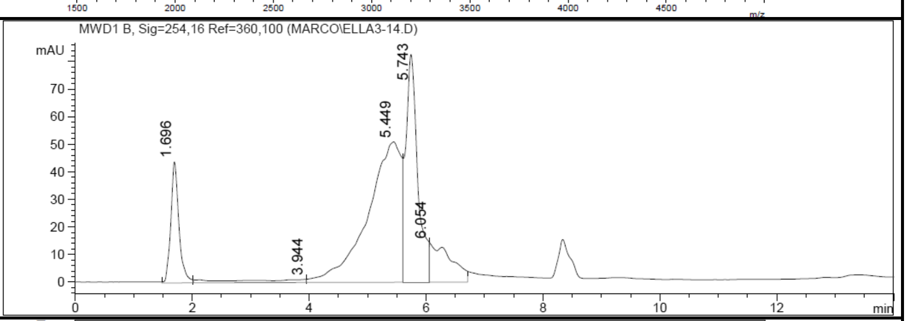
</td></tr>
<tr><td>MALDI-MS spectrum</td><td>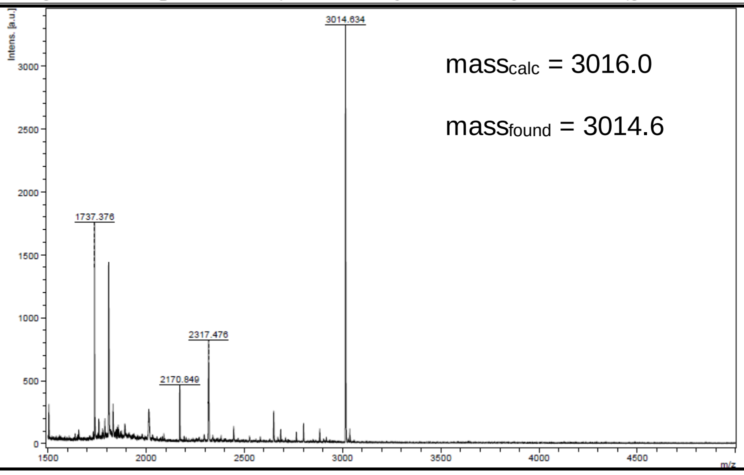
</td></tr>
</table>

CPG-oligonucleotide + $RuCl_3$

According to the representative procedure RP-02 solid support-coupled oligonucleotide (20 nmol) was treated with $RuCl_3$.

CPG-oligonucleotide	Analytical data
10mer T7De8a-dGC<br><br>Analytical RP-HPLC (I) trace	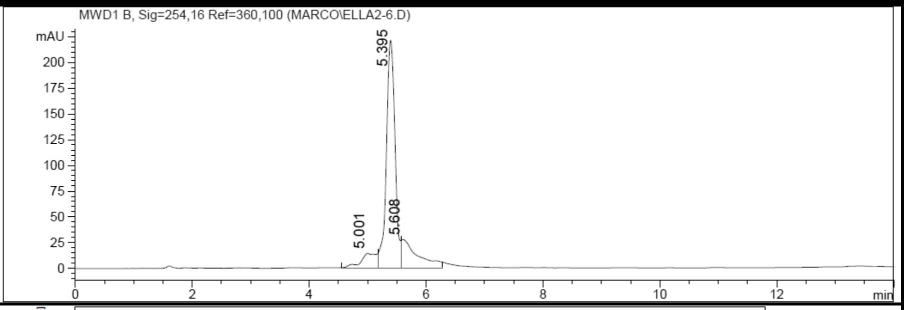
MALDI-MS spectrum	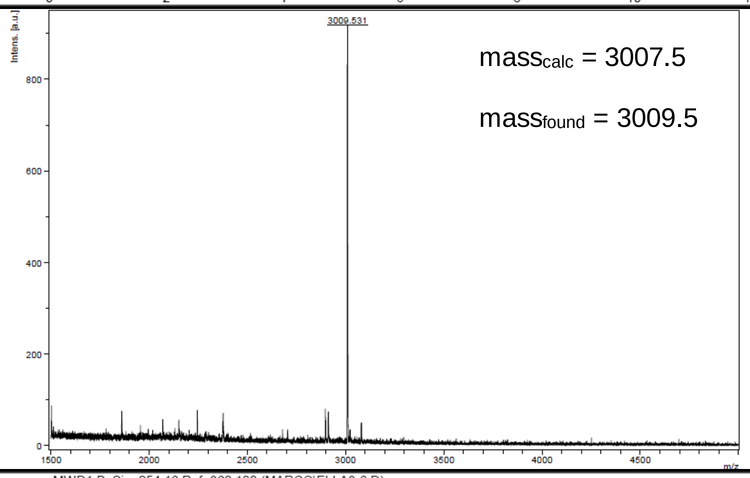
10mer 7De-dAT7De8a-dGC<br><br>Analytical RP-HPLC (I) trace	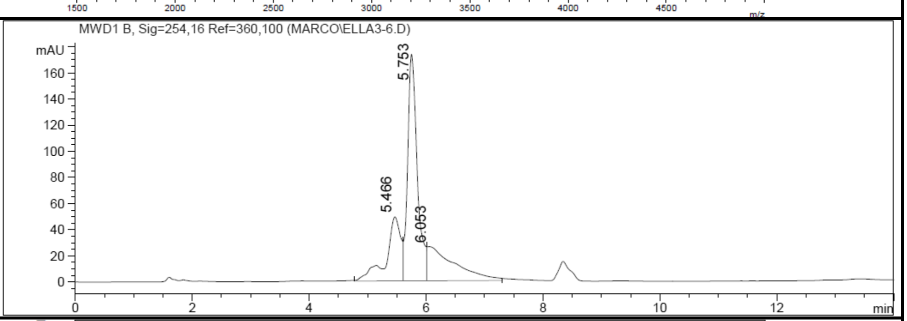
MALDI-MS spectrum	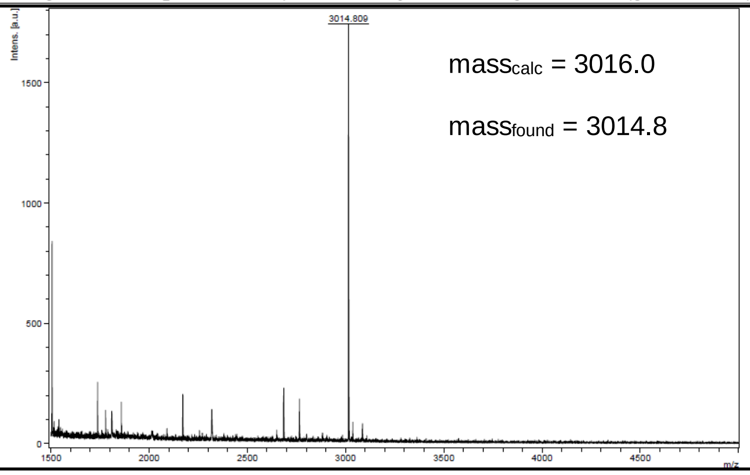

CPG-oligonucleotide + Grubbs 1st Gen.

According to the representative procedure RP-02 solid support-coupled oligonucleotide (20 nmol) was treated with Grubbs 1st Gen..

<table>
<tr><th>CPG-oligonucleotide</th><th>Analytical data</th></tr>
<tr><td>10mer T7De8a-dGC

Analytical RP-HPLC (I) trace</td><td>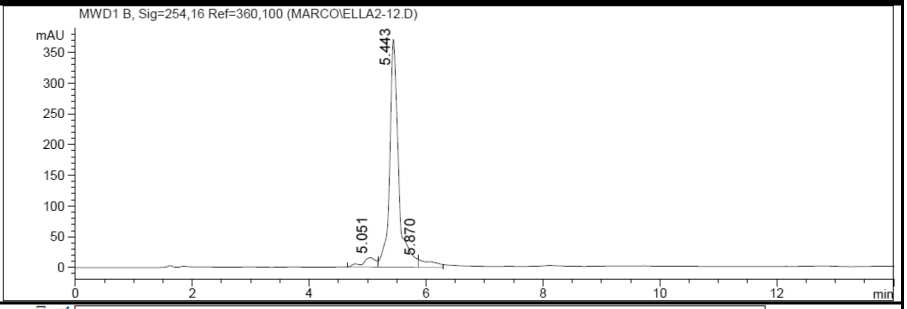
</td></tr>
<tr><td>MALDI-MS spectrum</td><td>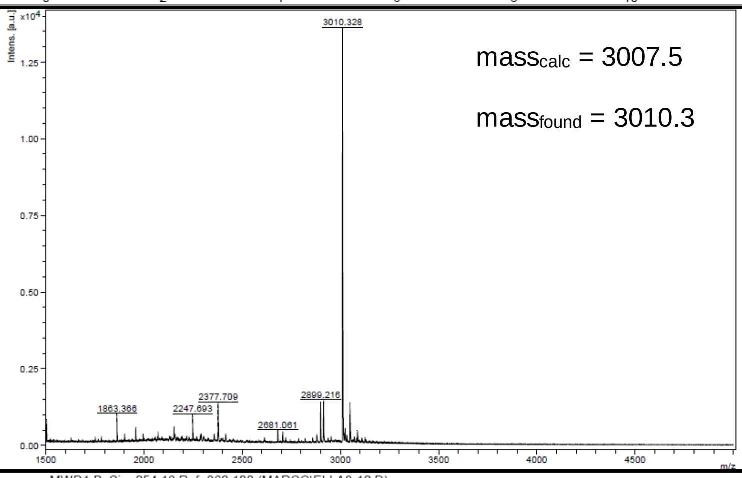
</td></tr>
<tr><td>10mer 7De-dAT7De8a-dGC

Analytical RP-HPLC (I) trace</td><td>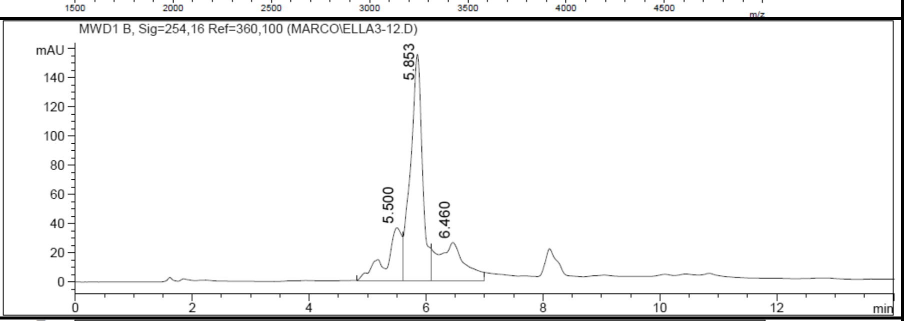
</td></tr>
<tr><td>MALDI-MS spectrum</td><td>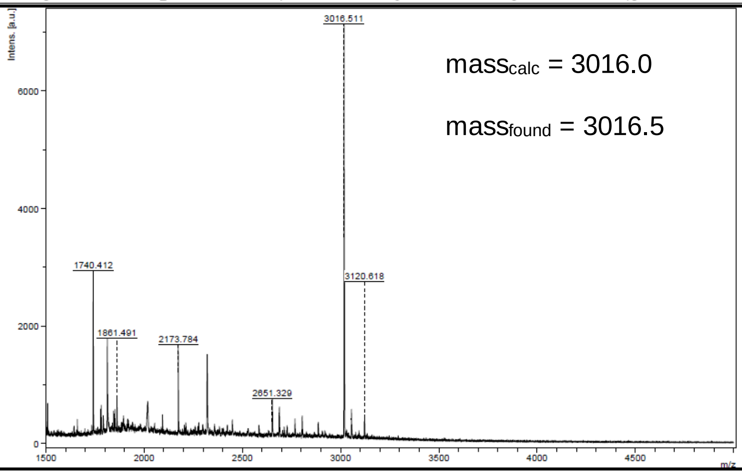
</td></tr>
</table>

CPG-oligonucleotide + Sc(OTf)$_3$

According to the representative procedure RP-02 solid support-coupled oligonucleotide (20 nmol) was treated with Sc(OTf)$_3$.

CPG-oligonucleotide	Analytical data
10mer T7De8a-dGC<br>Analytical RP-HPLC (I) trace	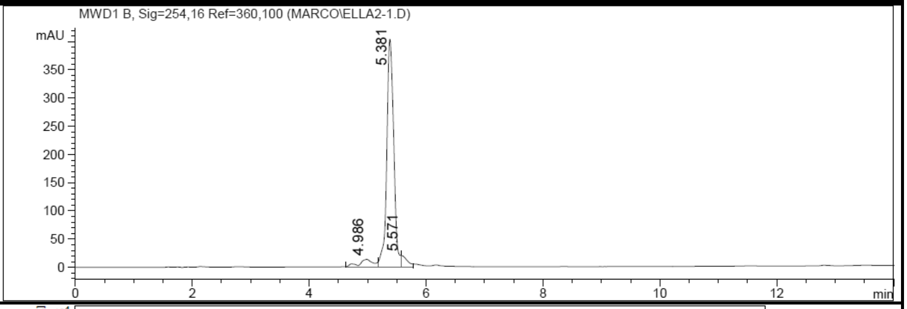
MALDI-MS spectrum	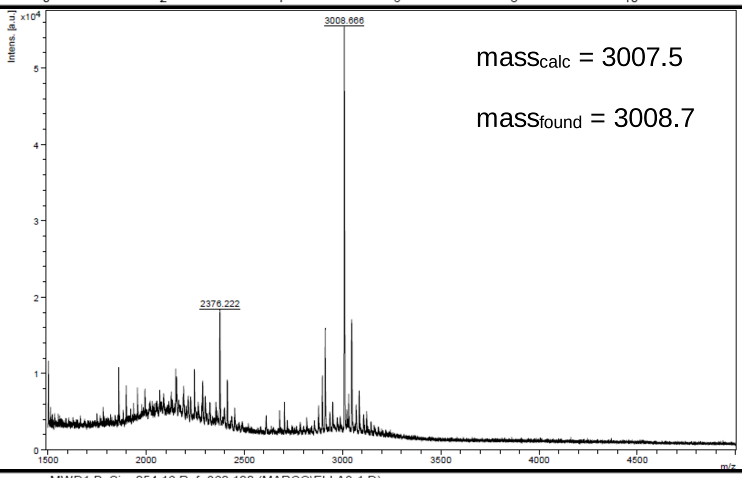
10mer 7De-dAT7De8a-dGC<br>Analytical RP-HPLC (I) trace	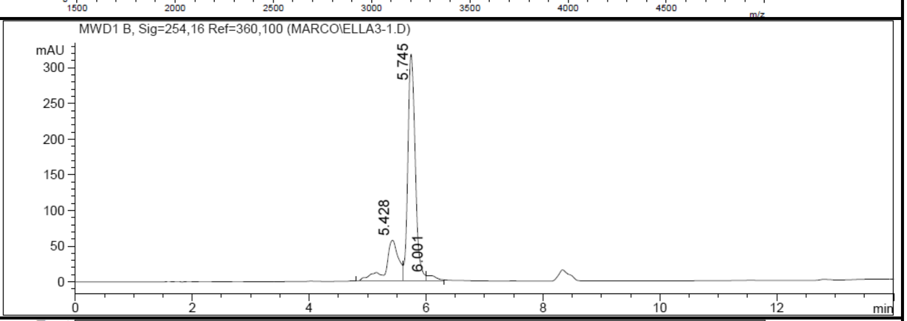
MALDI-MS spectrum	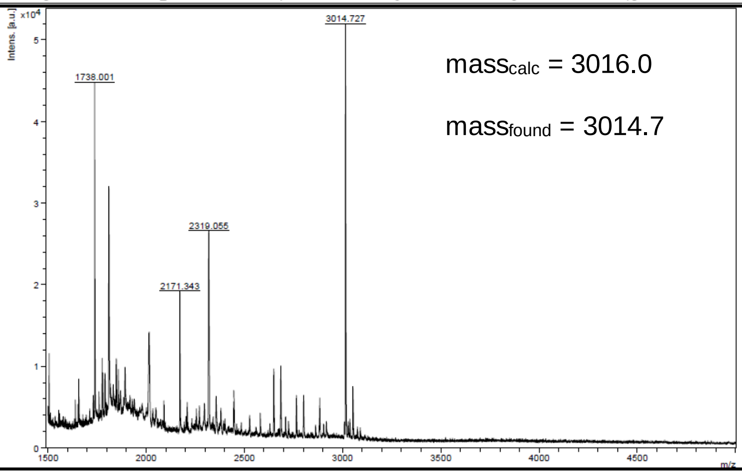

CPG-oligonucleotide + $Sc(OTf)_3$

According to the representative procedure RP-02 solid support-coupled oligonucleotide (20 nmol) was treated with $Sc(OTf)_3$ at 40 °C.

CPG-oligonucleotide	Analytical data
10mer T7De8a-dGC<br><br>Analytical RP-HPLC (I) trace	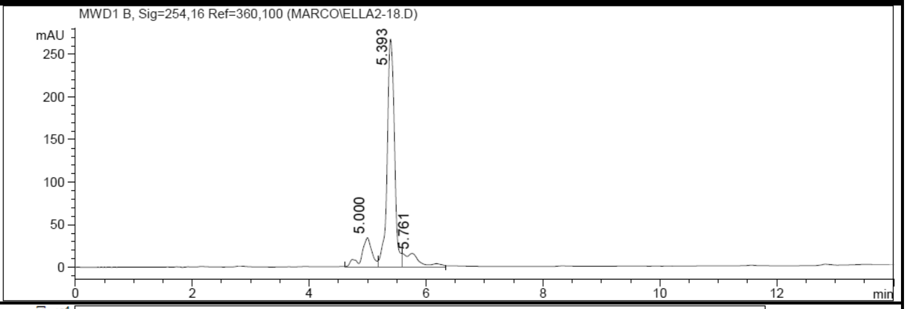
MALDI-MS spectrum	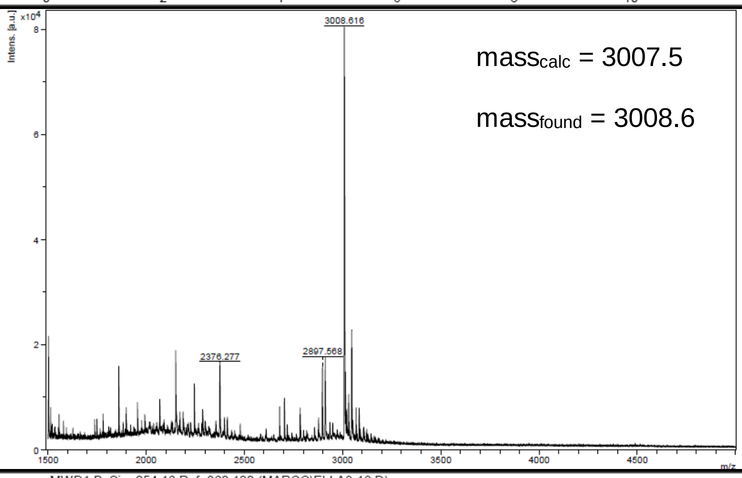
10mer 7De-dAT7De8a-dGC<br><br>Analytical RP-HPLC (I) trace	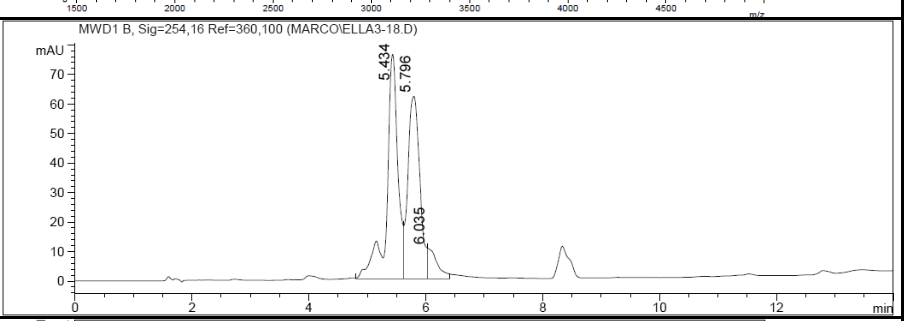
MALDI-MS spectrum	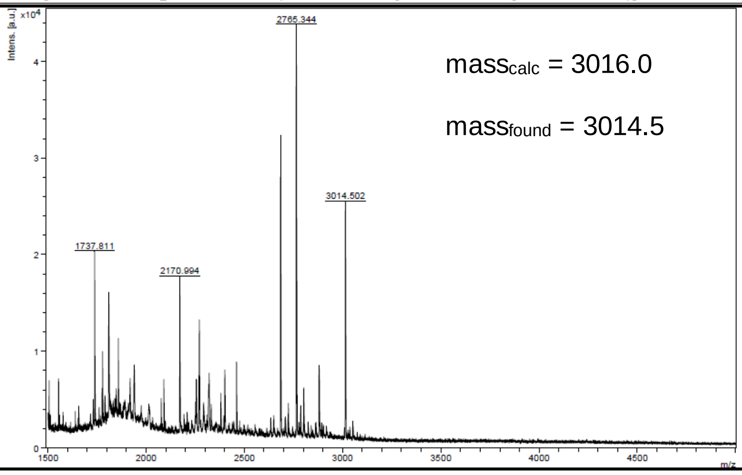

CPG-oligonucleotide + SeO_2

According to the representative procedure RP-02 solid support-coupled oligonucleotide (20 nmol) was treated with SeO_2.

CPG-oligonucleotide	Analytical data
10mer T7De8a-dGC<br><br>Analytical RP-HPLC (I) trace	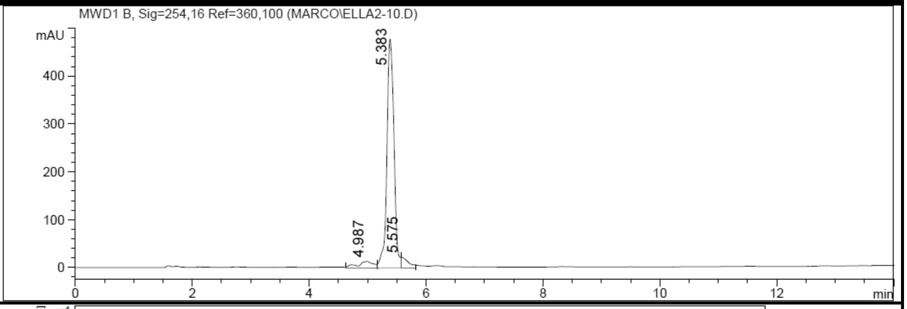
MALDI-MS spectrum	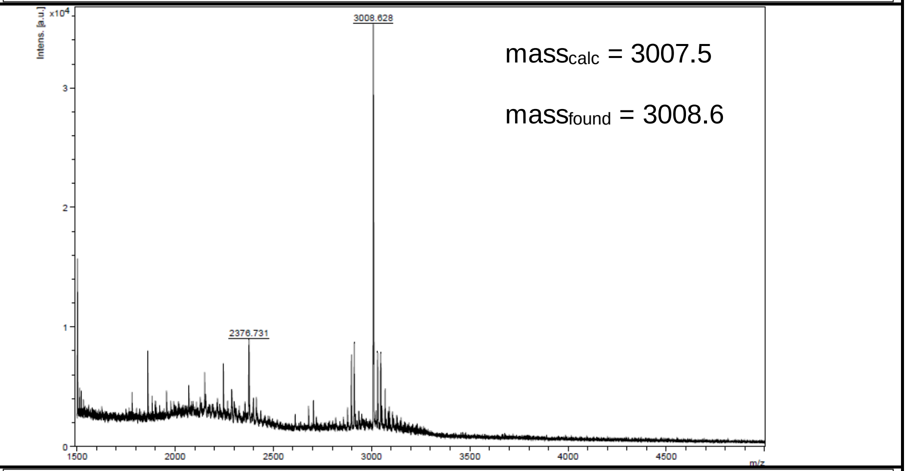
10mer 7De-dAT7De8a-dGC<br><br>Analytical RP-HPLC (I) trace	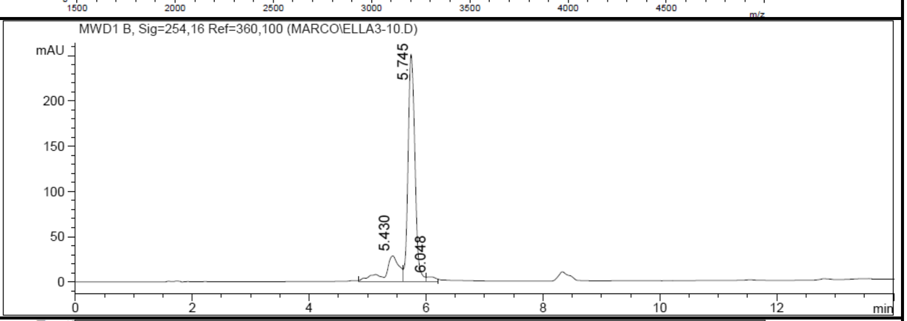
MALDI-MS spectrum	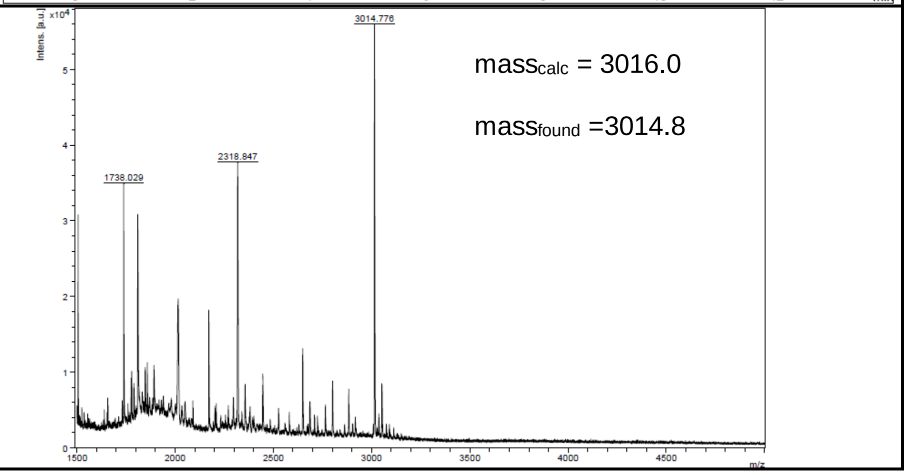

CPG-oligonucleotide + $VO(acac)_2$

According to the representative procedure RP-02 solid support-coupled oligonucleotide (20 nmol) was treated with $VO(acac)_2$.

CPG-oligonucleotide	Analytical data
10mer T7De8a-dGC<br><br>Analytical RP-HPLC (I) trace	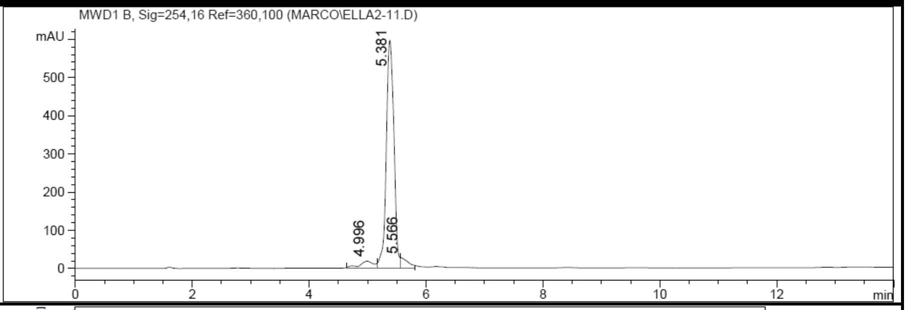
MALDI-MS spectrum	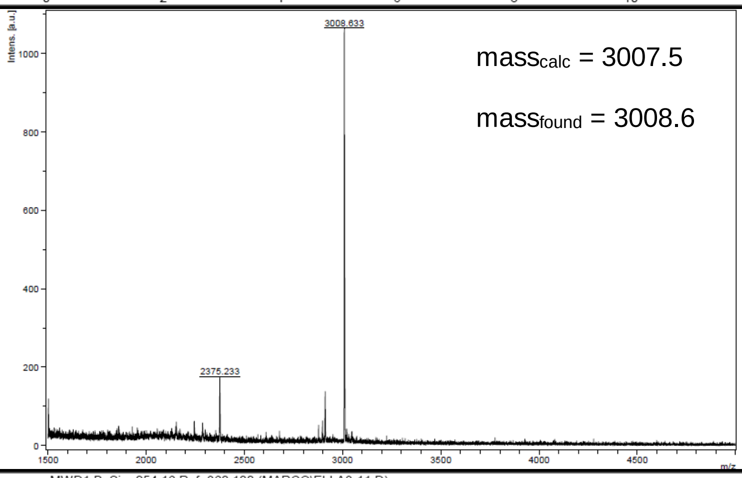
10mer 7De-dAT7De8a-dGC<br><br>Analytical RP-HPLC (I) trace	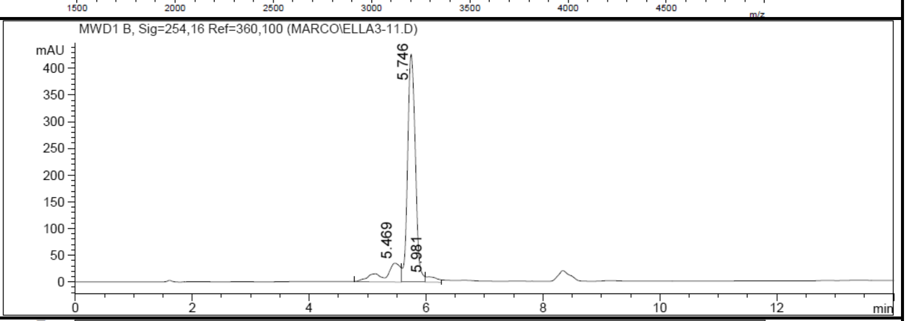
MALDI-MS spectrum	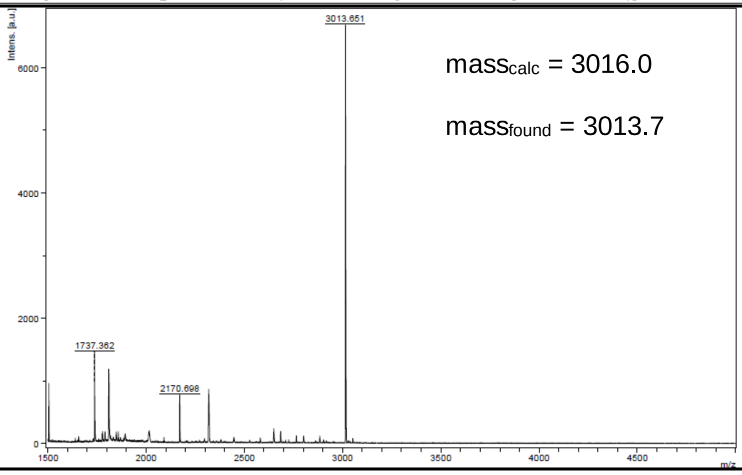

CPG-oligonucleotide + $ZnCl_2$

According to the representative procedure RP-02 solid support-coupled oligonucleotide (20 nmol) was treated with $ZnCl_2$.

<table>
<tr><th>CPG-oligonucleotide</th><th>Analytical data</th></tr>
<tr><td>10mer T7De8a-dGC

Analytical RP-HPLC (I) trace</td><td>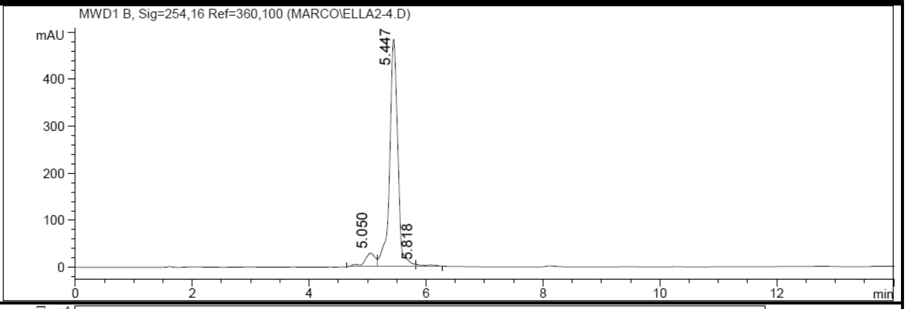
</td></tr>
<tr><td>MALDI-MS spectrum</td><td>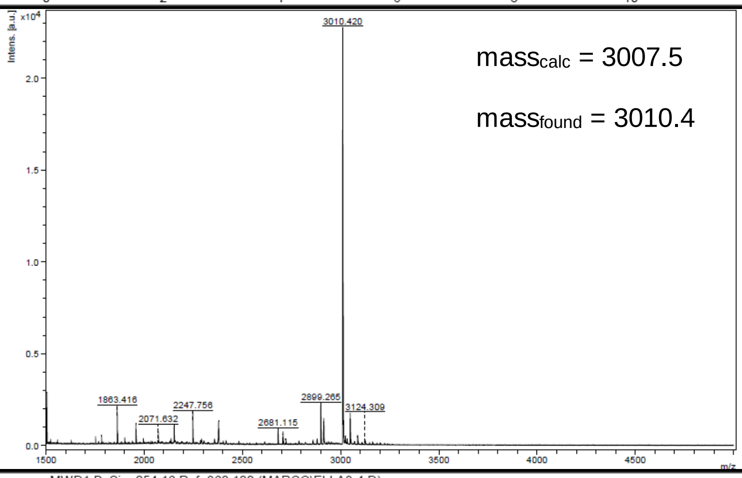
</td></tr>
<tr><td>10mer 7De-dAT7De8a-dGC

Analytical RP-HPLC (I) trace</td><td>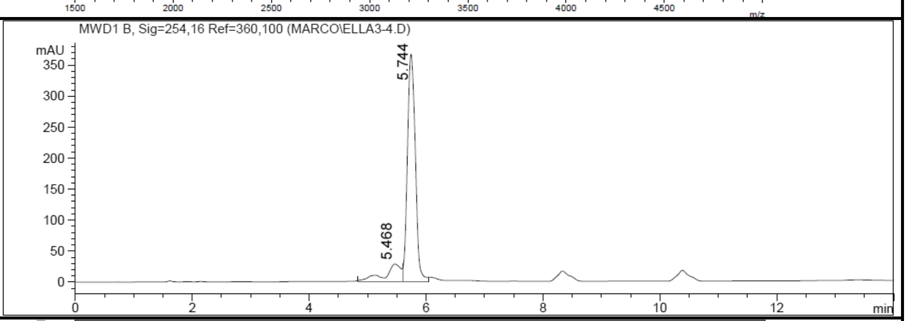
</td></tr>
<tr><td>MALDI-MS spectrum</td><td>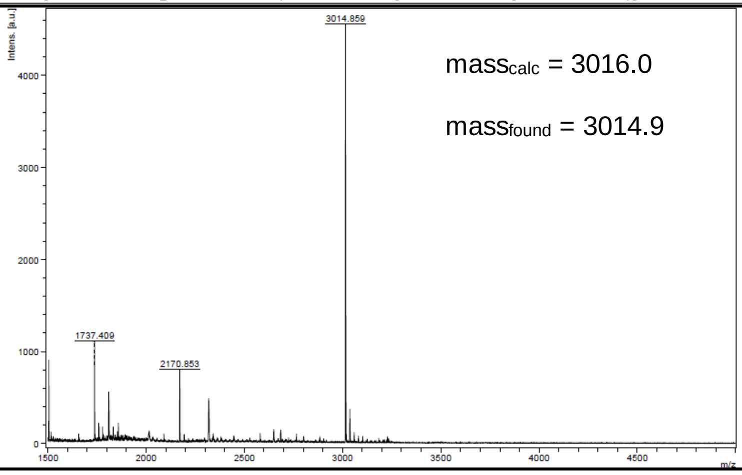
</td></tr>
</table>

CPG-oligonucleotide + DDQ

According to the representative procedure RP-02 solid support-coupled oligonucleotide (20 nmol) was treated with DDQ **7**.

<table>
<tr><th>CPG-oligonucleotide</th><th>Analytical data</th></tr>
<tr><td>10mer T7De8a-dGC

Analytical RP-HPLC (I) trace</td><td>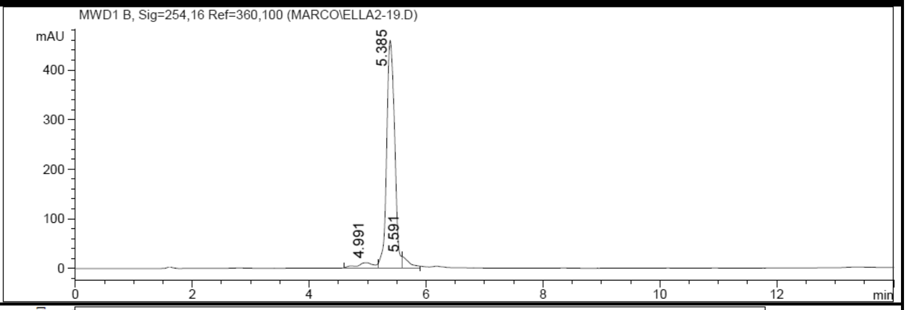
</td></tr>
<tr><td>MALDI-MS spectrum</td><td>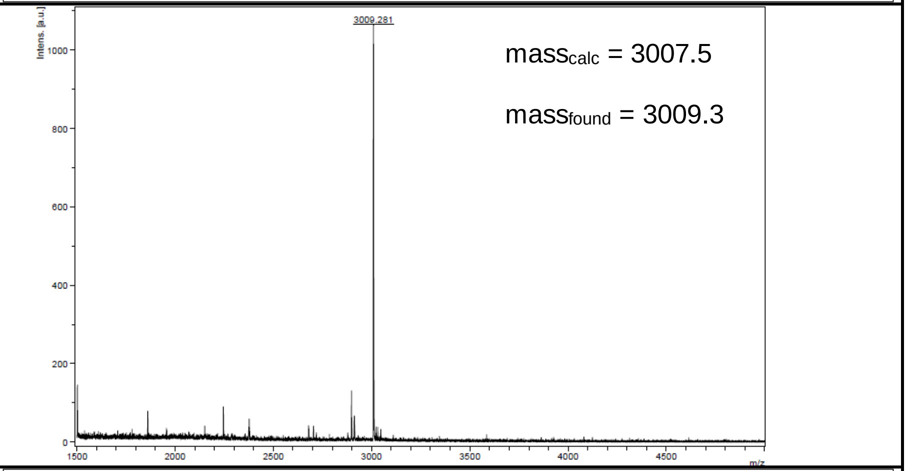
</td></tr>
<tr><td>10mer 7De-dAT7De8a-dGC

Analytical RP-HPLC (I) trace</td><td>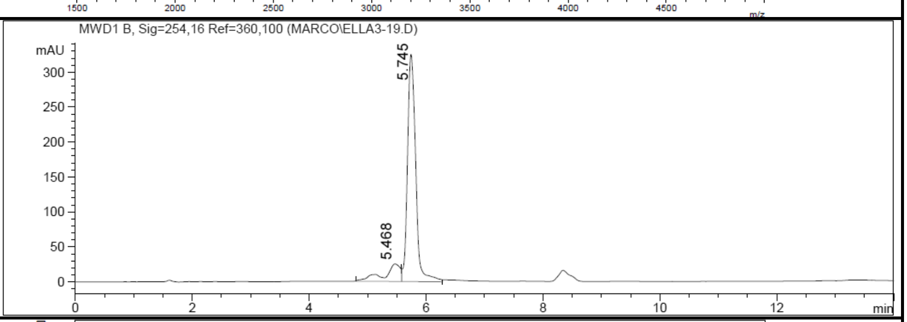
</td></tr>
<tr><td>MALDI-MS spectrum</td><td>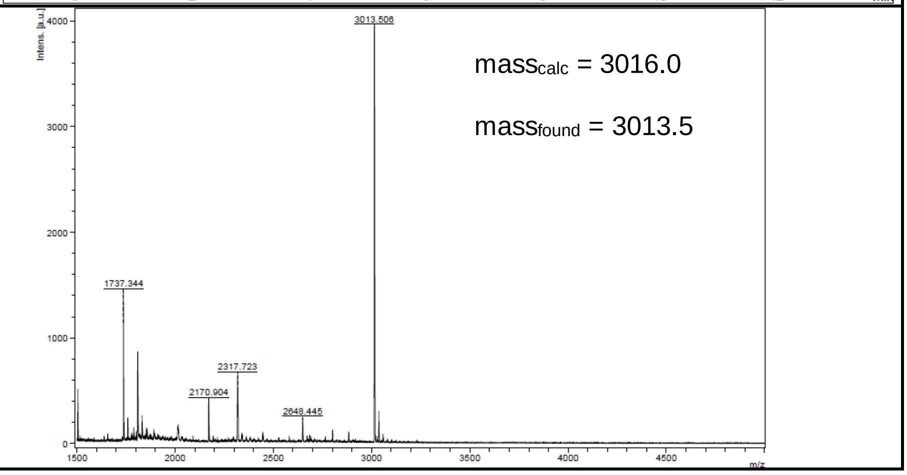
</td></tr>
</table>

CPG-oligonucleotide + PIDA

According to the representative procedure RP-02 solid support-coupled oligonucleotide (20 nmol) was treated with PIDA **8**.

<table>
<tr><th>CPG-oligonucleotide</th><th>Analytical data</th></tr>
<tr><td>10mer T7De8a-dGC

Analytical RP-HPLC (I) trace</td><td>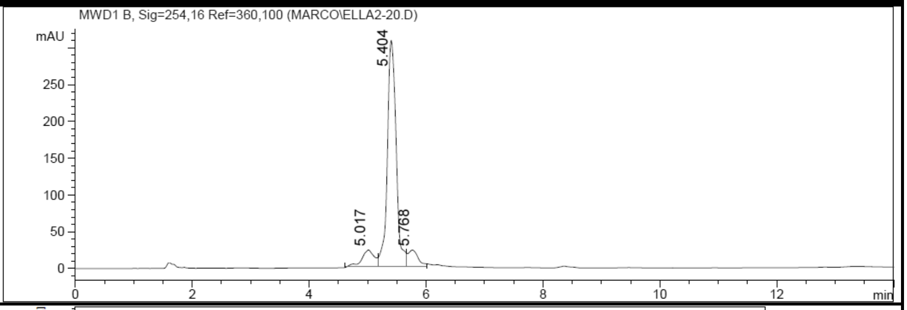
</td></tr>
<tr><td>MALDI-MS spectrum</td><td>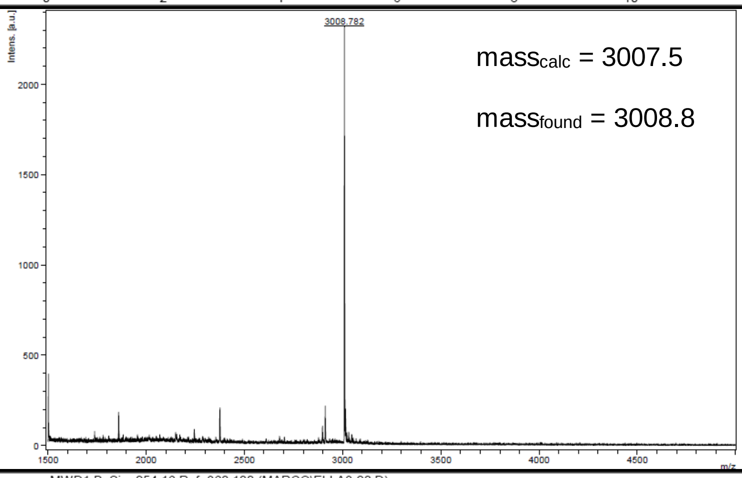
</td></tr>
<tr><td>10mer 7De-dAT7De8a-dGC

Analytical RP-HPLC (I) trace</td><td>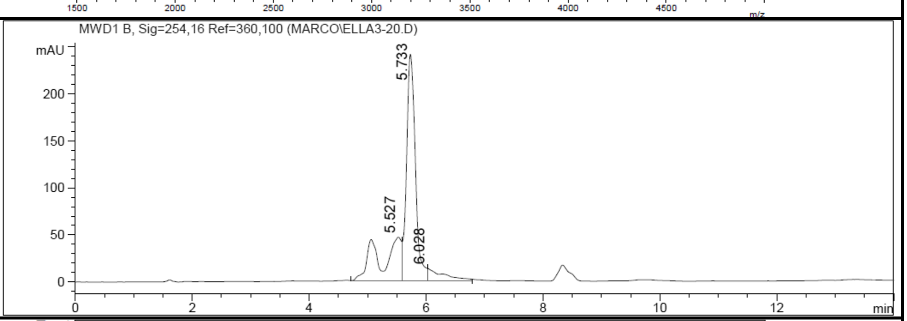
</td></tr>
<tr><td>MALDI-MS spectrum</td><td>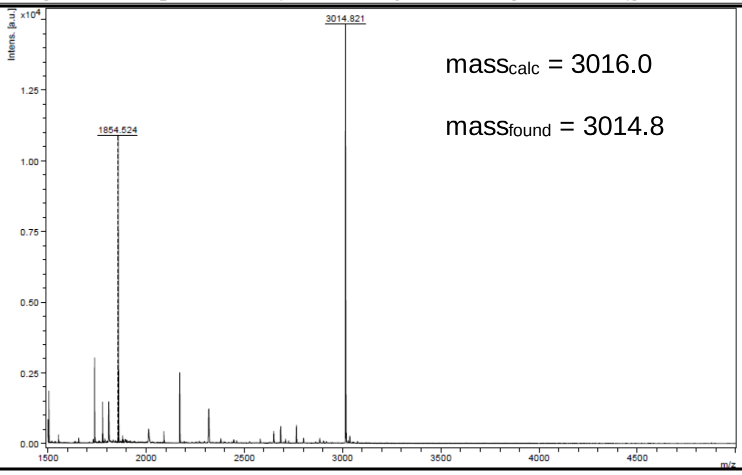
</td></tr>
</table>

CPG-oligonucleotide + TEMPO

According to the representative procedure RP-02 solid support-coupled oligonucleotide (20 nmol) was treated with TEMPO **9**.

CPG-oligonucleotide	Analytical data
10mer T7De8a-dGC<br><br>Analytical RP-HPLC (I) trace	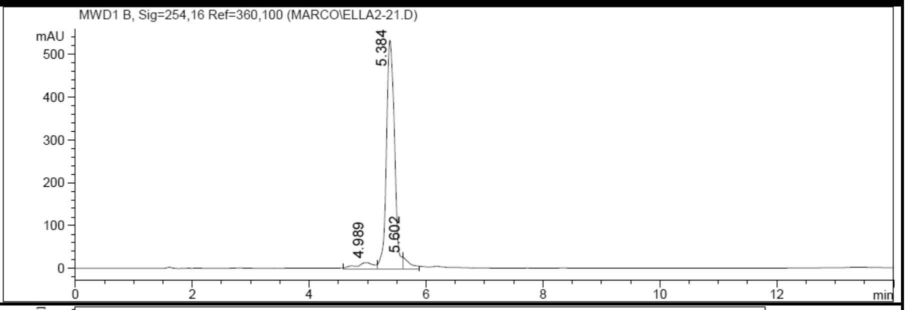
MALDI-MS spectrum	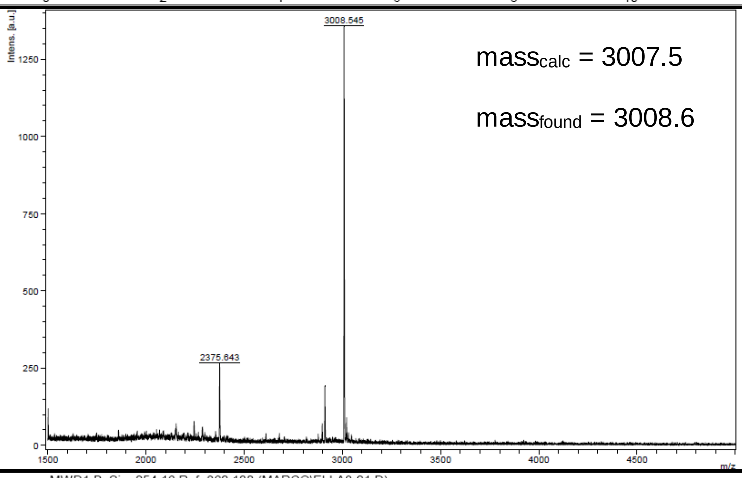
10mer 7De-dAT7De8a-dGC<br><br>Analytical RP-HPLC (I) trace	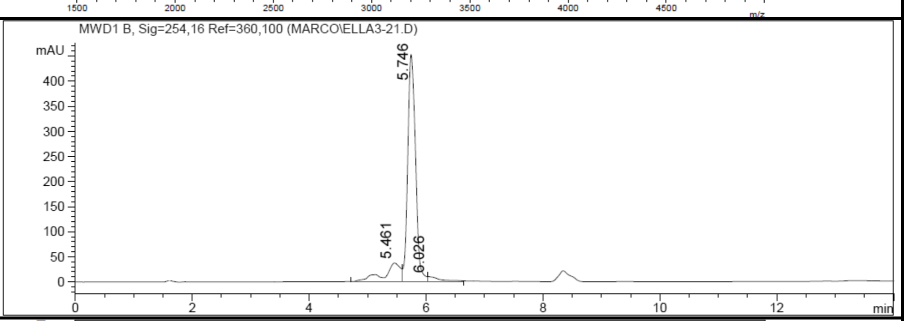
MALDI-MS spectrum	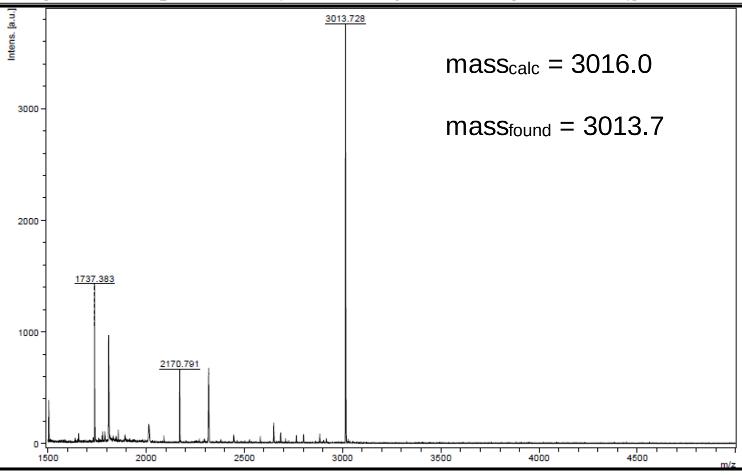

Biological experiments

T4 ligation and amplification by PCR

Table S5 – Sequences of DNA oligonucleotides *I* – *III/III'* and used primer sequences.

DNA	Sequence (5'-3')
HP	CAA ATC CGT TCA SAG GTC GGT GTG AAC GGA TTT GAG TC
I (a-d)	CT*C TCT 7De8a-dGTC T7De8a-dGT 7De-dACC T<br>CT*C TC7De8a-dG 7De-dATT C7De8a-dGC 7De-dACC T<br>CT*C TC7De-dA 7De8a-dG7De-dAT TTC 7De-dACC T<br>CT*C TC7De8a-dG 7De-dA7De8a-dGC CTC 7De-dACCT
I'	TAG G AG GTi aai iaa iAG AGG ACT
II	GTA TCA AGC AG G
II'	TAG GCC TGC TTG
III	CCT ACT CTC GTA TGA CCT CAA CTA CAT GGT CTA CA
III'	TGT AGA CCA TGT AGT TGA GGT CAT ACG AGA G
forward primer	TCG TCG GCA GCG TCA GAT GTG TAT AAG AGA CAG AGG TCG GTG TGA ACG GAT TTG
reverse primer	GTC TCG TGG GCT CGG AGA TGT GTA TAA GAG ACA GTG TAG ACC ATG TAG TTG AGG TCA
forward primer (qPCR)	AGG TCG GTG TGA ACG GAT TTG AG
reverse primer (qPCR)	GTA GAC CAT GTA GTT GAG GTC A

S = C_9-Spacer, T* = Ethynyl-dU, i = inosine; a = abasic site.

5'-phosphorylation of DNA

For 5'-phosphorylation of 280 pmol DNA in a total reaction volume of 20 µL,10 units of T4 polynucleotide kinase (T4 PNK, *Thermo Fisher Scientific*), 1x PNK Buffer A (50 mM Tris-HCl, 10 mM $MgCl_2$, 5 mM DTT, 0.1 mM spermidine, pH = 7.6 at 25 °C, *Thermo Fisher Scientific*) and 1 mM ATP (*Thermo Fisher Scientific*) were used. Reaction mixtures were incubated at 37 °C for 20 min, then heat-inactivated at 75 °C for 15 min and slowly cooled down to 4 °C.

Ligation of DNA

Prior to enzymatic ligation of DNA, the oligonucleotides were annealed by incubation at 85 °C for 10 min and cooling down to 4 °C. For ligation (40 µL scale), 100 pmol of each oligonucleotide, 600 units of T4 DNA Ligase (T4 DNA ligase, *New England Biolabs*) and 1x T4 DNA Ligase Buffer (50 mM Tris-HCl, 10 mM $MgCl_2$, 10 mM DTT, 1 mM ATP, pH = 7.5 at 25 °C, *New England Biolabs*) were mixed. Ligation reactions were performed at 25 °C for 16 h, then stopped by heat inactivation at 75 °C for 15 min and cooled down to 4 °C.

Analysis of DNA ligation

For analysis of DNA ligation reactions, agarose gel electrophoresis was performed using a 3% or 4% agarose gel. Electrophoresis was carried out in TBE buffer (89 mM Tris-borate, 2 mM EDTA, pH = 8.3) at 100 V constant voltage for 15 min and then 150 V constant voltage for about 45 min. For staining of the DNA, Midori Green Direct (*NIPPON Genetics*) and as a reference, GeneRuler Ultra Low Range DNA Ladder (*Thermo Fisher Scientific*) was used. Imaging of the gels was performed using the *Bio-Rad* Gel Doc™ XR system.

Purification of DNA by ethanol precipitation

After the first and second ligation, the DNA was precipitated by adding 1/10 volume of 3 M aq. sodium acetate (pH = 5.2) and 3 volumes of 100% ethanol and incubating this solution for about 4 h or overnight at -80 °C. Afterwards the samples were centrifuged at 4 °C for 30 min (13200 rpm; Centrifuge 5415 R, *Eppendorf*), the supernatant was taken off, additional 100 µL of 100% ethanol were added and the solution was incubated for 1 h at -80 °C. Afterwards the samples were centrifuged at 4 °C for 30 min (13200 rpm; Centrifuge 5415R, *Eppendorf*), the supernatant was taken off, and the DNA pellets were dried at 37 °C. The DNA samples were dissolved in ddH_2O.

Purification of DNA by gel extraction

After the third ligation, the DNA samples were gel extracted using the “QIAquick Gel Extraction Kit” (*Qiagen*) according to the manufacturer protocol.

PCR amplification

Following the third ligation, fully encoded DNA was amplified by PCR. Thereby, 5 µL of gel extracted DNA, 5 U of Taq DNA polymerase (*Thermo Fisher Scientific*), 1x Taq Buffer (10 mM Tris-HCl, 50 mM KCl, 0.08% (v/v) Nonidet P40, pH = 8.8 at 25 °C, *Thermo Fisher Scientific*), 3 mM $MgCl_2$, 0.625 mM of each dNTP (dATP, dGTP, dTTP, and dCTP, corresponding to 2.5 mM of the mixture of dNTPs) and 1 µM of the reverse primer in a reaction volume of 39 µL. The PCR program started with pre-denaturation at 95 °C for 3 min, followed by denaturation for 30 s at 95 °C, annealing for 30 s at 55 °C, and elongation for 30 s at 72 °C. After 10 cycles, 1 µM of the forward primer was added and additional 20 cycles were performed. After PCR, the time for elongation was prolonged to 5 min. The PCR products were analyzed by agarose gel electrophoresis.

Silica-membrane-based purification of DNA

PCR products were purified using the "QIAquick PCR Purification Kit" (*Qiagen*) according to the manufacturer´s protocol.

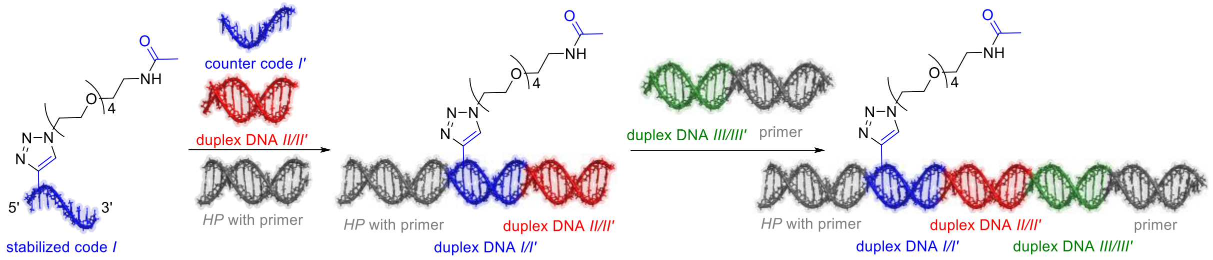

Figure S1 – Encoding scheme for test ligations with chemically stabilized barcodes *I*.

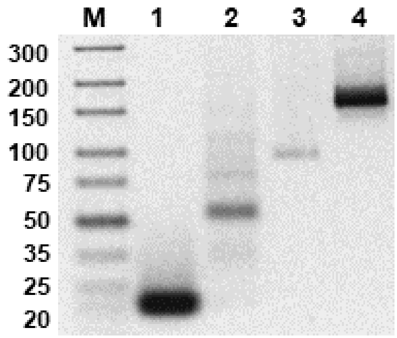

Figure S2 – Agarose gel (4%) of hairpin-based encoding strategy and PCR amplification of fully encoded DNA using stabilized DNA in barcode *I*. Stabilized DNA barcode *I* was ligated to hairpin *HP*, DNA *I'* and to DNA duplexes *II/II'* in one pot to encode the first building block and the heterocyclic scaffold yielding the duplex *HP-I-II/I'-II'*. Then, DNA duplex *HP-I-II/I'-II'* was ligated to DNA duplexes *III/III'* to encode acid building blocks. Lane 1: hairpin *HP*, lane 2: ligation of stabilized DNA barcode *I* to hairpin *HP* and DNA duplexes *II/II'*, lane 3: ligation of DNA duplex *HP-I-II/I'-II'* to DNA duplexes *III/III'*, lane 4: PCR amplification of DNA duplex *HP-I-II-III/I'-II'-III'*.

Sanger sequencing

Sanger sequencing of purified PCR products was performed by *Microsynth Seqlab GmbH* (Göttingen, Germany). The sequencing data was analyzed with *Benchling* [Biology Software] (2021). Retrieved from https://benchling.com.

Table S6 – Sanger sequencing results of the PCR products of encoded sequences containing five different stabilized codes *Ia-e* (T* = Ethynyl-dU)

Code *Ia*	5'-CT*C TCT 7De8a-dG TC T7De8a-dG T 7De-dACC T-3'

forward primer

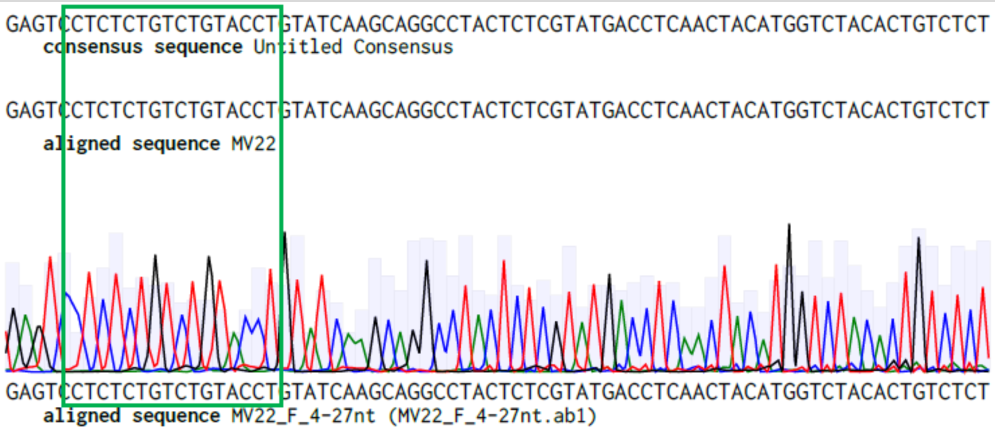

reverse primer

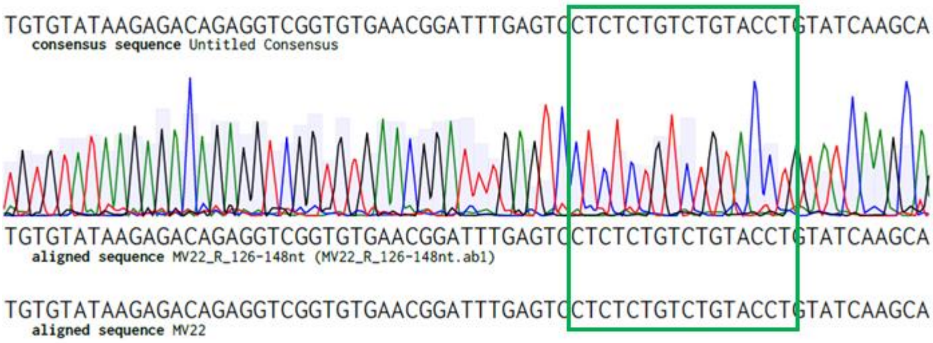

Code *Ib*	5'-CT*C TC7De8a-dG 7De-dATT C7De8a-dG C 7De-dACC T-3'

forward primer

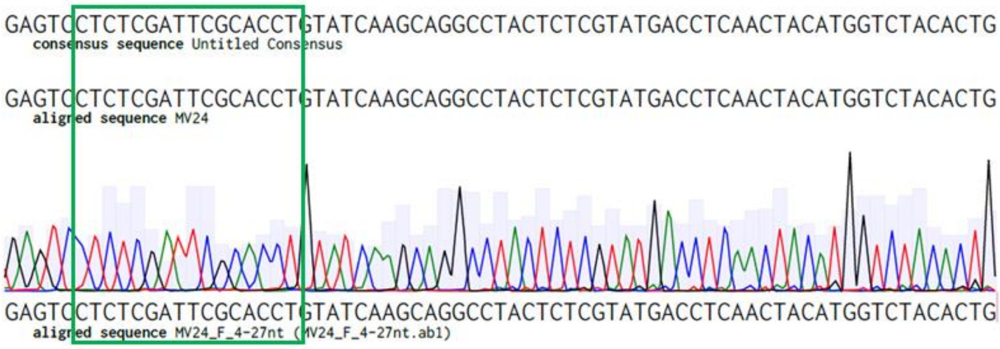

reverse primer

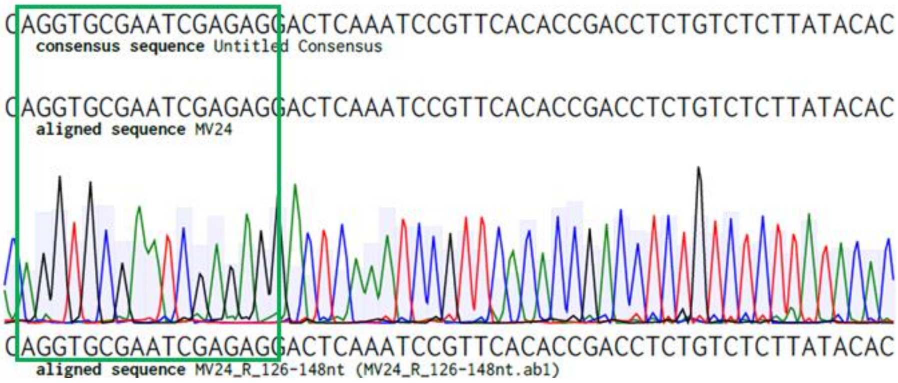

**Code *Id***	**5'-CT*C TC7De-dA 7De8a-dG 7De-dAT TTC 7De-dACC T-3'**

forward primer

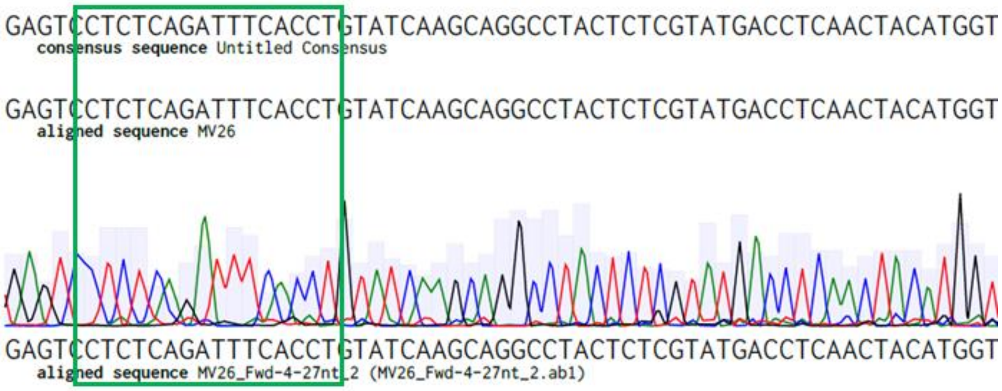

reverse primer

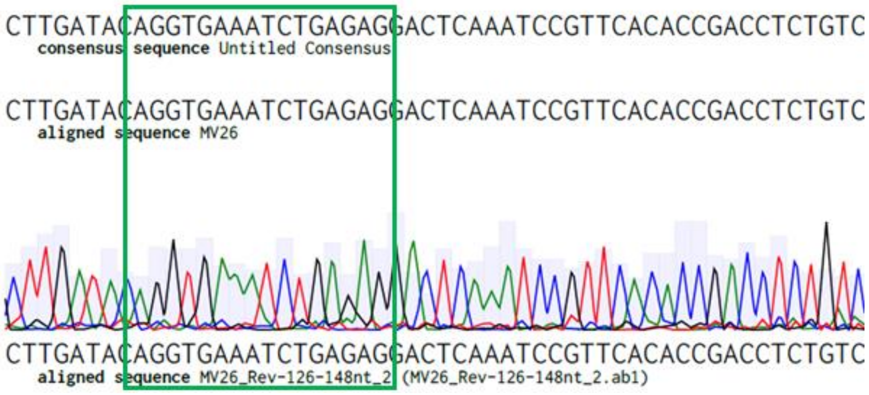

**Code *Ie***	**5'-CT*C TC7De8a-dG 7De-dA7De8a-dG C CTC 7De-dACCT-3'**

forward primer

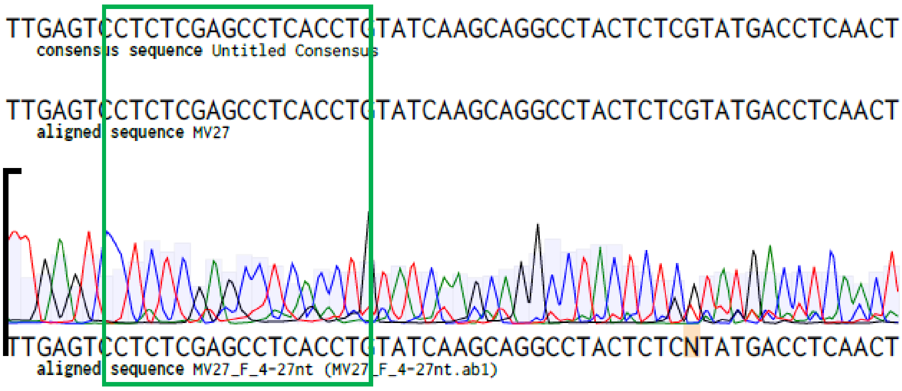

reverse primer

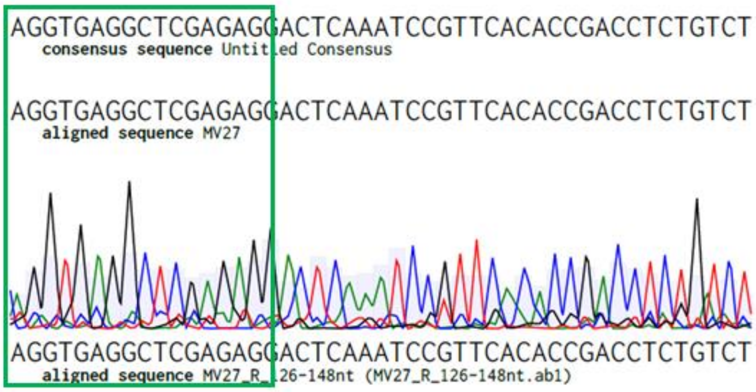

qPCR

For qPCR experiments the following were combined in PCR plate wells (GK480K-50, *Kisker*) in a total volume of 20 µL: DNA template (5 µL, ligation product 3), 200 nM forward primer (0.8 µL, 5 µM stock), 200 nM reverse primer (0.8 µL, 5 µM stock), SsoAdvanced universal SYBR® Green supermix (10 µL, *Bio-Rad*) and H_2O (3.4 µL).

For all qPCR experiments the following amplification method using the LightCycler® 480 II system from *Roche* was performed: hot start at 95 °C for 30 s, then 35 cycles of 95 °C for 15 s (denaturation), 60 °C for 30 s (annealing) and 72 °C for 30 s (elongation).

The specificity of the PCR amplification was analyzed by melting curve measurements. Analysis was done with the LightCycler® 480 – software version 1.5.

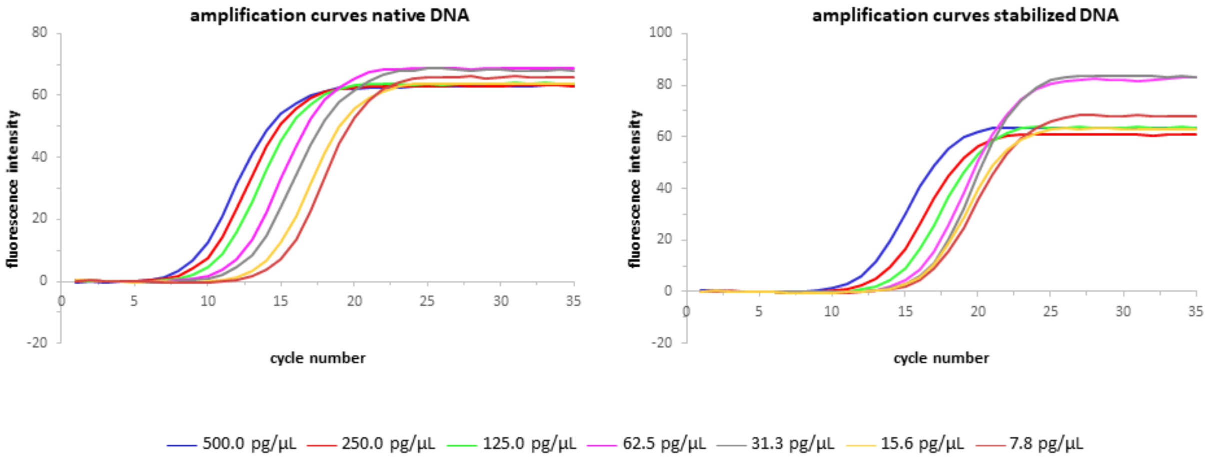

Figure S3 – Amplification curves (qPCR) using different concentrations of DNA sequences containing native or stabilized DNA barcodes *Ia* (5'- CT*C TCT 7De8a-dGTC T7De8a-dGT 7De-dACC T-3').

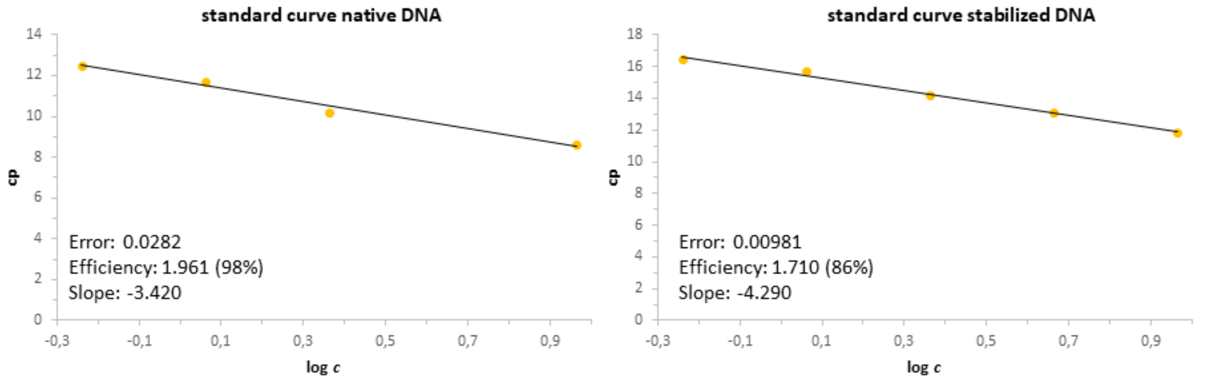

Figure S4 – Standard curve (qPCR) using different concentrations of DNA sequences containing native or stabilized DNA barcodes *Ia* (5'- CT*C TCT 7De8a-dGTC T7De8a-dGT 7De-dACC T-3').

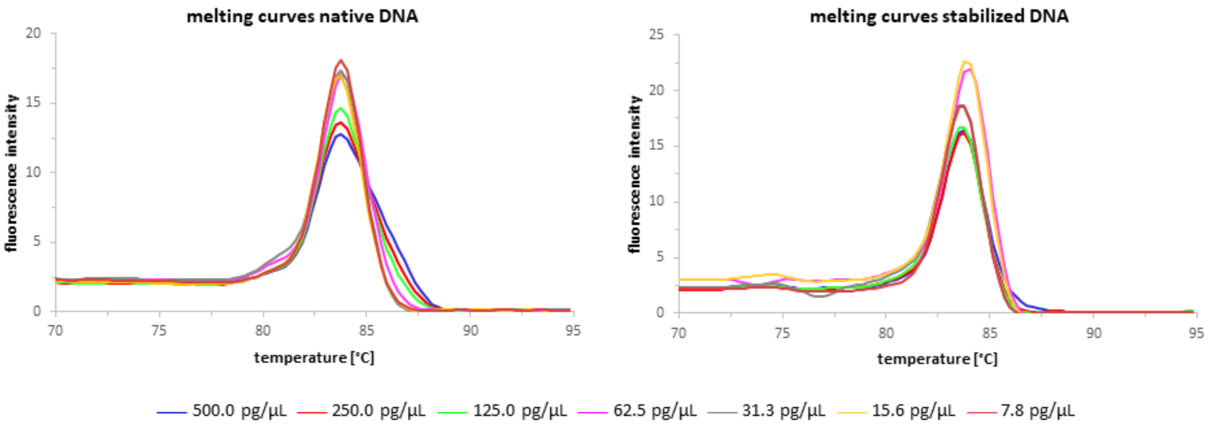

Figure S5 – Melting curves of the PCR products after qPCR of different concentrations of DNA sequences containing native o r stabilized DNA barcodes *Ia* (5'- CT*C TCT 7De8a-dGTC T7De8a-dGT 7De-dACC T-3').

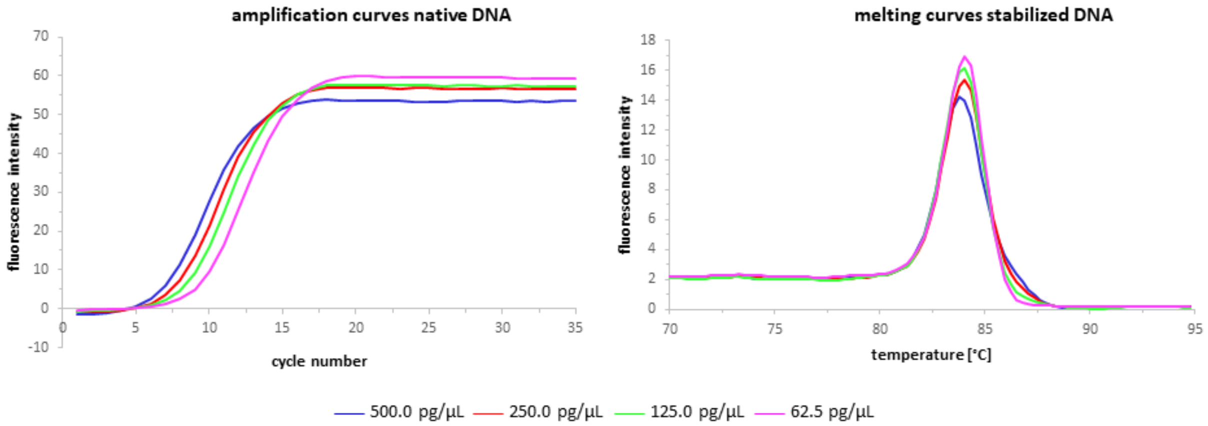

Figure S6 – Amplification and melting curves (qPCR) using different concentrations of DNA sequence containing stabilized DNA barcode *Ib* (5'-CT*C TC7De8a-dG 7De-dATT C7De8a-dGC 7De-dACC T-3').

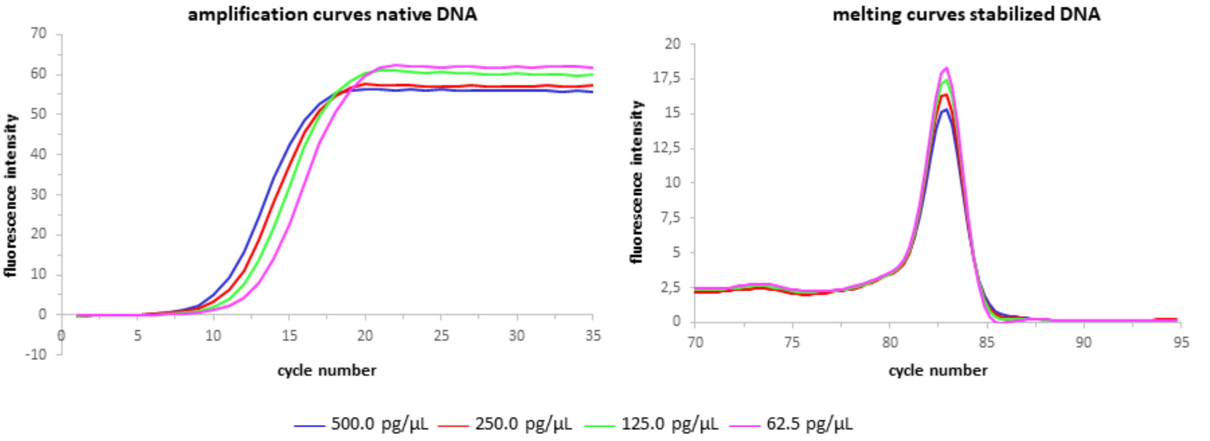

Figure S7 – Amplification and melting curves (qPCR) using different concentrations of DNA sequences containing stabilized DNA barcode *Ic* (5'-CT*C TC7De-dA 7De8a-dG7De-dAT TTC 7De-dACC T-3').

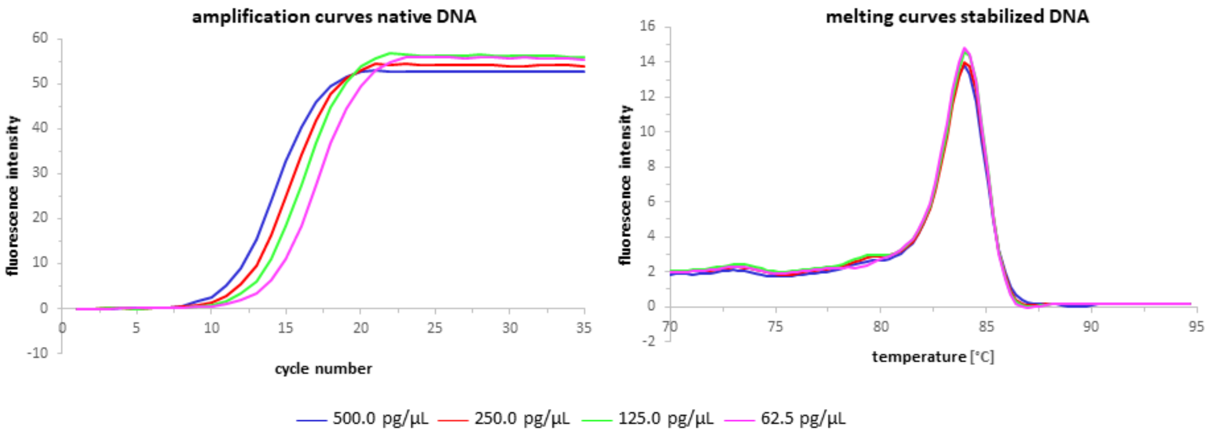

Figure S8 – Amplification and melting curves (qPCR) using different concentrations of DNA sequences containing stabilized DNA barcode *Id* (5'-CT*C TC7De8a-dG 7De-dA7De8a-dGC CTC 7De-dACCT-3').

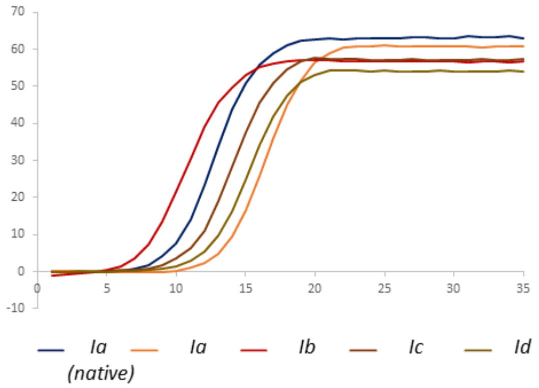

Figure S9 – Differences in the amplification rate of different DNA sequences containing stabilized DNA barcodes *(Ia-d)* and native DNA (*Ia*) [250 pg/µL].

DNA-encoded chemistries
Representative Procedures

The syntheses followed published procedures.[1,18-24]

Copper(I)-promoted alkyne-azide cycloaddition (RP-03)[1]

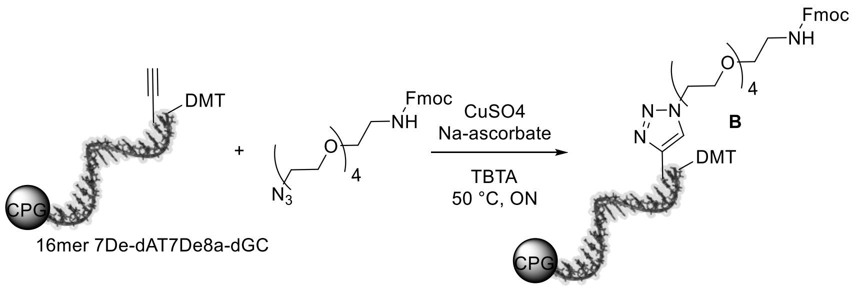

The CPG-bound oligonucleotide-alkyne conjugate (400 nmol) was suspended in 280 µL of H_2O/MeOH (1:1). Subsequently, the azide (33.60 µmol, 84 equiv.) dissolved in 100 µL of DMF, TBTA (16.80 µmol, 42 equiv.) dissolved in 120 µL of DMF, Na-ascorbate (16.80 µmol, 42 equiv.) dissolved in 10 µL of H_2O, and $CuSO_4 \cdot 5H_2O$ (1.68 µmol, 4.2 equiv.) dissolved in 10 µL of H_2O were added to the suspension in this order. Stock solutions of all reactants were prepared before the reaction was started. The reaction mixtures were shaken at 50 °C overnight. Then the CPG-bound conjugate was filtered over a filter column and washed three times with each 200 µL of 0.1 M EDTA solution, 0.1 M $MgCl_2$ solution, water, DMF, MeOH, ACN and CH_2Cl_2 and dried *in vacuo*.

The completeness of the reaction was controlled by cleavage of a small portion (~20 nmol) of CPG-bound oligonucleotide conjugate with 500 µL AMA (AMA = aqueous ammonia (30%)/ aqueous methylamine (40%), 1:1, vol/vol) for 4 h at ambient temperature. To this solution 20 µL of 1 M Tris buffer (pH = 7.5) were added, the mixture was dried in a SpeedVac, and dissolved in 200 µL of distilled water. The crude was analyzed by analytical RP-HPLC and MALDI-MS.

Amide coupling (RP-04)[1]

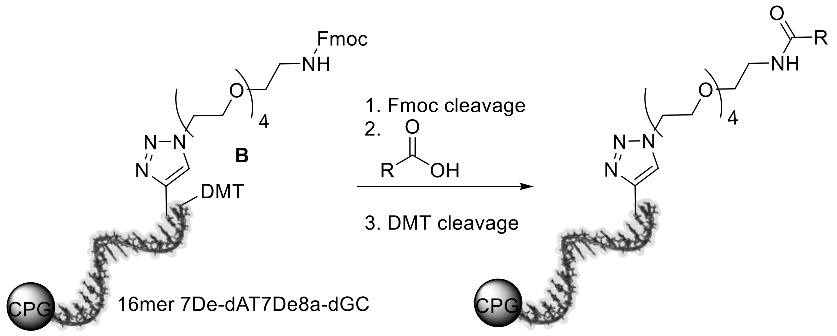

Step 1: The Fmoc-protecting group of the CPG-bound oligonucleotide (250 nmol, 9-10 mg) was cleaved off by addition of 200 µL 20% piperidine in dry DMF and shaking for 5 min. Afterwards, the CPG-bound deprotected oligonucleotide was washed three times with each 200 µL of DMF, MeOH, ACN and CH_2Cl_2 and then dried *in vacuo*.

Step 2: CPG-bound oligonucleotide, carboxylic acid and HATU were dried *in vacuo* for 15 min. Stock solutions of all reactants in dry DMF were prepared before the reaction was started. To the solution of carboxylic acid (25 µmol, 100 equiv.) in 75 µL dry DMF, HATU (25 µmol, 100 equiv.) dissolved in 75 µL dry DMF and DIPEA (62.5 µmol, 250 equiv.) were added. The mixture was shaken for 5 min and added to CPG-bound DNA suspended in 75 µL dry DMF (250 nmol, 1 equiv.). The amide coupling reaction was shaken at ambient temperature for 2 hours. Next, CPG-bound conjugate was filtered over a filter column, washed three times with each 200 µL of DMF, MeOH, ACN and CH_2Cl_2 and dried *in vacuo*. Amide coupling was repeated two times.

Completeness of amide coupling was controlled by cleaving off a small portion of CPG-bound oligonucleotide conjugate (0.7–0.9 mg, ~20 nmol) with 500 µL AMA (AMA = aqueous ammonia (30%)/ aqueous methylamine (40%), 1:1, vol/vol) 1 h (TC-sequences) or 4 h (ATGC- and 7De-dATC-sequences) at ambient temperature. Afterwards 20 µL of 1 M Tris buffer (pH = 7.5) were added, the mixture was dried under reduced pressure (SpeedVac) and DNA was dissolved in 200 µL distilled water. Crude reaction mixture was analyzed by analytical RP-HPLC and MALDI-MS. In case of uncompleted coupling (<90%) the reaction was repeated a third time.

Unreacted amines were capped with acetic anhydride (three times 200 µL, 30 s, 1:1 mixture of THF/methylimidazole, 9:1, vol/vol, and THF/pyridine/acetic anhydride 8:1:1, vol/vol). Capped CPG-bound oligonucleotide conjugate was washed three times with each 200 µL of DMF, MeOH, ACN and CH_2Cl_2 and dried *in vacuo*.

Step 3: DMT-protecting group of CPG-bound oligonucleotide (250 nmol, 9-10 mg of solid phase material) was removed by addition of 200 µL 3% trichloroacetic acid in CH_2Cl_2 for 1 min. Orange coloring of the solution indicated successful removal of protecting group. The deprotection was repeated 3-5 times until no further coloring of the solution was observed. CPG-bound deprotected DNA was washed three times with each 200 µL of 1% TEA in ACN, DMF, MeOH, ACN and CH_2Cl_2 and dried *in vacuo*.

Ugi four-component reaction on CPG-bound oligonucleotides (RP-05)[18]

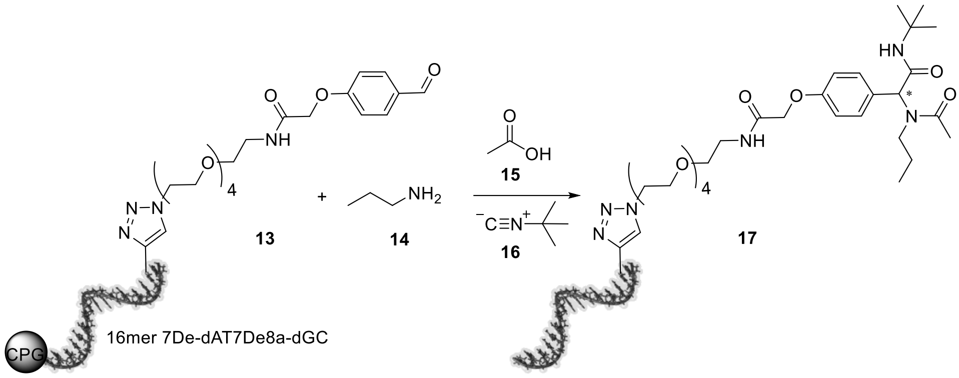

Prior to use, CPG-bound oligonucleotide aldehyde conjugate was dried *in vacuo* for 15 min.[26] A solution of propylamine **14** (1000 equiv., 20 µmol) in 50 µL MeOH was added to the CPG-bound DNA-aldehyde conjugate **13**. The reaction mixture was shaken at ambient temperature for 3 h to effect imine formation. Afterwards, acetic acid **15** (1000 equiv., 20 µmol, solid acids were dissolved in 15 µL MeOH) was pipetted to the reaction mixture, followed by the addition of *tert*-butylisocyanide **16** (1000 equiv., 20 µmol). The reaction mixture was shaken for 16 h at 50 °C. The CPG-bound conjugate was filtered over a filter column, washed three times with each 200 µL of DMF, MeOH, ACN and CH_2Cl_2 and dried *in vacuo*. The CPG-bound DNA conjugate **17** was cleaved from the solid phase and deprotected with 500 µL AMA solution for 4 h at ambient temperature. Afterwards the mixture was dried in a SpeedVac and the remaining DNA pellet was dissolved in 200 µL of distilled water. The crude was analyzed by analytical RP-HPLC and MALDI-MS. The product was isolated by preparative RP-HPLC.

Ugi-azide four-component reaction on CPG-bound oligonucleotides (RP-06)[18]

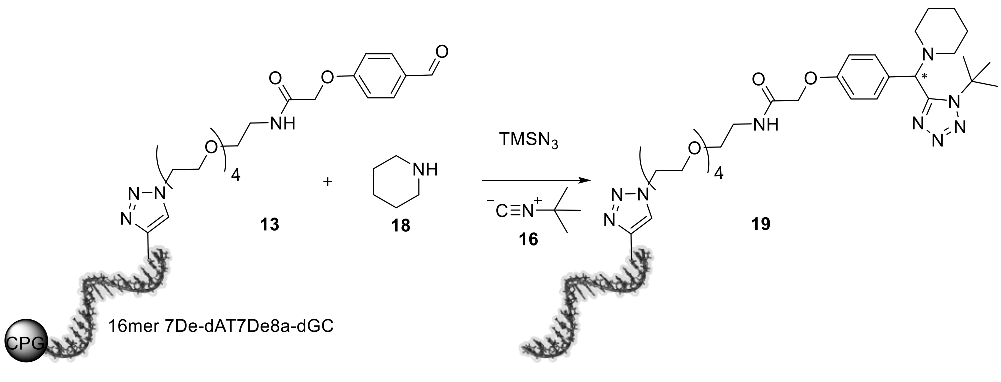

CPG-bound oligonucleotide was dried *in vacuo* for 15 min.[26] A solution of piperidine **18** (1000 equiv., 20 µmol) in 50 µL MeOH was added to the CPG-bound DNA-aldehyde conjugate **13**. The reaction mixture was shaken at ambient temperature for 3 h to effect imine formation. Afterwards, *tert*-butylisocyanide **16** (1000 equiv., 20 µmol) was pipetted to the reaction mixture, followed by the addition of azidotrimethylsilane (1000 equiv., 20 µmol). The

reaction mixture was shaken for 16 h at 50 °C. The CPG-bound conjugate was filtered over a filter column, washed three times with each 200 µL of DMF, MeOH, ACN and CH_2Cl_2 and dried *in vacuo*. The CPG-bound DNA conjugate **19** was cleaved from the solid phase and deprotected with 500 µL AMA solution for 4 h at ambient temperature. Afterwards the mixture was dried in a SpeedVac and the remaining DNA pellet was dissolved in 200 µL of distilled water. The crude was analyzed by analytical RP-HPLC and MALDI-MS. The product was isolated by preparative RP-HPLC.

Groebke-Blackburn-Bienaymé three-component reaction on CPG-bound oligonucleotides (RP-07)[18]

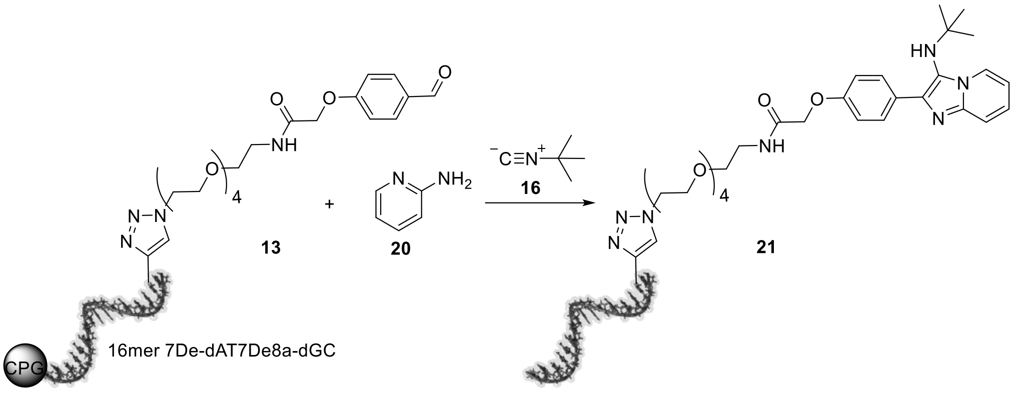

Prior to use, CPG-bound oligonucleotide aldehyde conjugate and 2-aminopyridine **20** were dried *in vacuo* for 15 min.[26] 2-aminopyridine (1000 equiv., 20 µmol) was added to the CPG-bound DNA-aldehyde conjugate **13** in 50 µL MeOH. The reaction mixture was shaken at ambient temperature for 6 h to effect imine formation. Afterwards, *tert*-butylisocyanide **16** (1000 equiv., 20 µmol) was pipetted to the reaction mixture, followed by the addition of acetic acid as Brønsted acid (final volume: 80 µL, acid concentration: 1%). The reaction mixture was shaken for 16 h at ambient temperature. The CPG-bound conjugate was filtered over a filter column, washed three times with each 200 µL of DMF, MeOH, ACN and CH_2Cl_2 and dried *in vacuo*. The CPG-bound DNA conjugate **21** was cleaved from the solid phase and deprotected with 500 µL AMA solution for 4 h at ambient temperature. Afterwards the mixture was dried in a SpeedVac and the remaining DNA pellet was dissolved in 200 µL of distilled water. The crude was analyzed by analytical RP-HPLC and MALDI-MS. The product was isolated by preparative RP-HPLC.

Ugi four-component/aza-Wittig reaction on CPG-bound oligonucleotides (RP-08)[18]

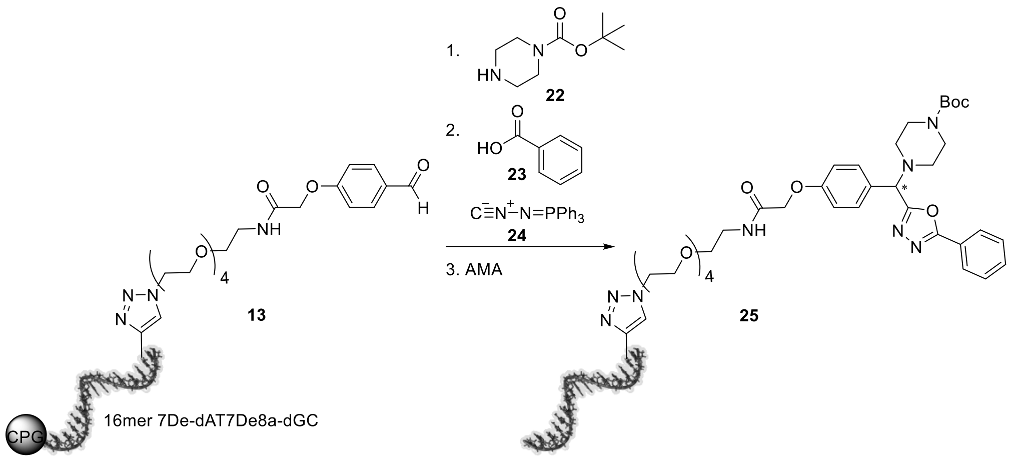

CPG-bound oligonucleotide, *N*-Boc-piperazine **22**, (isocyanoimino)triphenylphosphorane **24** and solid acids were dried *in vacuo* for 15 min.[26] *N*-Boc-piperazine **232** (1000 equiv., 20 µmol) was added to the CPG-bound DNA-aldehyde conjugate **13** in 30 µL 1,2-dichloroethane. The reaction mixture was shaken at ambient temperature for 3 h to effect imine formation. Then, benzoic acid **23** (1000 equiv., 20 µmol) was dissolved in 80 µL 1,2-dichloroethane, transferred to (isocyanoimino)triphenylphosphorane **4**(1000 equiv., 20 µmol) and this mixture was added to the CPG-bound conjugate. The reaction mixture was shaken for 16 h at 50 °C. The CPG-bound conjugate was filtered over a filter column, washed three times with each 200 µL of DMF, MeOH, ACN and CH_2Cl_2 and dried *in vacuo*. The CPG-bound DNA conjugate **25** was cleaved from the solid phase and deprotected with 500 µL AMA solution for 4 h at ambient temperature. Afterwards the mixture was dried in a SpeedVac and the remaining DNA pellet was dissolved in 200 µL of distilled water. The crude was analyzed by analytical RP-HPLC and MALDI-MS. The product was isolated by preparative RP-HPLC.

(*R*)-(-)-BNDHP-mediated Biginelli reaction on CPG-bound oligonucleotides (RP-09)[19]

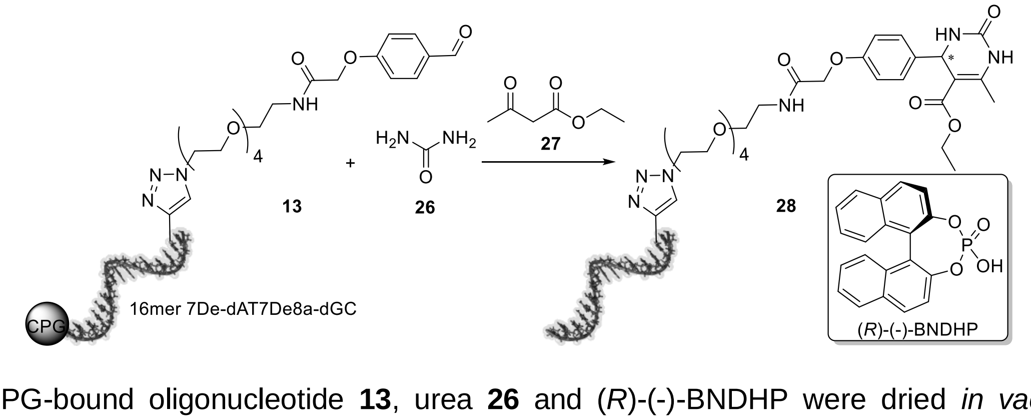

The CPG-bound oligonucleotide **13**, urea **26** and (*R*)-(-)-BNDHP were dried *in vacuo* for 15 min.[27] Urea **6**(10 µmol, 500 equiv.) and (*R*)-(-)-BNDHP (1 µmol, 50 equiv.) were

dissolved both in 30 µL ethanol. The solutions were added to CPG-coupled oligonucleotide-aldehyde conjugate **13** (20 nmol) followed by ethyl acetoacetate **27** (10 µmol, 500 equiv.). The reaction mixture was shaken at 50 °C for 20 h. Then the CPG-bound oligonucleotide conjugate **28** was filtered over a filter column, washed three times with each DMF, MeOH, ACN and CH_2Cl_2 and dried *in vacuo*. CPG-bound oligonucleotide conjugate **28** was cleaved from solid support and deprotected with 500 µL AMA at ambient temperature for 4 h. Afterwards 20 µL of 1 M Tris buffer (pH = 7.5) were added, the mixture was dried under reduced pressure (SpeedVac) and DNA was dissolved in 200 µL distilled water. The crude reaction mixture was analyzed by analytical RP-HPLC and MALDI-TOF-MS. The product was purified by preparative RP-HPLC.

(*R*)-(-)-BNDHP-mediated Povarov reaction on CPG-bound oligonucleotides (RP-10)[19]

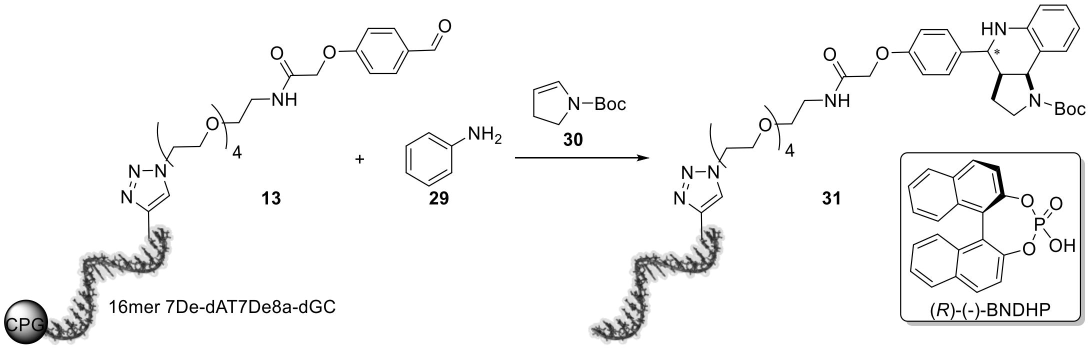

Prior to use, CPG-bound oligonucleotide, solid anilines and (*R*)-(-)-BNDHP were dried *in vacuo* for 15 min.[27] Aniline **29** (10 µmol, 500 equiv.) was dissolved in 24 µL ethanol. The solution was added to CPG-bound oligonucleotide-aldehyde conjugate **13** (20 nmol) suspended in 12 µL triethyl orthoformate. The suspension was shaken at ambient temperature for 4 h. Afterwards 30 µL of (*R*)-(-)-BNDHP (2 µmol, 100 equiv.) in ethanol followed by *N*-Boc-2,3-dihydro-1H-pyrrole **30** (10 µmol, 500 equiv.) was added. The reaction mixture was shaken at 50 °C for 16 h. Then the CPG-bound oligonucleotide conjugate **31** was filtered over a filter column, washed three times with each DMF, MeOH, ACN and CH_2Cl_2 and dried *in vacuo*. CPG-bound oligonucleotide conjugate **31** was cleaved from solid support and deprotected with 500 µL AMA at ambient temperature for 4 h. Afterwards 20 µL of 1 M Tris buffer (pH = 7.5) were added, the mixture was dried under reduced pressure (SpeedVac) and DNA was dissolved in 200 µL distilled water. The crude reaction mixture was analyzed by analytical RP-HPLC and MALDI-TOF-MS. The product was purified by preparative RP-HPLC.

TFA-mediated Pictet-Spenger reaction on CPG-bound oligonucleotides (RP-11)[20,21]

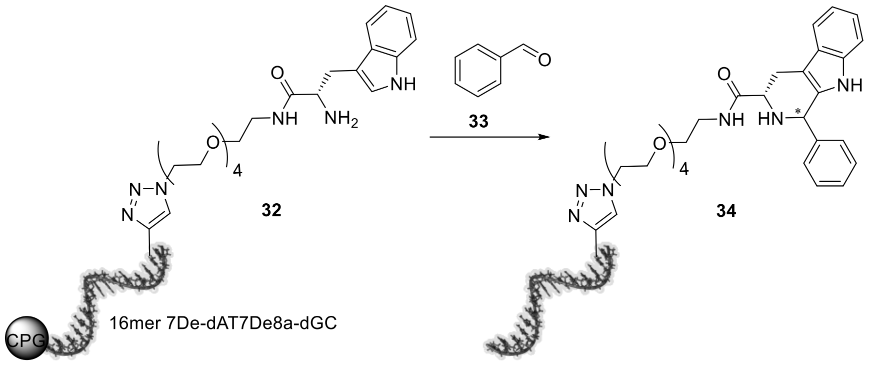

Prior to use, CPG-bound oligonucleotide **32** was dried *in vacuo* for 15 min.[28,29] Benzaldehyde **33** (30 µmol, 1500 equiv.) was dissolved in 50 µL of a 5% trifluoroacetic acid in CH_2Cl_2 solution. This solution was added to CPG-bound oligonucleotide-tryptophan conjugate **32** (20 nmol) and the reaction mixture was shaken at ambient temperature for 20 h. Afterwards CPG bound DNA was filtered over a filter column, washed with excess of 1% TEA and three times with each 200 µL of DMF, MeOH, ACN and CH_2Cl_2 and dried in vacuo. CPG-bound oligonucleotide conjugate **34** was cleaved from solid support and deprotected with 500 µL AMA at ambient temperature for 4 h. Afterwards 20 µL of 1 M Tris buffer (pH = 7.5) were added, the mixture was dried under reduced pressure (SpeedVac) and DNA was dissolved in 200 µL distilled water. The crude reaction mixture was analyzed by analytical RP-HPLC and MALDI-TOF-MS. The product was purified by preparative RP-HPLC.

Cu(I)/bpy-mediated Petasis reaction on CPG-bound oligonucleotides (RP-12)[22]

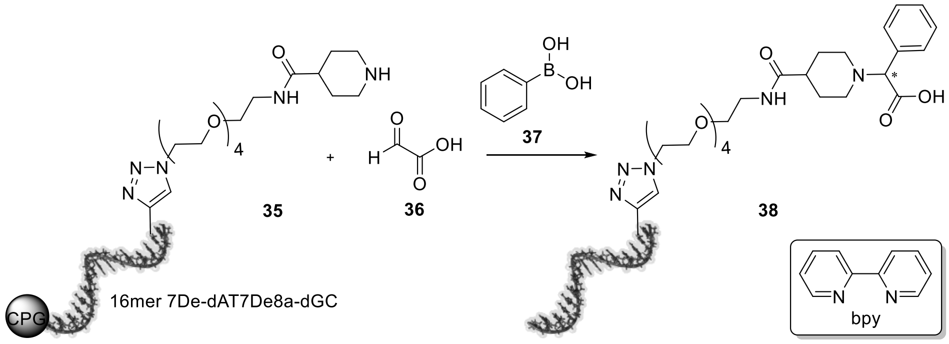

Prior to use all solid materials were dried in vacuo for 30 min.[30] CuCl (4.0 µmol, 200 equiv., 40 mM calculated for the final volume of 100 µL) and 2,2′-bipyridine (bpy, 4.0 µmol, 200 equiv., 40 mM calculated for the final volume of 100 µL) were dissolved in 48 µL DMF. The solution was shaken at 50 °C for 1 h. Phenylboronic acid **37** (50 µmol, 2500 equiv., 500 mM calculated for the final volume of 100 µL) were dissolved in the CuCl/bpy solution in DMF. 12 µL triethyl orthoformate and glyoxylic acid **36** (40 µmol, 2000 equiv., 400 mM

calculated for the final volume of 100 µL) dissolved in 40 µL DMF were added. The solution was added to CPG-coupled-DNA-secondary amine conjugate **35** (20 nmol, 1 equiv.) and the suspension was shaken at 50 °C for 20 h. Then the CPG-bound DNA conjugate was filtered over a filter column, washed three times with each 200 µL of 0.1 M EDTA solution, 0.1 M $MgCl_2$ solution, water, DMF, MeOH, ACN and CH_2Cl_2 and dried in vacuo. CPG-bound oligonucleotide conjugated *α*-aryl glycine **38** were cleaved from solid support and deprotected with 500 µL AMA for 4 h at ambient temperature. Afterwards 20 µL of 1 M Tris buffer (pH = 7.5) were added, the mixture was dried under reduced pressure (SpeedVac) and the DNA was dissolved in 200 µL distilled water. The crude reaction mixture was analyzed by analytical RP-HPLC and MALDI-TOF-MS. The product was purified by preparative RP-HPLC.

Zn(II)-mediated *aza*-Diels-Alder reaction on CPG-bound oligonucleotides (RP-13)[19]

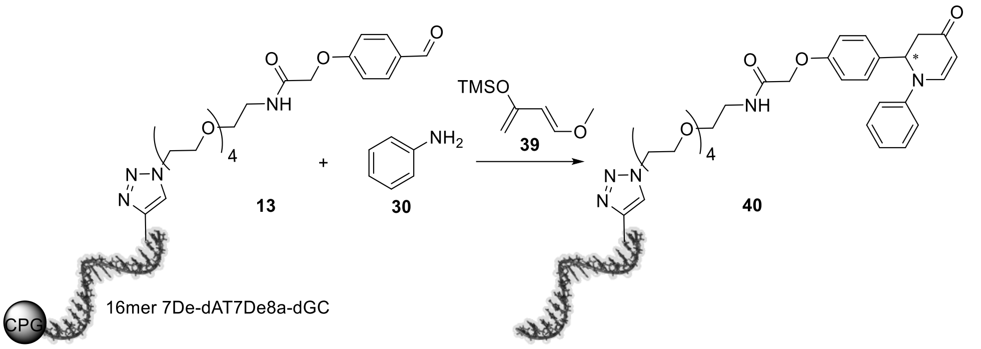

CPG-bound oligonucleotide **13**, and $ZnCl_2$ were dried *in vacuo* for 15 min.[27] Aniline **30** (10 µmol, 500 equiv.) was dissolved in 24 µL acetonitrile. The solution was added to CPG-coupled oligonucleotide-aldehyde conjugate **13** (20 nmol) suspended in 12 µL triethyl orthoformate. The suspension was shaken at ambient temperature for 4 h. Afterwards 30 µL of $ZnCl_2$ (2 µmol, 100 equiv.) in ACN followed by Danisheskys's diene **40** (20 µmol, 1000 equiv.) was added. The reaction mixture was shaken for 1 h at ambient temperature. Then the CPG-coupled oligonucleotide conjugate **41** was filtered over a filter column, washed three times with each 200 µL of 0.1 M EDTA solution, 0.1 M $MgCl_2$ solution, water, DMF, MeOH, ACN and CH_2Cl_2 and dried *in vacuo*. CPG-coupled oligonucleotide conjugate **38** was cleaved from solid support and deprotected with 200 µL aqueous ammonia (30%) at 50 °C for 6 h. Afterwards 20 µL of 1 M Tris buffer (pH = 7.5) were added, the mixture was dried under reduced pressure (SpeedVac) and DNA was dissolved in 200 µL distilled water. The crude reaction mixture was analyzed by analytical RP-HPLC and MALDI-TOF-MS. The product was purified by preparative RP-HPLC.

Yb(III)-mediated Castagnoli-Cushman reaction on CPG-bound oligonucleotides (RP-14)[21]

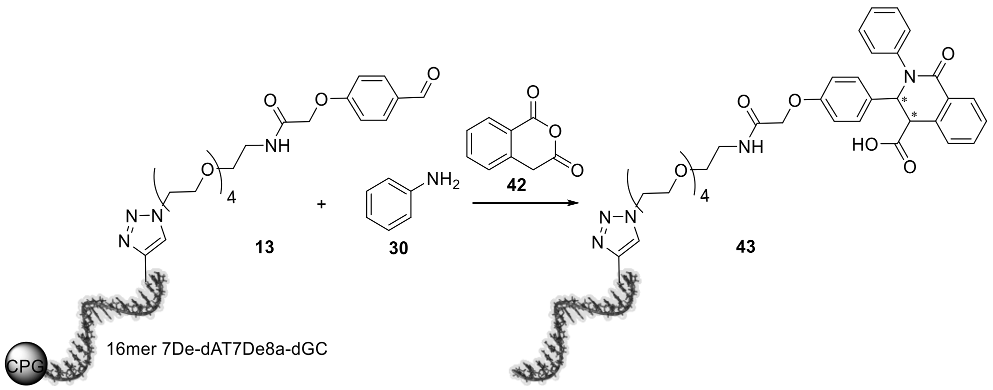

CPG-bound oligonucleotide **13**, homophtalic anhydride **42** and $Yb(OTf)_3$ were dried *in vacuo* for 15 min. [28] Aniline **30** (10 µmol, 500 equiv.) was dissolved in 24 µL CH_2Cl_2. The solution was added to the CPG-bound oligonucleotide-aldehyde conjugate **13** (20 nmol) suspended in 12 µL triethyl orthoformate. The suspension was shaken at ambient temperature for 4 h. Afterwards 30 µL of a suspension of $Yb(OTf)_3$ (1 µmol, 50 equiv.) in CH_2Cl_2 was added, followed by 30 µL of a suspension of homophtalic anhydride **42** (10 µmol, 500 equiv.) in CH_2Cl_2. Prior addition to the reaction vessel both suspensions were vortexed and pipetted up and down to obtain homogeneous suspensions. The reaction mixture was shaken for 1 h at ambient temperature. Then the CPG-bound conjugate was filtered over a filter column and washed three times with each 200 µL of 0.1 M EDTA solution, 0.1 M $MgCl_2$ solution, water, DMF, MeOH, ACN and CH_2Cl_2 and dried in *vacuo*. CPG-bound oligonucleotide conjugate **43** was then cleaved from the solid support and deprotected with 500 µL AMA solution for 4 h at ambient temperature. To this solution 20 µL of 1 M Tris buffer (pH = 7.5) were added, the mixture was dried in a SpeedVac and afterwards dissolved in 200 µL of distilled water. The crude was analyzed by analytical RP-HPLC and MALDI-MS. The product was purified by preparative RP-HPLC.

Yb(PFO)$_3$-mediated three-component synthesis of pyrazoles on CPG-bound oligonucleotides (RP-15)[1]

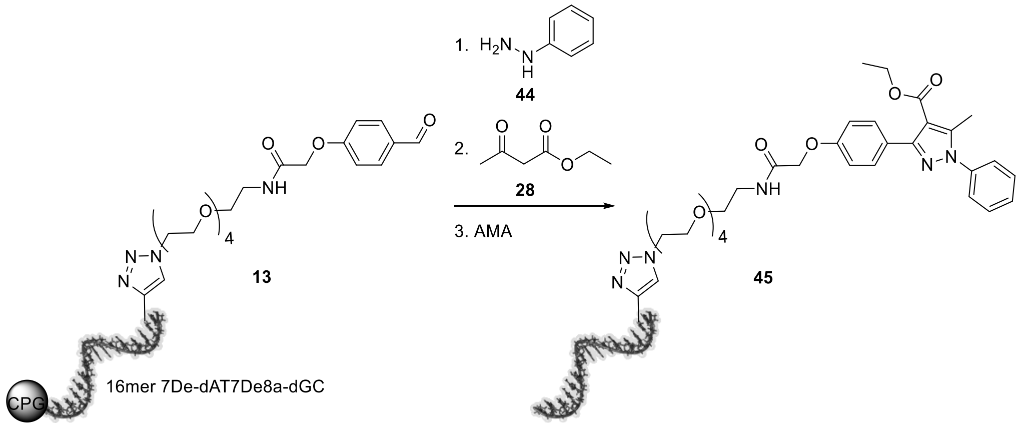

The catalyst Yb(PFO)$_3$ was prepared according to a published procedure.[31] Prior to the reaction, the hydrazine was extracted with diluted NH_3 solution and CH_2Cl_2, dried over $MgSO_4$ and finally dried *in vacuo* if the hydrazine was present as a hydrochloride salt. The hydrazine **44** (250 equiv., 5 µmol), dissolved in 30 µL toluene was added to the CPG-bound DNA-aldehyde conjugate **13** and the reaction mixture was shaken at ambient temperature for 0.5 h. Afterwards, ethyl acetoacetate **28** (3000 equiv., 60 µmol) and 50 µL of a suspension of Yb(PFO)$_3$ (250 equiv., 5 µmol) in toluene was added. Prior addition to the reaction vessel the Yb(PFO)$_3$ suspension was vortexed and pipetted up and down to obtain a homogeneous suspension. The reaction mixture was shaken at 50 °C for 16 h. The CPG-bound conjugate was filtered over a filter column and washed with each 3x 200 µL of 0.1 M EDTA solution, 0.1 M $MgCl_2$ solution, water, DMF, MeOH, ACN and CH_2Cl_2 and then dried *in vacuo*. The CPG-bound DNA conjugate **45** was cleaved from the solid phase and deprotected by adding 500 µL AMA solution and shaking for 1 h (TC-sequences) or 4 hours (ATCG- and 7De-dATC-sequences) at ambient temperature. Afterwards the mixture was dried in a SpeedVac and the remaining DNA pellet was dissolved in 200 µL of distilled water.

Au(I)/Ag(I)-promoted pyrazoline-containing spiroheterocycle synthesis on CPG-bound oligonucleotides (RP-16)[23]

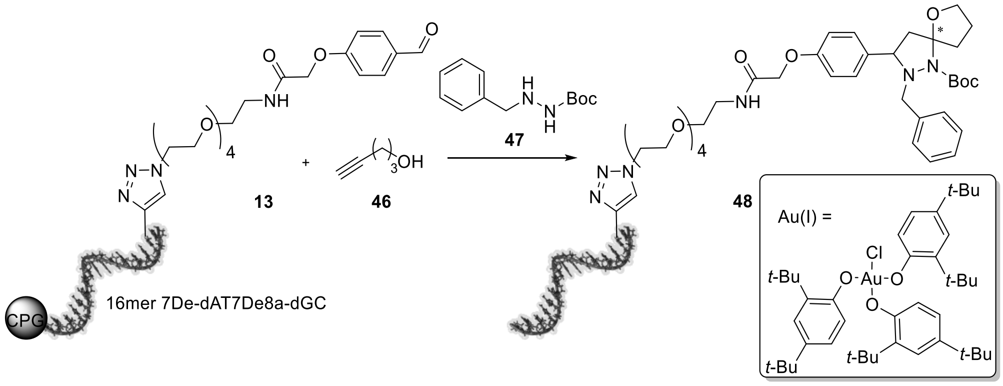

CPG-bound oligonucleotide **13**, *tert*-butyl 2-benzylhydrazinecarboxylate **47**, [Tris(2,4-di-*tert*-butylphenyl)phosphite]gold chloride and $AgSbF_6$ were dried *in vacuo* for 15 min.[32] The solution of *tert*-butyl 2-benzylhydrazine-carboxylate **47** (500 equiv., 15 µmol) in 20 µL THF and pent-4-yn-1-ol **46** (1000 equiv., 30 µmol) were added to CPG- bound DNA-aldehyde conjugate **13** (30 nmol) followed by equimolar mixture of Au(I)/$AgSbF_6$ (250 equiv., 7.5 µmol) suspended in 30 µL THF. Prior addition to the reaction vessel the mixture was vortexed and pipetted up and down. The reaction mixture was shaken at room temperature for 20 h. Then the CPG-bound conjugate was filtered over a filter column and washed three times with each 200 µL of 0.1 M EDTA solution, 0.1 M $MgCl_2$ solution, water, DMF, MeOH, ACN and CH_2Cl_2 and dried in *vacuo*. CPG-bound oligonucleotide conjugate **48** was then cleaved from the solid support and deprotected with 500 µL AMA solution for 4 h at ambient temperature. To this solution 20 µL of 1 M Tris buffer (pH = 7.5) were added, the mixture was dried in a SpeedVac and afterwards dissolved in 45 µL of distilled water. 5 µL of 1,3,5-triazine-2,4,6-trithiol trisodium salt solution (15% in H_2O) were added and the solution was shaken for 30 min at ambient temperature. Afterwards the sample was centrifuged at 4 °C for 30 min (13200 rpm; Centrifuge 5415 R, *Eppendorf*), the supernatant was taken off and diluted with 5 µL of a 3 M sodium acetate (pH = 5.2) and 200 µL 100% ethanol. The solution was incubated overnight at -80 °C. Afterwards the samples were centrifuged at 4 °C for 30 min (13200 rpm; Centrifuge 5415 R, *Eppendorf*), the supernatant was taken off, additional 100 µL of 100% ethanol were added to the pellet and the solution was incubated again for 1 h at -80 °C. Afterwards the sample was centrifuged at 4 °C for 30 min (13200 rpm; Centrifuge 5415 R, *Eppendorf*), the supernatant was taken off, and the DNA pellets were dried at 37 °C. The DNA samples were dissolved in 100 µL ddH_2O. The crude was analyzed by analytical RP-HPLC and MALDI-MS. The product was purified by preparative RP-HPLC.

Au(I)/Ag(I)-promoted pyrazoline synthesis on CPG-bound oligonucleotides (RP-17)[20]

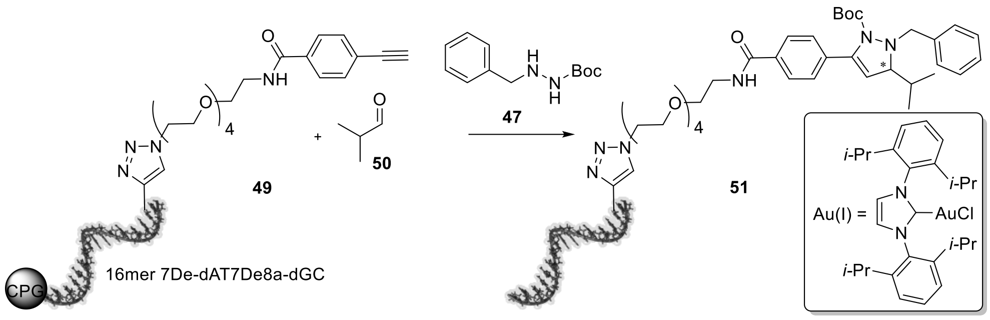

CPG-bound oligonucleotide **49**, *tert*-butyl 2-benzylhydrazinecarboxylate **47**, chloro[1,3-bis(2,6-diisopropylphenyl)imidazol-2-ylidene] gold(I) and AgOTf were dried *in vacuo* for 15 min.[29] The solution of *tert*-butyl 2-benzylhydrazine-carboxylate **47** (1000 equiv., 20 µmol) in 20 µL dry acetonitrile and aliphatic aldehyde **50** (1000 equiv., 20 µmol) were added to CPG-bound DNA-alkyne conjugate **49** (20 nmol) followed by equimolar mixture of Au(I)/AgOTf (250 equiv., 5 µmol) suspended in dry 30 µL acetonitrile. Prior addition to the reaction vessel the mixture was vortexed and pipetted up and down. The reaction mixture was shaken at room temperature for 20 h. Then the CPG-bound conjugate was filtered over a filter column and washed three times with each 200 µL of 0.1 M EDTA solution, 0.1 M $MgCl_2$ solution, water, DMF, MeOH, ACN and CH_2Cl_2 and dried *in vacuo*. CPG-bound oligonucleotide conjugate **51** was then cleaved from the solid support and deprotected with 500 µL AMA solution for 4 h at 50 °C. To this solution 20 µL of 1 M Tris buffer (pH = 7.5) were added, the mixture was dried in a SpeedVac and afterwards dissolved in 45 µL of distilled water. 5 µL of 1,3,5-triazine-2,4,6-trithiol trisodium salt solution (15% in H_2O) were added and the solution was shaken for 30 min at ambient temperature. Afterwards the sample was centrifuged at 4 °C for 30 min (13200 rpm; Centrifuge 5415 R, *Eppendorf*), the supernatant was taken off and diluted with 5 µL of a 3 M sodium acetate (pH = 5.2) and 200 µL 100% ethanol. The solution was incubated overnight at -80 °C. Afterwards the samples were centrifuged at 4 °C for 30 min (13200 rpm; Centrifuge 5415 R, *Eppendorf*), the supernatant was taken off, additional 100 µL of 100% ethanol were added to the pellet and the solution was incubated again for 1 h at -80 °C. Afterwards the sample was centrifuged at 4 °C for 30 min (13200 rpm; Centrifuge 5415 R, Eppendorf), the supernatant was taken off, and the DNA pellets were dried at 37 °C. The DNA samples were dissolved in 100 µL ddH_2O. The crude was analyzed by analytical RP-HPLC and MALDI-MS. The product was purified by preparative RP-HPLC.

Au(I)/Ag(I)-promoted pyrazole synthesis on CPG-bound oligonucleotides (RP-18)[20]

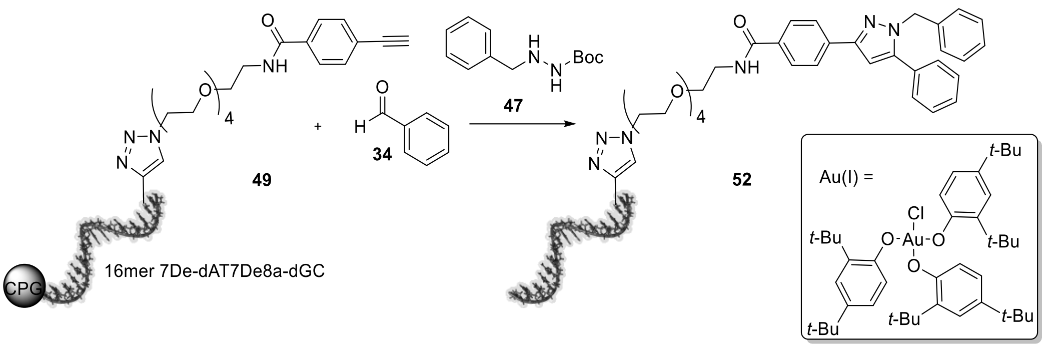

CPG-bound oligonucleotide **49**, *tert*-butyl 2-benzylhydrazinecarboxylate **47**, [tris(2,4-di-tert-butylphenyl)phosphite]gold chloride and AgOTf were dried *in vacuo* for 15 min.[29] The solution of *tert*-butyl 2-benzylhydrazine-carboxylate **47** (1000 equiv., 20 µmol) in 20 µL glacial acetic acid and benzaldehyde **34** (1000 equiv., 20 µmol) were added to CPG- bound DNA-alkyne conjugate **49** (20 nmol) followed by equimolar mixture of Au(I)/AgOTf (250 equiv., 5 µmol) suspended in dry 30 µL glacial acetic acid. Prior addition to the reaction vessel the mixture was vortexed and pipetted up and down. The reaction mixture was shaken at 60 °C for 20 h. Then the CPG-bound conjugate was filtered over a filter column and washed three times with each 200 µL of 0.1 M EDTA solution, 0.1 M $MgCl_2$ solution, water, DMF, MeOH, ACN and CH_2Cl_2 and dried in *vacuo*. CPG-bound oligonucleotide conjugate **52** was then cleaved from the solid support and deprotected with 500 µL AMA solution for 4 h at ambient temperature. To this solution 20 µL of 1 M Tris buffer (pH = 7.5) were added, the mixture was dried in a SpeedVac and afterwards dissolved in 45 µL of distilled water. 5 µL of 1,3,5-triazine-2,4,6-trithiol trisodium salt solution (15% in H_2O) were added and the solution was shaken for 30 min at ambient temperature. Afterwards the sample was centrifuged at 4 °C for 30 min (13200 rpm; Centrifuge 5415 R, *Eppendorf*), the supernatant was taken off and diluted with 5 µL of a 3 M sodium acetate (pH = 5.2) and 200 µL 100% ethanol. The solution was incubated overnight at -80 °C. Afterwards the samples were centrifuged at 4 °C for 30 min (13200 rpm; Centrifuge 5415 R, *Eppendorf*), the supernatant was taken off, additional 100 µL of 100% ethanol were added to the pellet and the solution was incubated again for 1 h at -80 °C. Afterwards the sample was centrifuged at 4 °C for 30 min (13200 rpm; Centrifuge 5415 R, *Eppendorf*), the supernatant was taken off, and the DNA pellets were dried at 37 °C. The DNA samples were dissolved in 100 µL ddH_2O. The crude was analyzed by analytical RP-HPLC and MALDI-MS. The product was purified by preparative RP-HPLC.

Trifluoroacetic acid-mediated Boc cleavage on oligonucleotide scaffold conjugates in solution (RP-19)

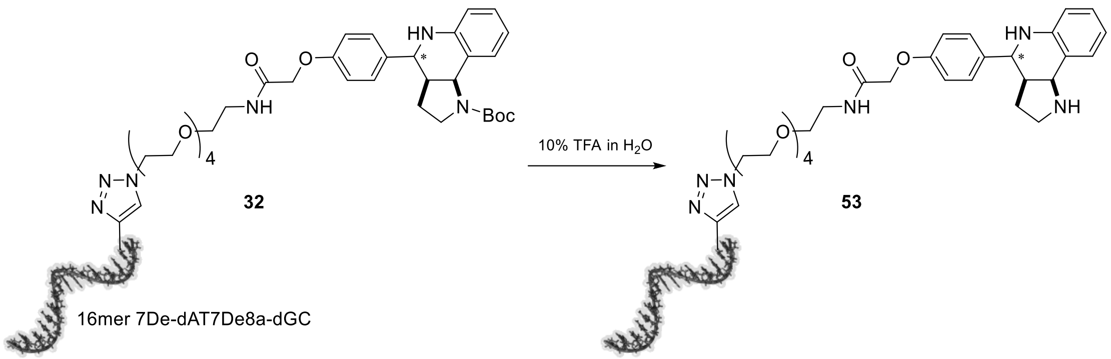

An isolated pellet of Boc-protected oligonucleotide conjugate **32** was dissolved in 20 µL of a 10% trifluoroacetic acid in H_2O solution. The solution was shaken at ambient temperature for 4 h. The Boc deprotected oligonucleotide conjugate **53** was precipitated by adding 2 µL of a 3 M sodium acetate (pH = 5.2) and 80 µL of 100% ethanol and storing this solution for overnight at -80 °C. Afterwards, the samples were centrifuged at 4 °C for 30 min (13200 rpm; Centrifuge 5415 R, *Eppendorf*), the supernatant was taken off and the DNA pellets were dried. Oligonucleotide conjugate was dissolved in ddH_2O and analyzed by analytical RP-HPLC and MALDI-TOF-MS.

Table S7 – Overview of diverse chemical reactions on CPG-bound stabilized barcode.

Entry	Reaction	Conditions
1	Ugi four-component reaction (**RP-05**)[18]<br>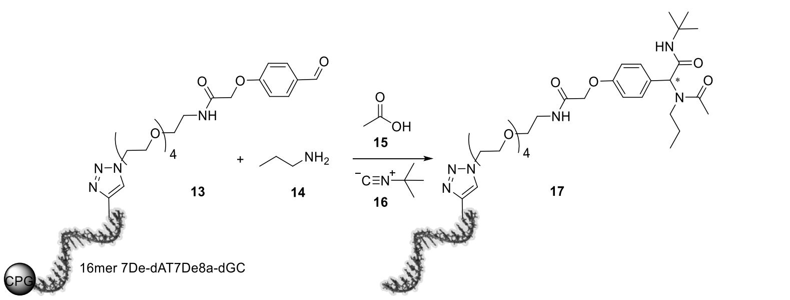	1. MeOH, 50 °C<br>2. AMA, rt
2	Ugi-azide four-component reaction (**RP-06**)[18]<br>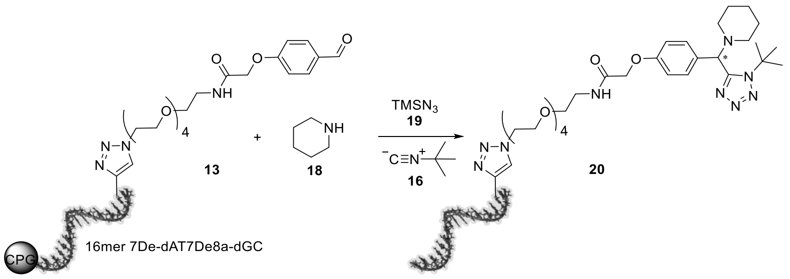	1. MeOH, 50 °C<br>2. AMA, rt
3	Groebke-Blackburn-Bienaymé reaction (**RP-07**)[18]<br>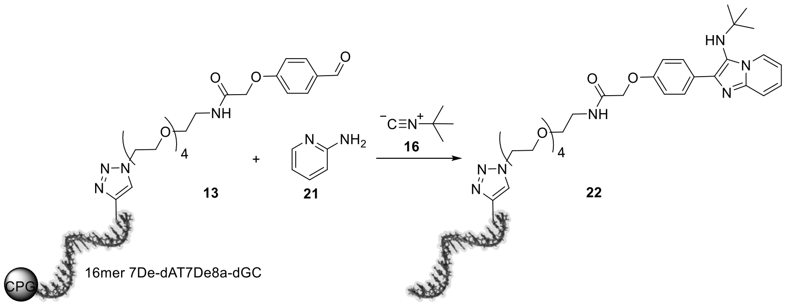	1. 1% acetic acid in MeOH, rt<br>2. AMA, rt
4	Ugi/*aza*-Wittig reaction (**RP-08**)[18]<br>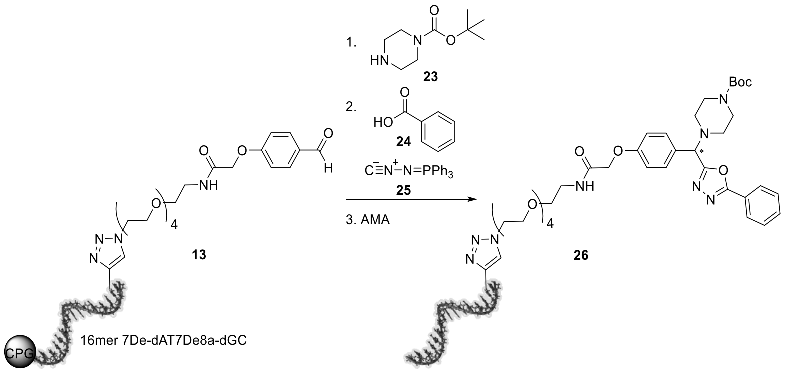	1. MeOH, 50 °C<br>2. AMA, rt
5	Biginelli reaction (**RP-09**)[19]<br>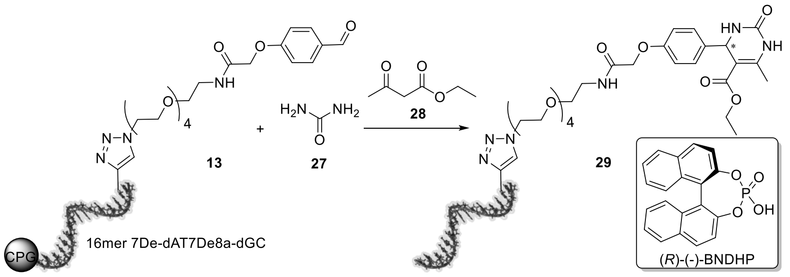	1. (*R*)-(-)-BNDHP EtOH 50 °C<br>2. AMA, rt

6 Povarov reaction (**RP-10**)[19]

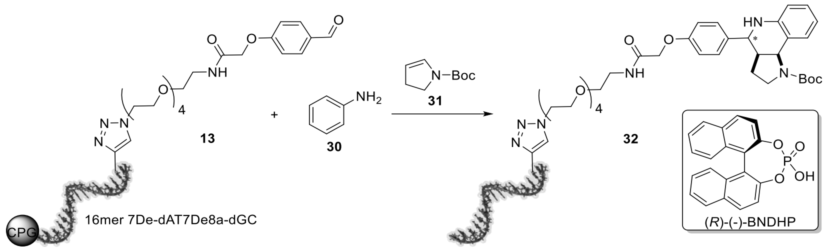

1. (*R*)-(-)-BNDHP
   EtOH/TEOF, 50 °C
2. AMA, rt

6 Pictet-Spengler reaction (**RP-11**)[20,21]

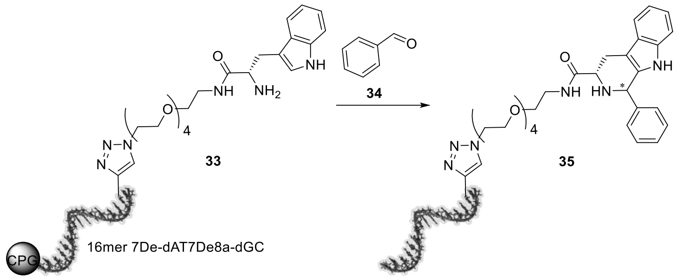

1. TFA
   CH_2Cl_2, rt
2. AMA, rt

7 Petasis reaction (**RP-12**)[22]

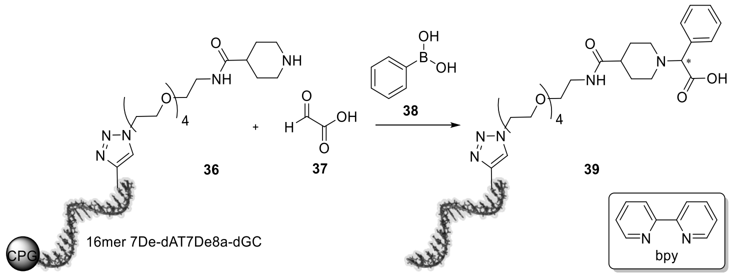

1. CuCl/bpy
   DMF/TEOF, 50 °C
2. AMA, rt

8 aza-Diels-Alder reaction (**RP-13**)[19]

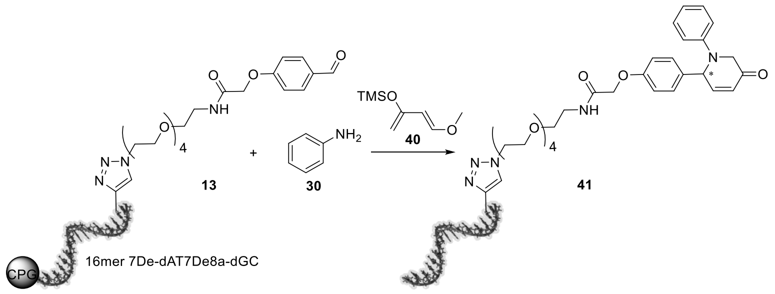

1. $ZnCl_2$
   ACN/TEOF, rt
2. aq. NH_3, 50 °C

9 Castagnoli-Cushman reaction (**RP-14**)[21]

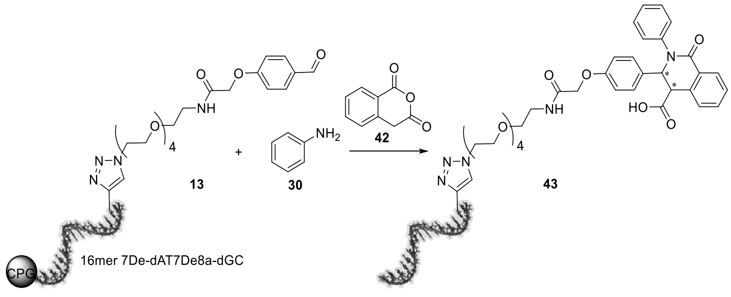

1. $Yb(OTf)_3$
   CH_2Cl_2/TEOF,
   rt
2. AMA, rt

10 Three-component pyrazole synthesis (**RP-15**)[1]

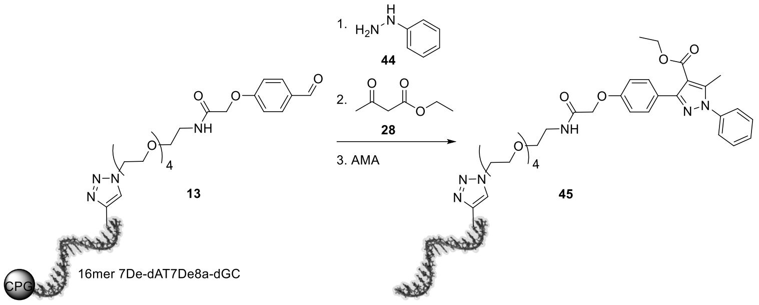

1. Yb(PFO)3
   toluene, 50 °C
2. AMA, rt

11 Pyrazoline-containing spiroheterocycle synthesis (**RP-16**)[23]

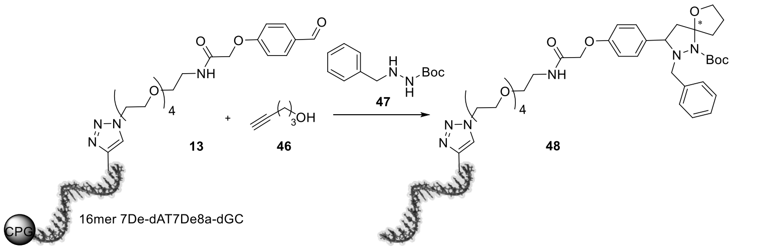

1. Au(I)/Ag(I)
   THF, rt
2. AMA, rt

12 Pyrazoline synthesis (**RP-17**)[20]

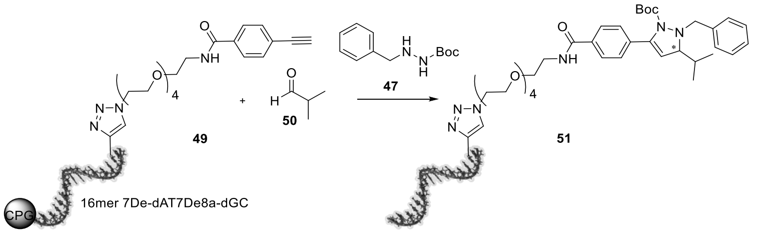

1. Au(I)/Ag(I)
   MeCN, 50 °C
2. AMA, rt

13 Pyrazole synthesis (**RP-18**)[20]

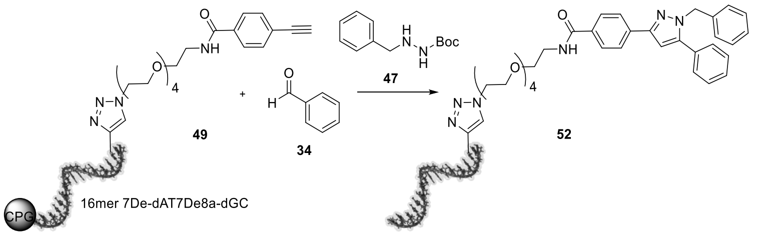

1. Au(I)/Ag(I)
   AcOH, 60 °C
2. AMA, rt

HPLC traces and MALDI-MS spectrum

CPG-bound DNA-starting material conjugates

CPG-bound 16mer 7De-dAT7De8a-dGC-alkyne conjugate was reacted with Boc-*N*-amido-PEG(4)-azide according to RP-03.

HPLC trace of crude reaction mixture (analytical RP-HPLC (**II**))

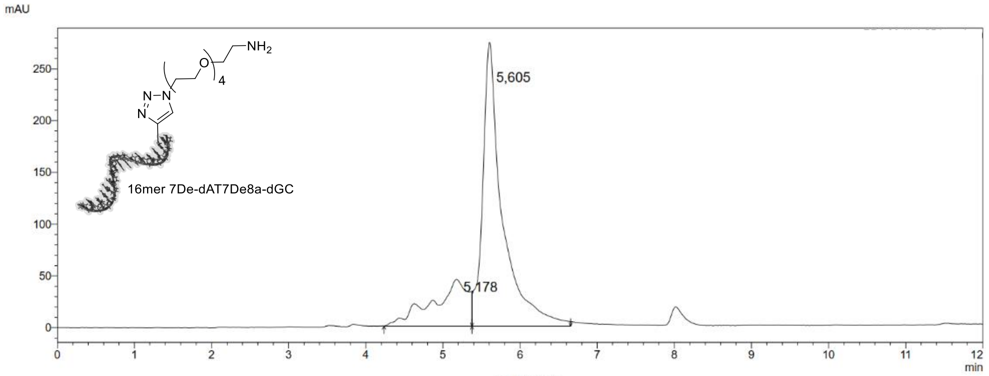

Peak Table

PDA Ch1 254nm

Peak#	Ret. Time	Area	Area%	Height	Height%
1	5,178	1442433	22,991	45280	14,180
2	5,605	4831386	77,009	274032	85,820
Total		6273820	100,000	319311	100,000

MALDI-MS spectrum of crude reaction mixture

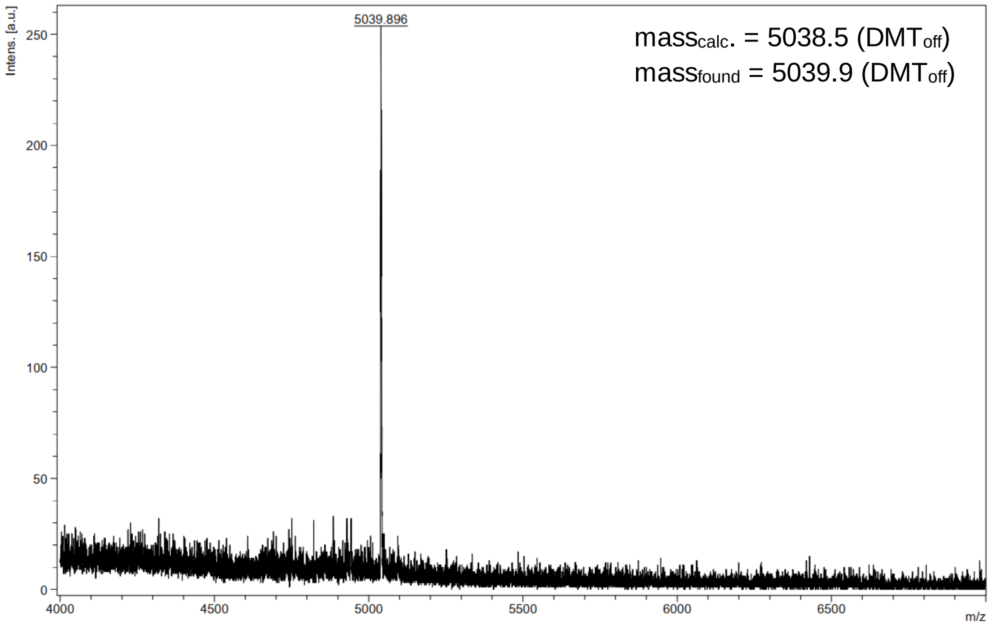

DNA conjugate 13: CPG-bound 16mer 7De-dAT7De8a-dGC-PEG(4)-NH_2 conjugate was reacted with 4-formyl-phenoxyacetic acid according to RP-04.

HPLC trace of crude reaction mixture **13** (analytical RP-HPLC (I))

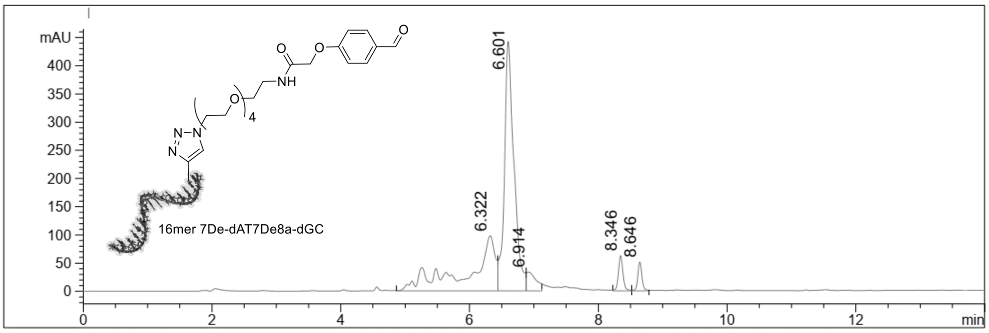

Peak list:

Ret. Time	Width min	Height	Area	Area %
6.322	0.478	97.310	2792.504	34.022
6.601	0.171	442.552	4537.662	55.283
6.914	0.172	33.106	342.080	4.168
8.346	0.082	62.429	306.150	3.730
8.646	0.075	51.345	229.603	2.797

MALDI-MS spectrum of crude reaction mixture **13**

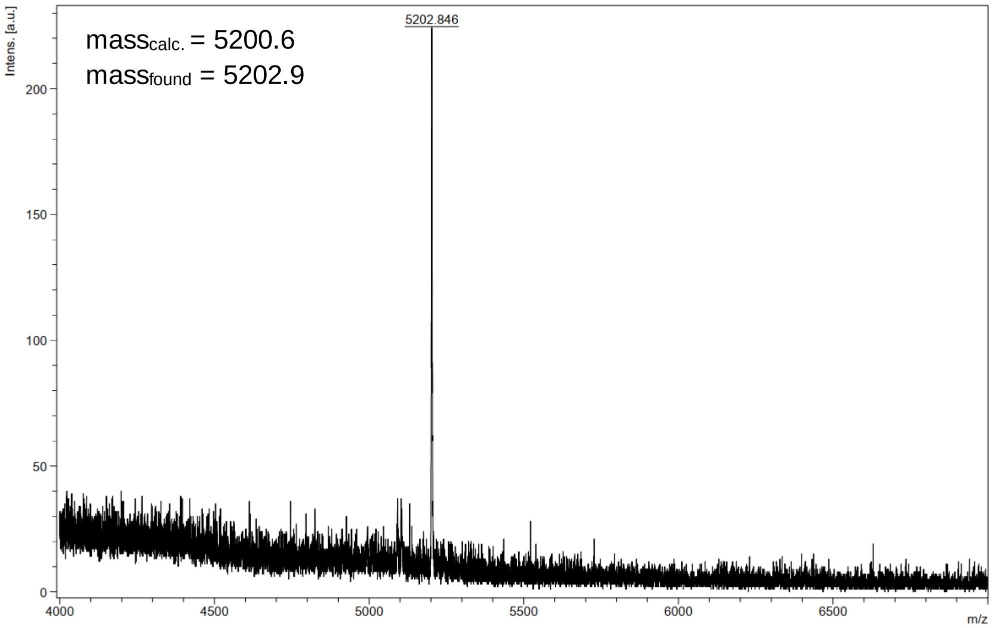

DNA conjugate 33: CPG-bound 16mer 7De-dAT7De8a-dGC-PEG(4)-NH_2 conjugate was reacted with *N*-Boc-tryptophan according to RP-04.

HPLC trace of crude reaction mixture **33** (analytical RP-HPLC (I))

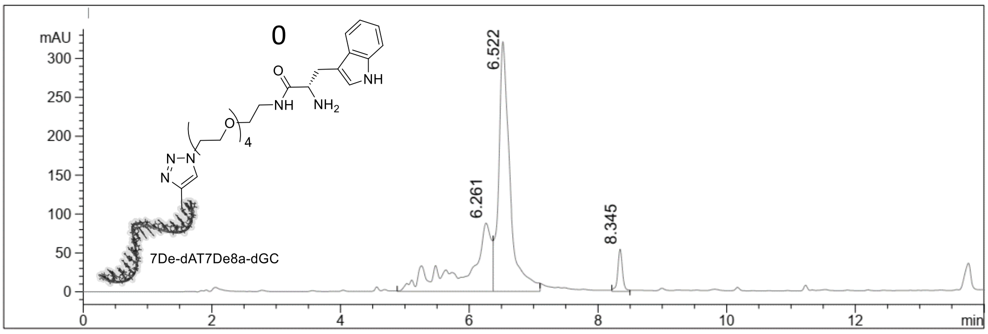

Peak list:

Ret. Time	Width min	Height	Area	Area %
6.261	0.453	87.893	2390.263	36.372
6.522	0.202	321.298	3903.339	59.395
8.345	0.085	54.279	278.175	4.233

MALDI-MS spectrum of crude reaction mixture **33**

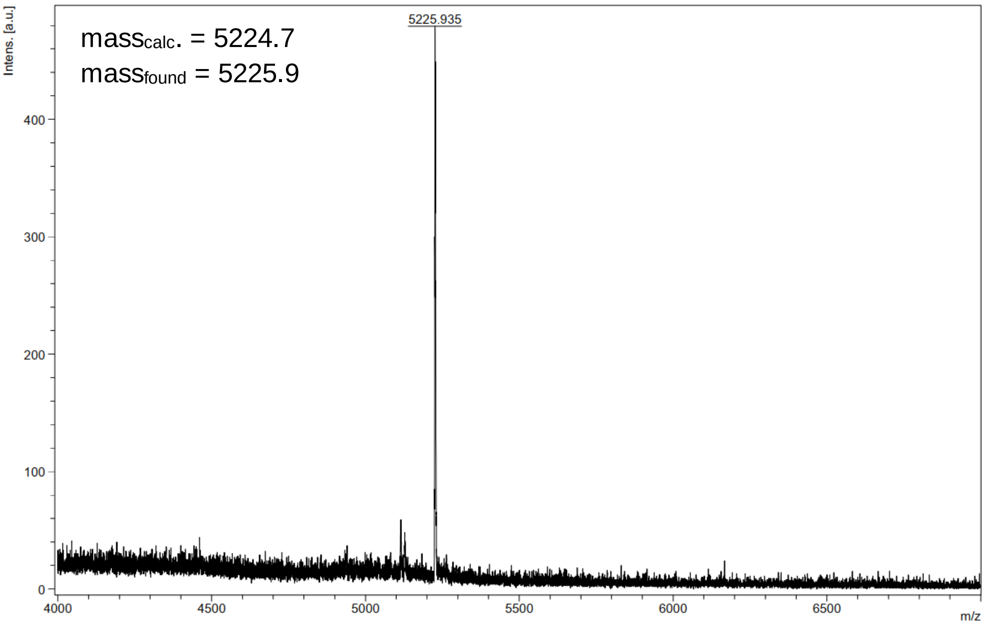

DNA conjugate 36: CPG-bound 16mer 7De-dAT7De8a-dGC-PEG(4)-NH_2 conjugate was reacted with *N*-Fmoc-piperidine-4-carboxylic acid according to RP-04.

HPLC trace of crude reaction mixture **36** (analytical RP-HPLC (**I**))

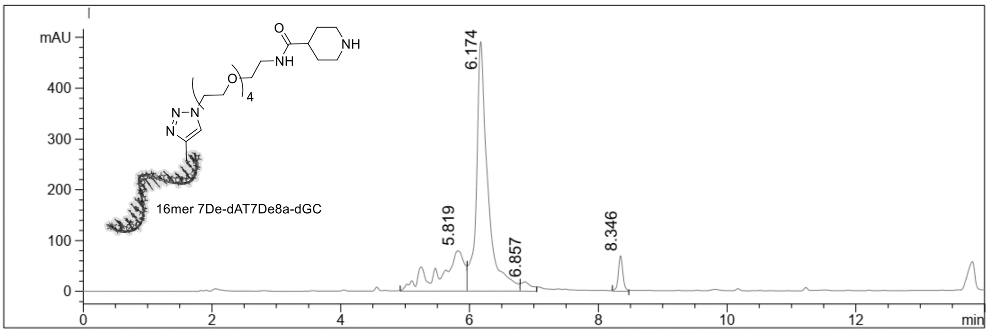

Peak list:

Ret. Time	Width min	Height	Area	Area %
5.819	0.458	79.293	2177.890	24.994
6.174	0.203	491.300	5982.634	68.659
6.857	0.186	18.320	204.946	2.352
8.346	0.083	69.812	348.089	3.995

MALDI-MS spectrum of crude reaction mixture **36**

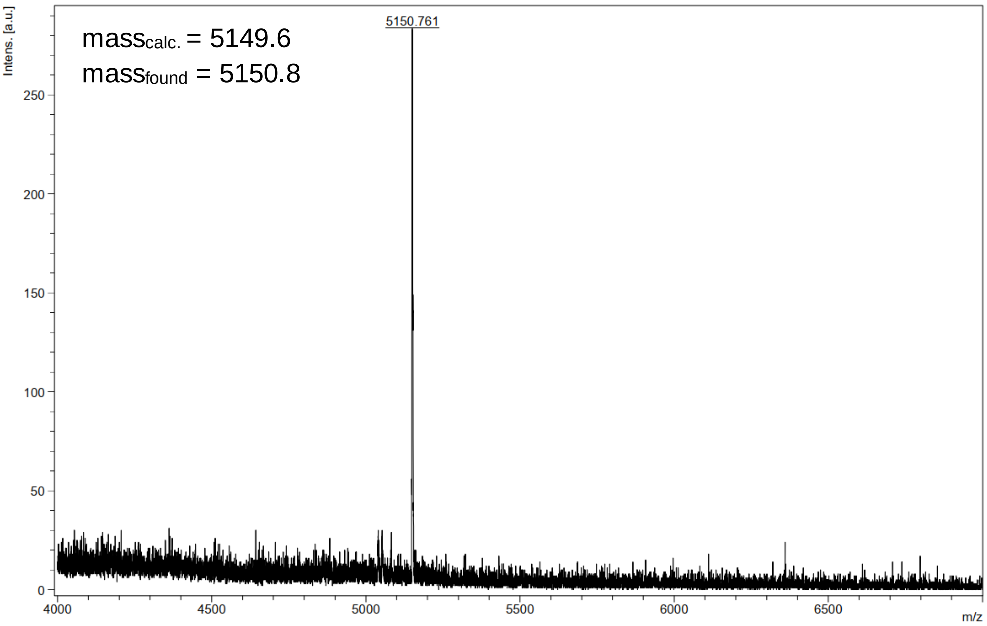

DNA conjugate 49: CPG-bound 16mer 7De-dAT7De8a-dGC-PEG(4)-NH_2 conjugate was reacted with 4-ethynylbenzoic acid according to RP-04.

HPLC trace of crude reaction mixture **49** (analytical RP-HPLC (**I**))

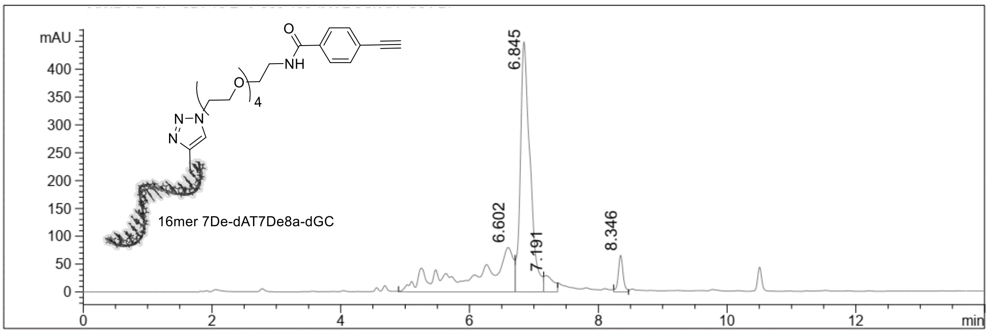

Peak list:

Ret. Time	Width min	Height	Area	Area %
6.602	0.685	79.704	3276.362	38.013
6.845	0.174	449.181	4702.889	54.564
7.191	0.170	29.253	298.181	3.460
8.346	0.086	65.993	341.531	3.963

MALDI-MS spectrum of crude reaction mixture **49**

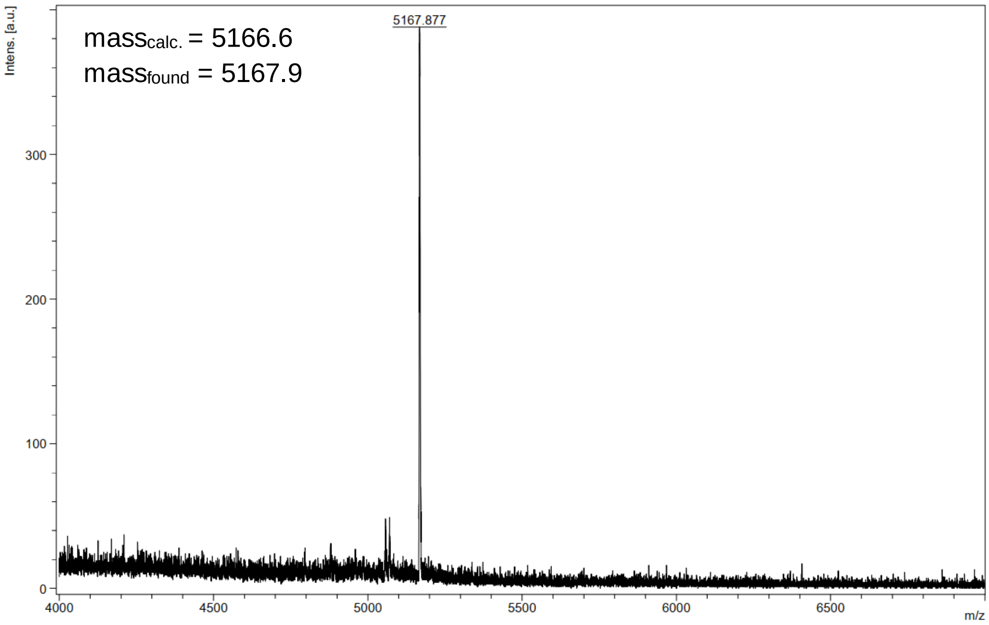

Isocyanide multicomponent reactions

Ugi four-component reaction

DNA conjugate 17: CPG-bound 16mer 7De-dAT7De8a-dGC-aldehyde conjugate **13** was reacted with acetic acid **15**, propylamine **14** and *tert*-butylisocyanide **16** according to RP-05.

HPLC trace of crude reaction mixture **17** (analytical RP-HPLC (**I**))

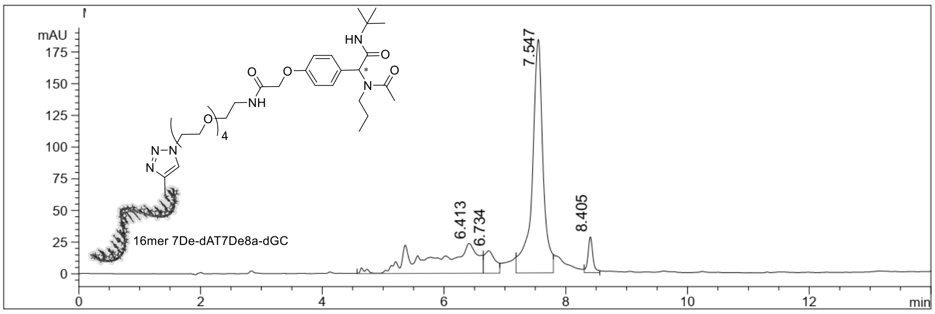

Peak list:

Ret. Time	Width min	Height	Area	Area %
6.413	0.870	23.574	1230.667	31.645
6.734	0.203	17.763	216.507	5.567
7.547	0.207	184.342	2284.406	58.741
8.405	0.092	28.378	157.346	4.046

HPLC trace of isolated product **17** (analytical RP-HPLC (I))

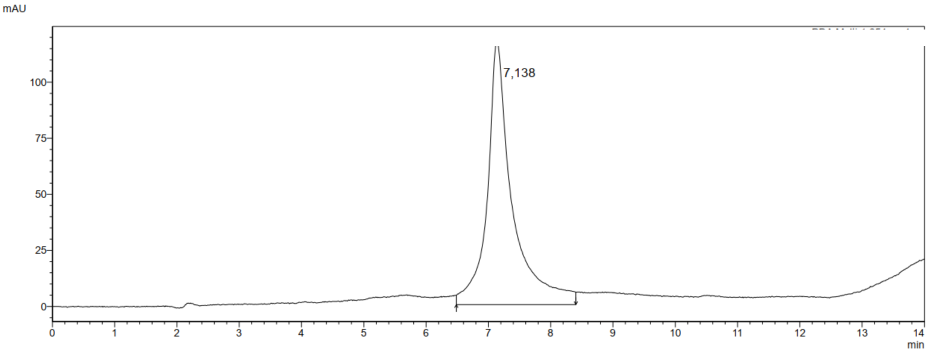

Peak Table

PDA Ch1 254nm

Peak#	Ret. Time	Area	Area%	Height	Height%
1	7,138	3267026	100,000	118029	100,000
Total		3267026	100,000	118029	100,000

MALDI-MS spectrum of isolated product **17**

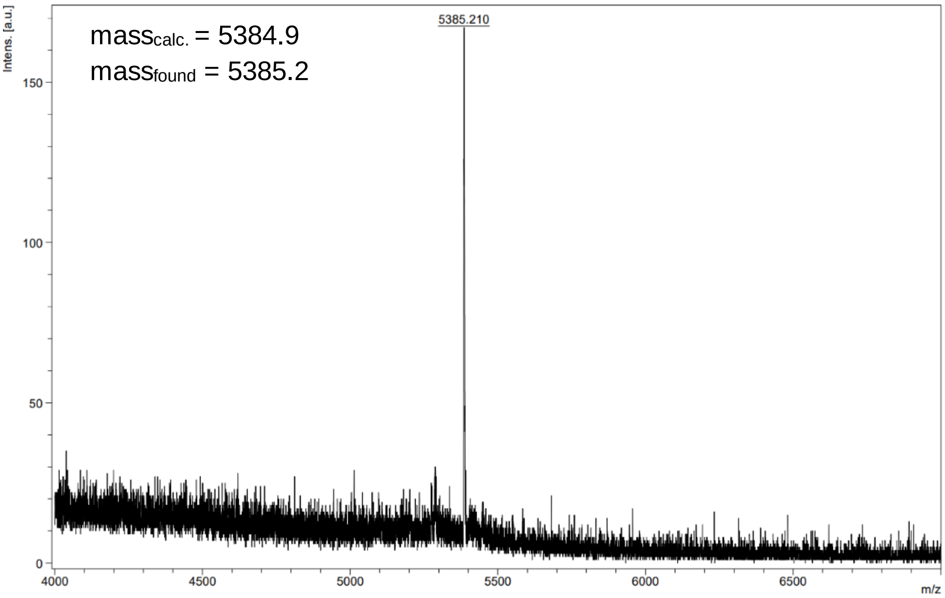

Ugi-azide three-component reaction

DNA conjugate 20: CPG-bound 16mer 7De-dAT7De8a-dGC-aldehyde conjugate **13** was reacted with piperidine **18**, *tert*-butylisocyanide **16** and trimethylsilyl azide **19** according to RP-06.

HPLC trace of crude reaction mixture **20** (analytical RP-HPLC (I))

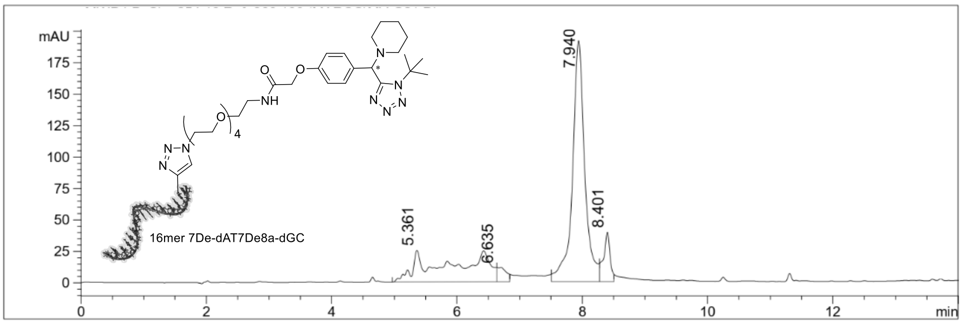

Peak list:

Ret. Time	Width min	Height	Area	Area %
5.361	0.800	25.038	1201.575	28.507
6.635	0.171	11.319	115.857	2.749
7.940	0.227	191.513	2611.569	61.959
8.401	0.121	39.317	285.990	6.785

HPLC trace of isolated product **20** (analytical RP-HPLC (I))

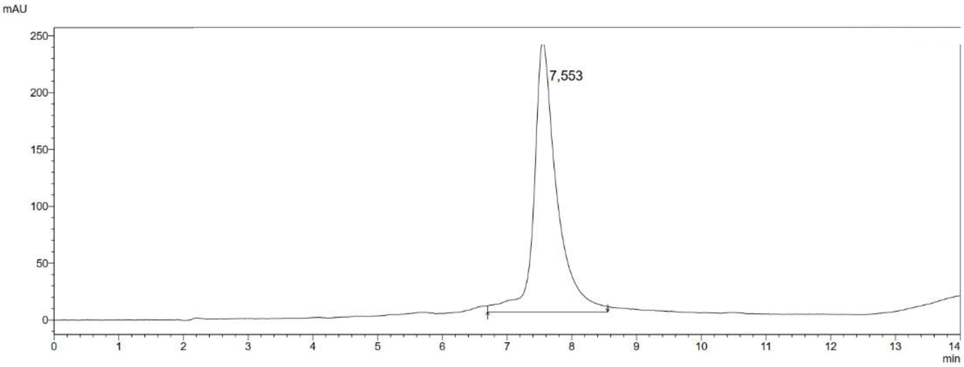

Peak Table

PDA Ch1 254nm

Peak#	Ret. Time	Area	Area%	Height	Height%
1	7,553	5792467	100,000	237672	100,000
Total		5792467	100,000	237672	100,000

MALDI-MS spectrum of isolated product **20**

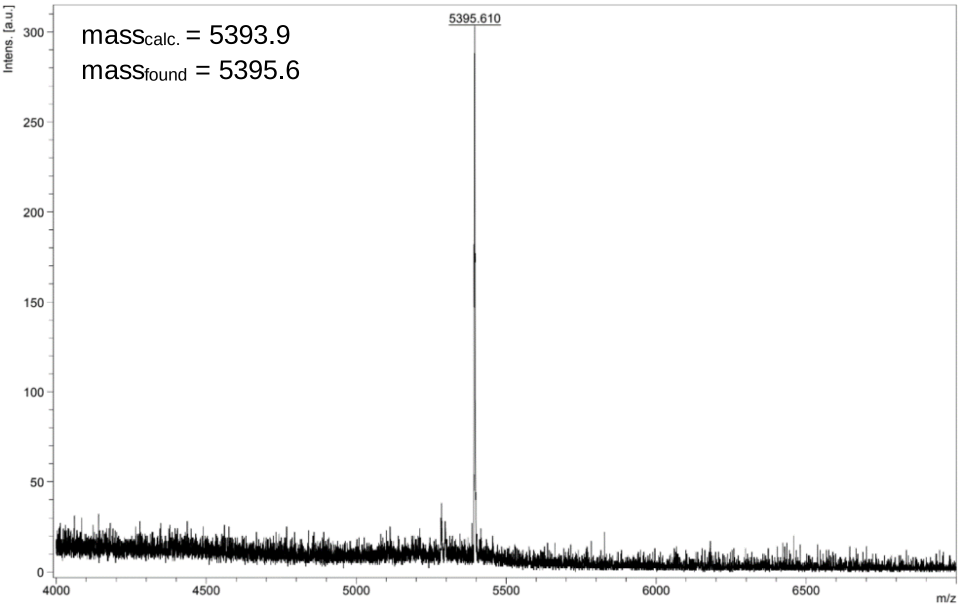

Groebke-Blackburn-Bienyamé three-component reaction

DNA conjugate 22: CPG-bound 16mer 7De-dAT7De8a-dGC-aldehyde conjugate **13** was reacted with 2-aminopyridine **21** and *tert*-butylisocyanide **16** according to RP-07.

HPLC trace of crude reaction mixture **22** (analytical RP-HPLC (I))

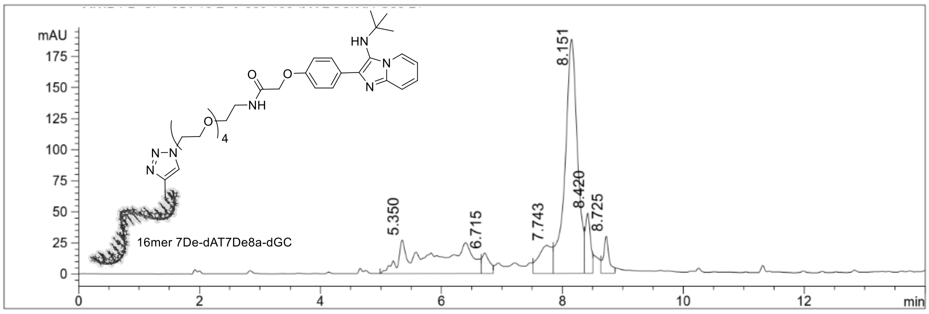


```
Peak list:

Ret. Time   Width min   Height     Area       Area %
----------------------------------------------------------
5.350       0.822       26.890     1327.023   26.754
6.715       0.140       16.366     137.710    2.776
7.743       0.260       22.516     351.008    7.077
8.151       0.236       188.208    2662.793   53.685
8.420       0.099       48.524     289.419    5.835
8.725       0.107       29.849     192.109    3.873
----------------------------------------------------------
```

HPLC trace of isolated product **22** (analytical RP-HPLC (I))

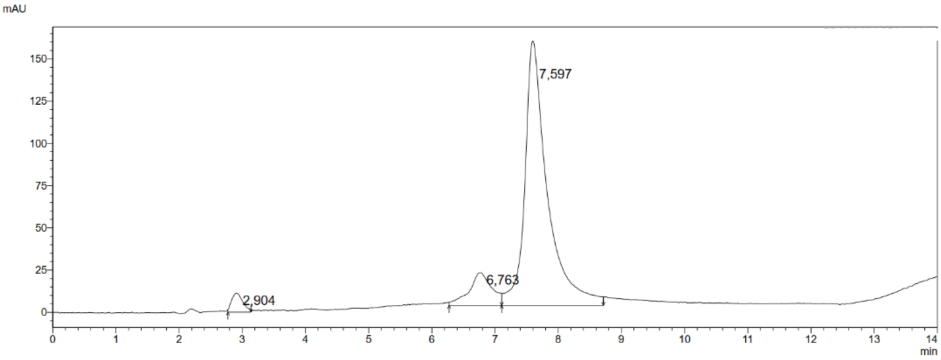

Peak Table

PDA Ch1 254nm

Peak#	Ret. Time	Area	Area%	Height	Height%
1	2,904	139025	3,092	11286	6,023
2	6,763	481465	10,709	19416	10,361
3	7,597	3875376	86,199	156686	83,616
Total		4495866	100,000	187388	100,000

MALDI-MS spectrum of isolated product **22**

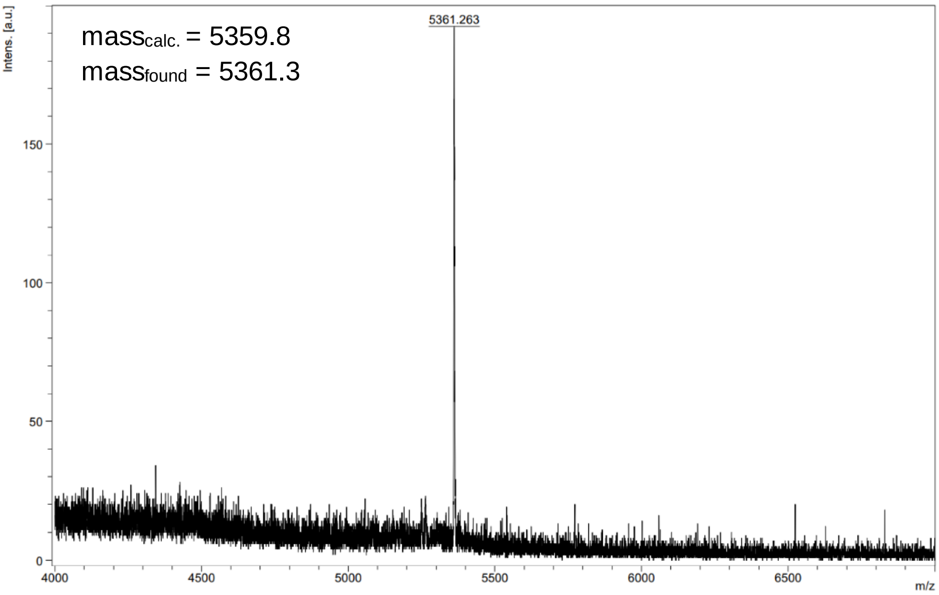

DNA conjugate 26: CPG-bound 16mer 7De-dAT7De8a-dGC-aldehyde conjugate **13** was reacted with benzoic acid **24**, *N*-Boc-piperazine **23** and (isocyanoimino)triphenylphosphorane **25** according to RP-08.

HPLC trace of crude reaction mixture **26** (analytical RP-HPLC (I))

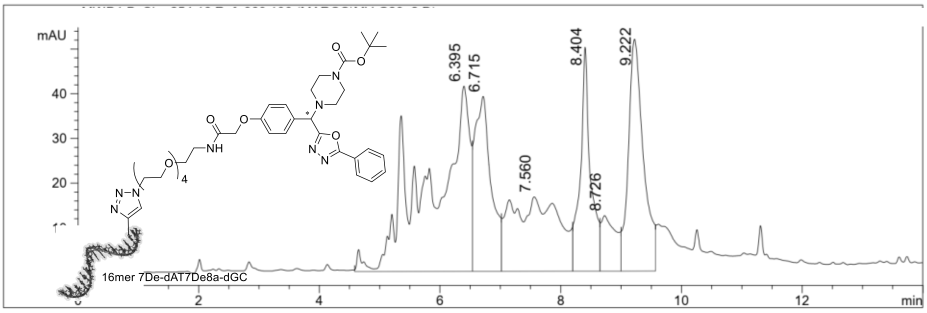

Peak list:

Ret. Time	Width min	Height	Area	Area %
6.395	0.736	41.488	1833.355	35.309
6.715	0.312	39.186	734.137	14.139
7.560	0.919	16.648	917.601	17.672
8.404	0.198	50.224	595.176	11.463
8.726	0.288	12.436	214.746	4.136
9.222	0.288	51.908	897.253	17.281

HPLC trace of isolated product **26** (analytical RP-HPLC (II))

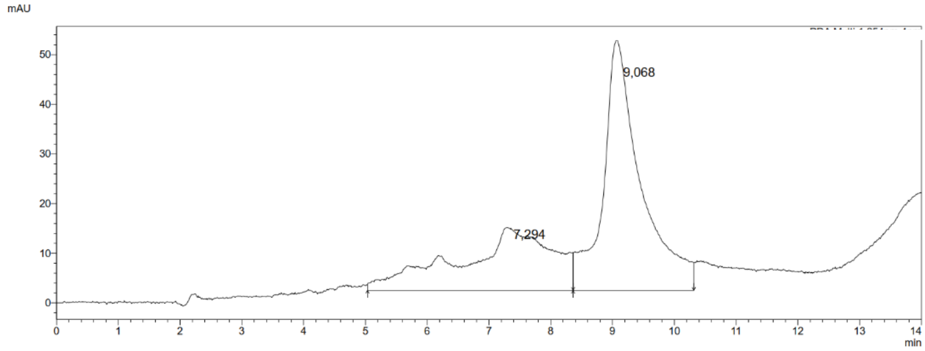

Peak Table

PDA Ch1 254nm

Peak#	Ret. Time	Area	Area%	Height	Height%
1	7,294	1302212	37,921	12688	20,078
2	9,068	2131773	62,079	50505	79,922
Total		3433985	100,000	63193	100,000

MALDI-MS spectrum of isolated product **26**

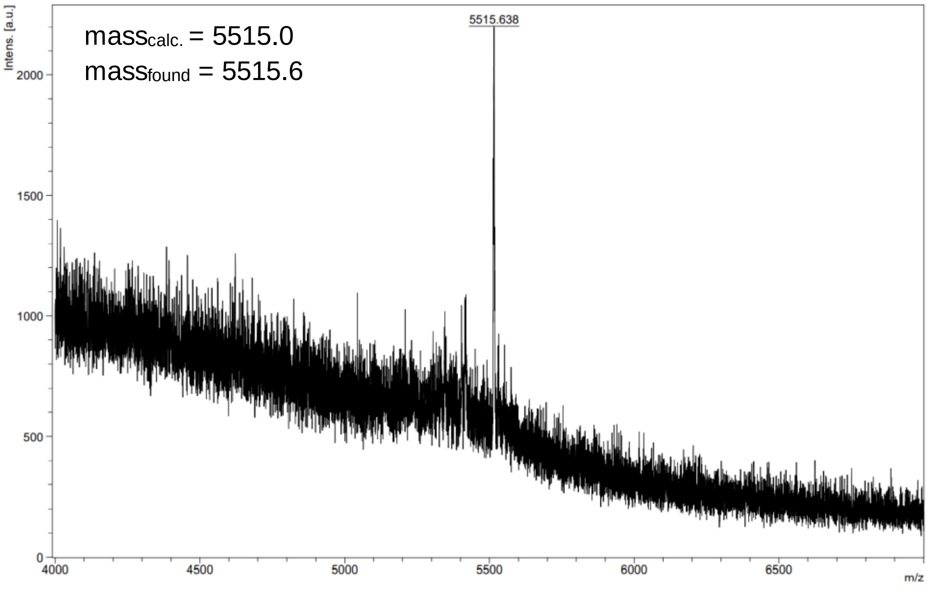

Brønsted acid-mediated reactions
Biginelli reaction

DNA conjugate 29: CPG-bound 16mer 7De-dAT7De8a-dGC-aldehyde conjugate **13** was reacted with urea **27,** and ethyl acetoacetate **28** according to RP-09.

HPLC trace of crude reaction mixture **29** (analytical RP-HPLC (**I**))

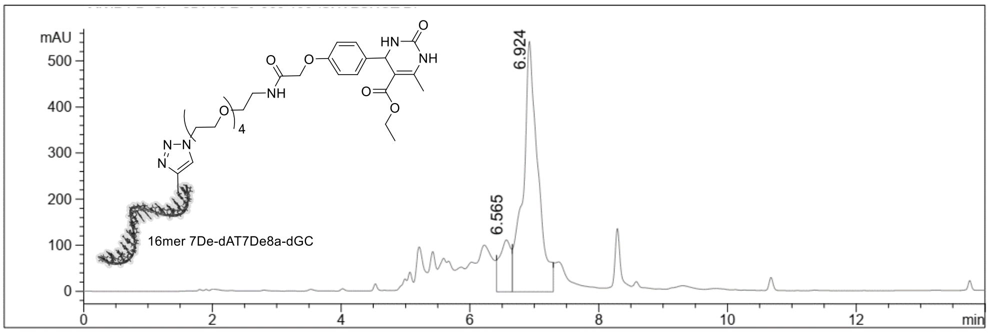


```
Peak list:

Ret. Time    Width min    Height      Area        Area %
---------------------------------------------------------
6.565        0.204        112.473     1378.739    13.908
6.924        0.262        543.819     8534.291    86.092
---------------------------------------------------------
```

HPLC trace of isolated product **29** (analytical RP-HPLC (**II**))

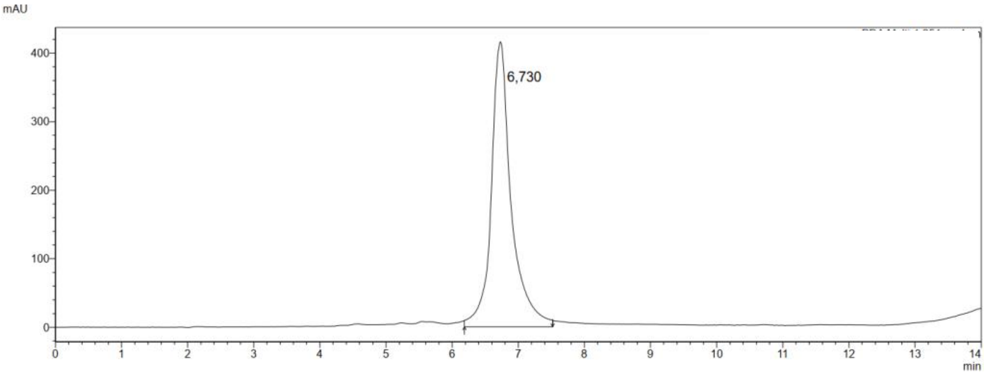

MALDI-MS spectrum of isolated product **29**

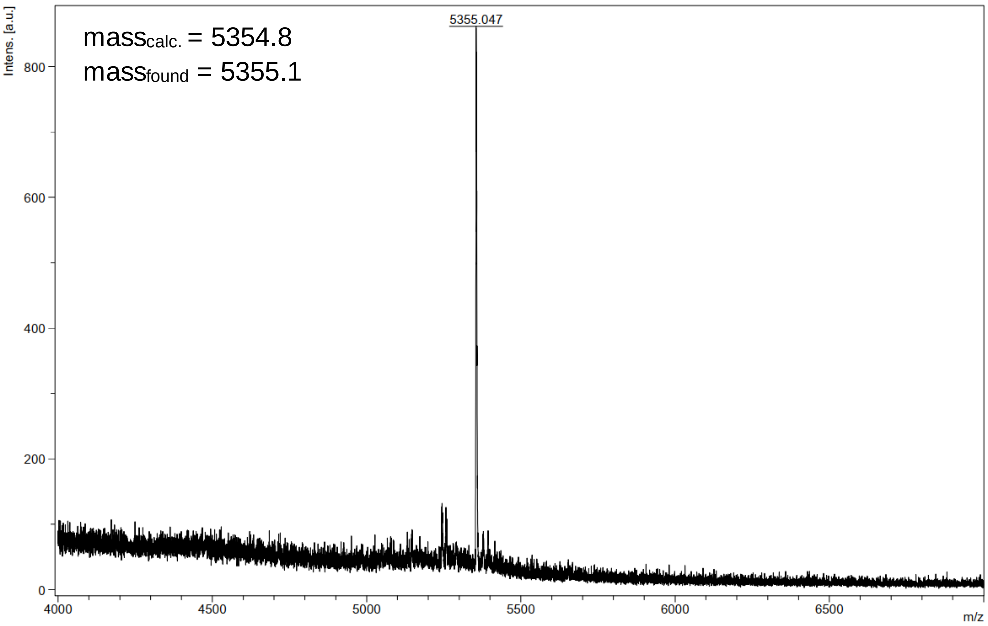

DNA conjugate 29a: CPG-bound 16mer 7De-dAT7De8a-dGC-aldehyde conjugate **13** was reacted with phenylurea**,** and ethyl acetoacetate **28** according to RP-09 with 200 equivalents of (*R*)-BNDHP.

HPLC trace of crude reaction mixture **29a** (analytical RP-HPLC (**I**))

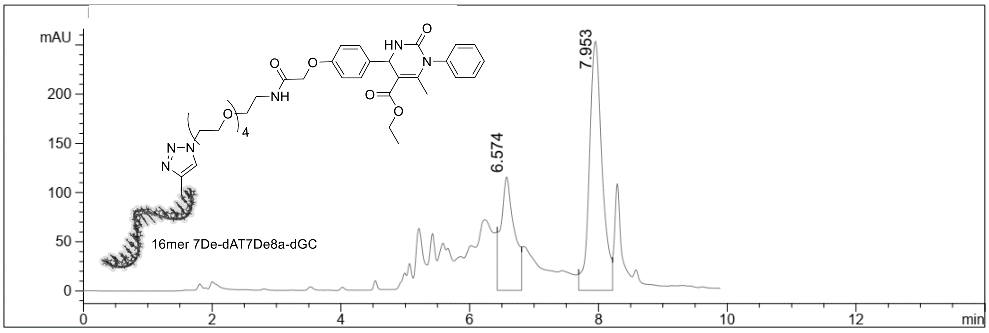

Peak list:

Ret. Time	Width min	Height	Area	Area %
6.574	0.241	115.177	1665.489	32.653
7.953	0.226	253.170	3435.080	67.347

HPLC trace of isolated product **29a** (analytical RP-HPLC (**II**))

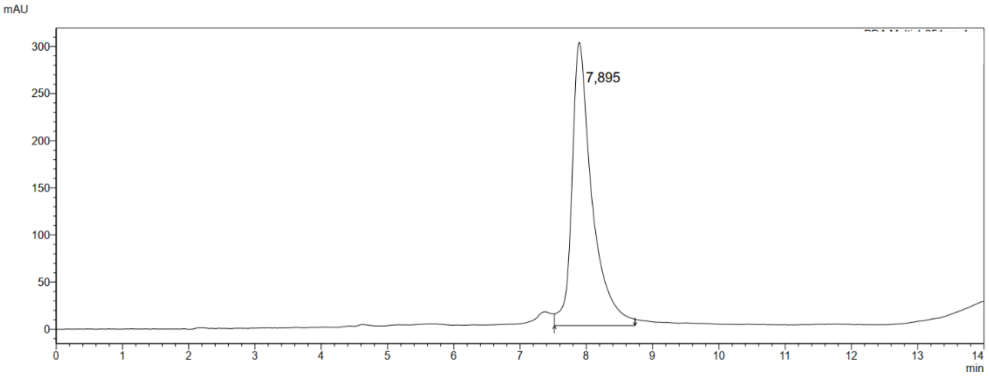

MALDI-MS spectrum of isolated product **29a**

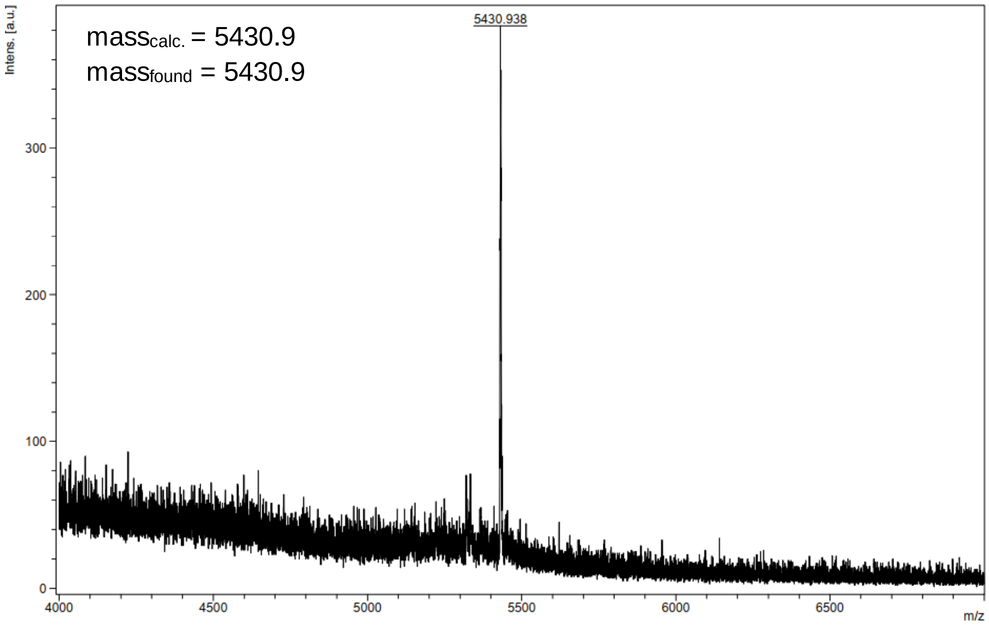

Povarov reaction

DNA conjugate 32: CPG-bound 16mer 7De-dAT7De8a-dGC-aldehyde conjugate **13** was reacted with aniline **30,** and *N*-Boc-2,3-dihydro-1*H*-pyrrole **31** according to RP-10.

HPLC trace of crude reaction mixture **32** (analytical RP-HPLC (II))

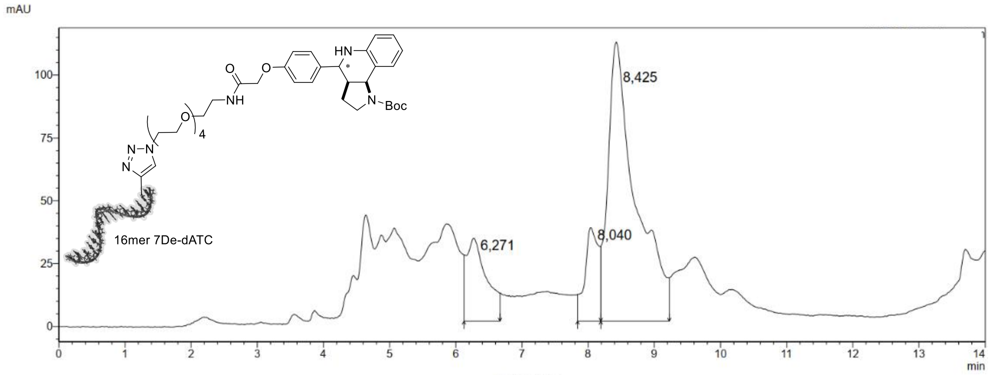

Peak Table

PDA Ch1 254nm

Peak#	Ret. Time	Area	Area%	Height	Height%
1	6,271	704153	15,749	33082	18,241
2	8,040	540867	12,097	37240	20,533
3	8,425	3226158	72,155	111044	61,226
Total		4471178	100,000	181366	100,000

HPLC trace of isolated product **32** (analytical RP-HPLC (II))

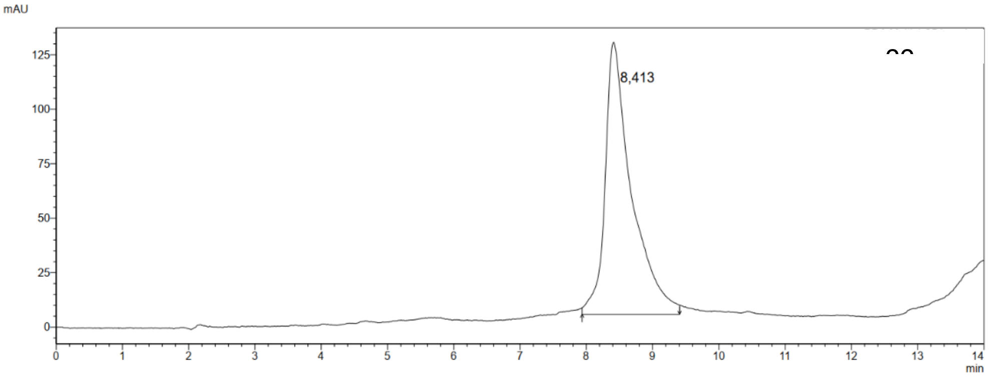

MALDI-MS spectrum of isolated product $\mathbf{32}_{\mathbf{dia1}}$

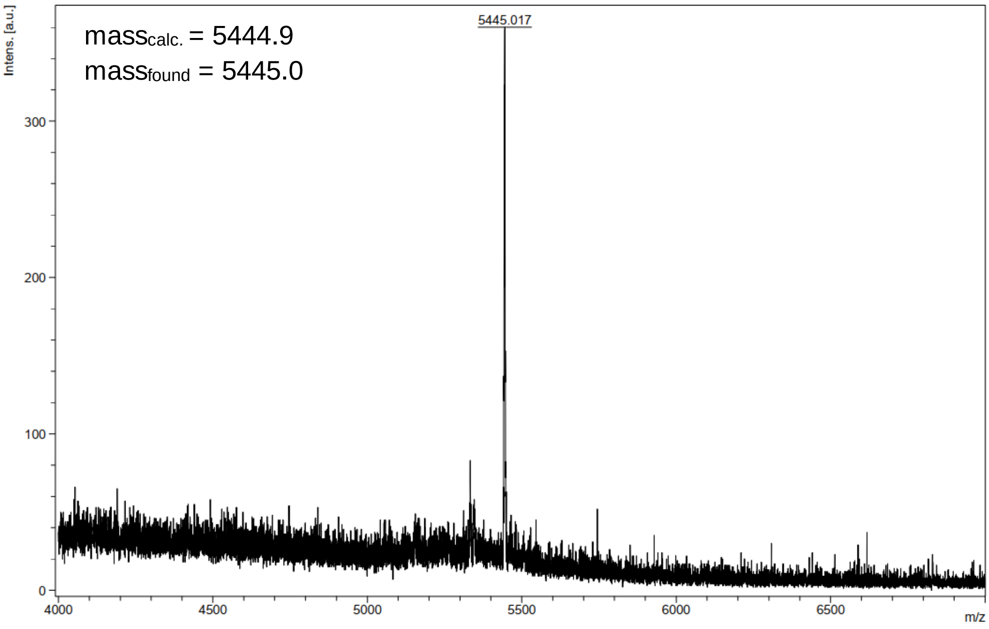

Pictet-Spengler reaction

DNA conjugate 35: CPG-bound 16mer 7De-dAT7De8a-dGC-tryptophan conjugate **33** was reacted with benzaldehyde **34** according to RP-11.

HPLC trace of crude reaction mixture **35** (analytical RP-HPLC (I))

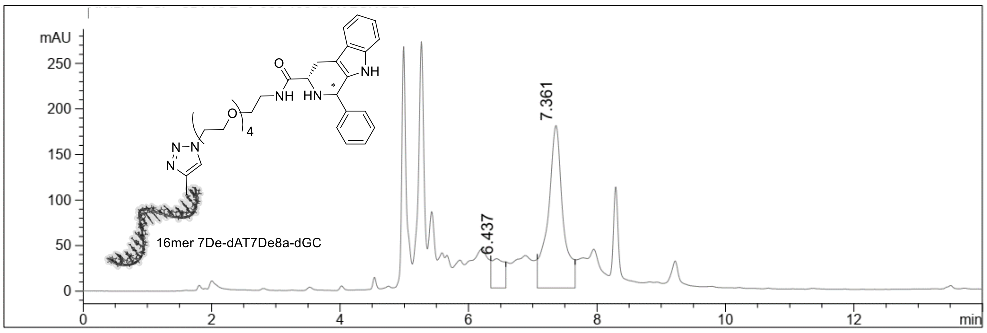

Peak list:

Ret. Time	Width min	Height	Area	Area %
6.437	0.220	32.250	425.207	13.027
7.361	0.265	178.428	2838.747	86.973

HPLC trace of isolated product **35** (analytical RP-HPLC (II))

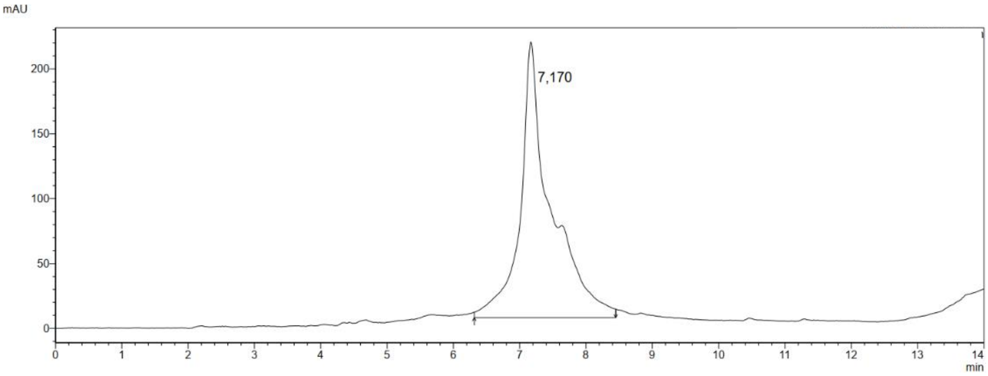

MALDI-MS spectrum of isolated product **35**

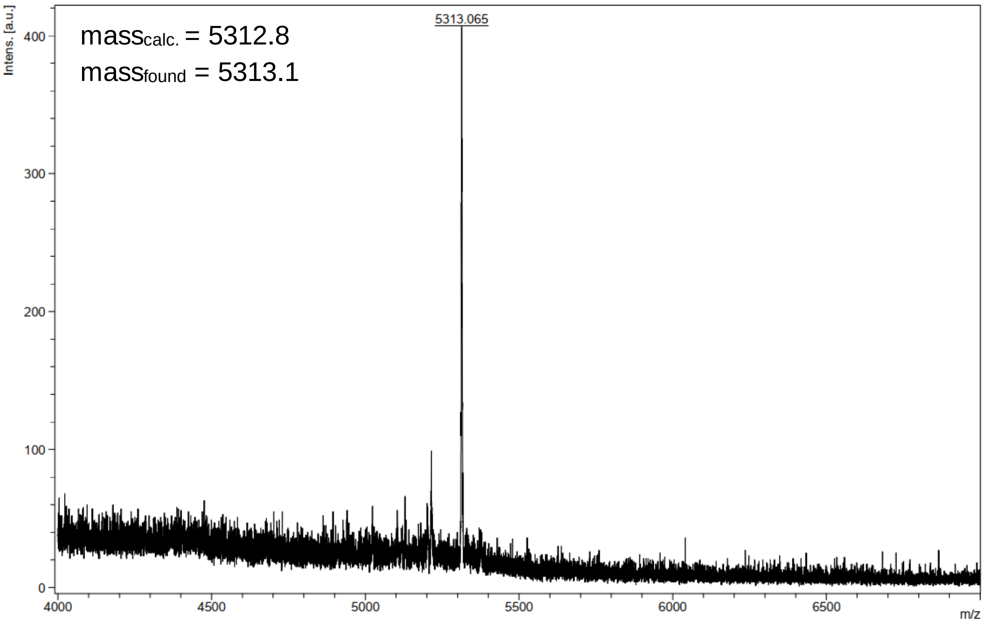

Lewis acid-promoted reactions
Petasis reaction

DNA conjugate 39: CPG-bound 16mer 7De-dAT7De8a-dGC-piperidine conjugate **36** was reacted with phenylboronic acid **38** and glyoxylic acid monohydrate **37** according to RP-12.

HPLC trace of crude reaction mixture **39** (analytical RP-HPLC (I))

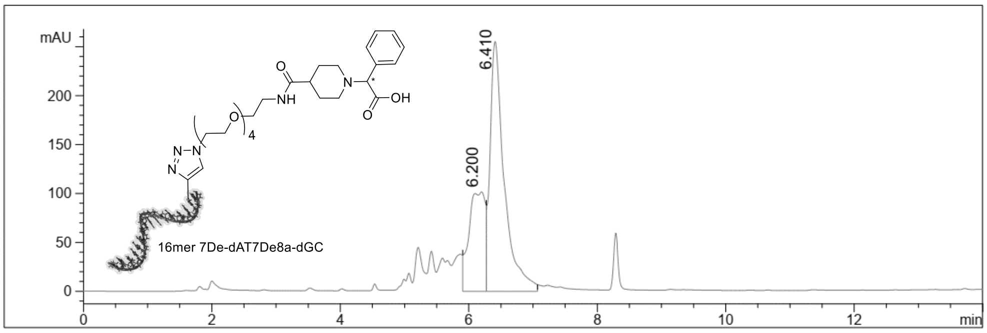


```
Peak list:

Ret. Time   Width min   Height     Area       Area %
-------------------------------------------------------
6.200       0.293       101.582    1788.189   31.347
6.410       0.256       255.414    3916.385   68.653
-------------------------------------------------------
```

HPLC trace of isolated product **39** (analytical RP-HPLC (II))

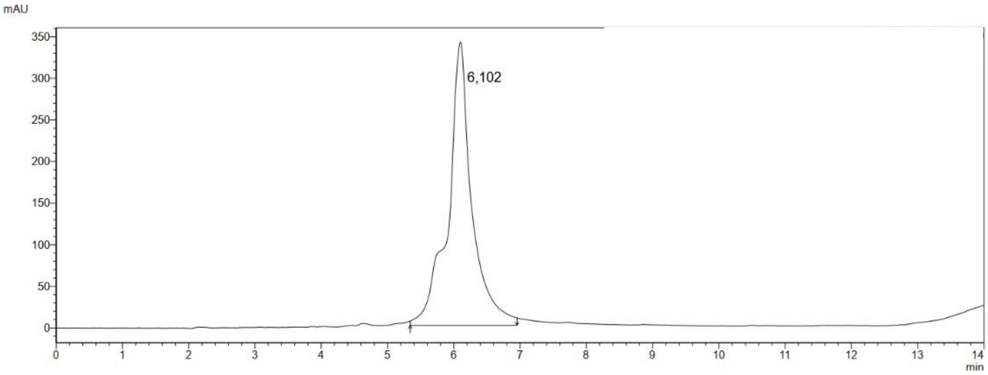

MALDI-MS spectrum of isolated product **39**

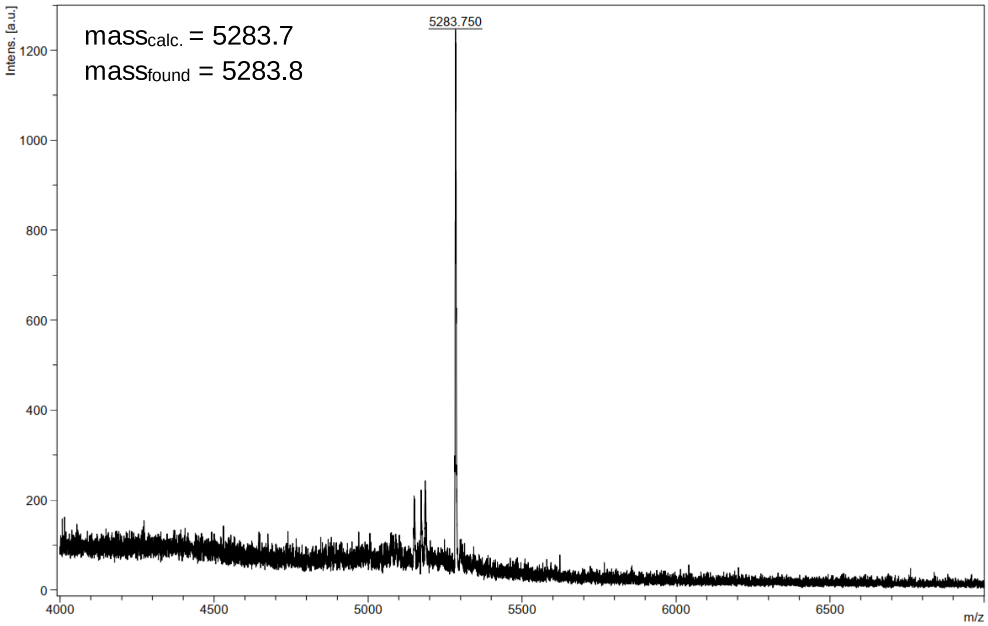

aza-Diels-Alder reaction

DNA conjugate 41: CPG-bound 16mer 7De-dAT7De8a-dGC-aldehyde conjugate **13** was reacted with aniline **30** and danishefsky's diene **40** according to RP-13.

HPLC trace of crude reaction mixture **41** (analytical RP-HPLC (I))

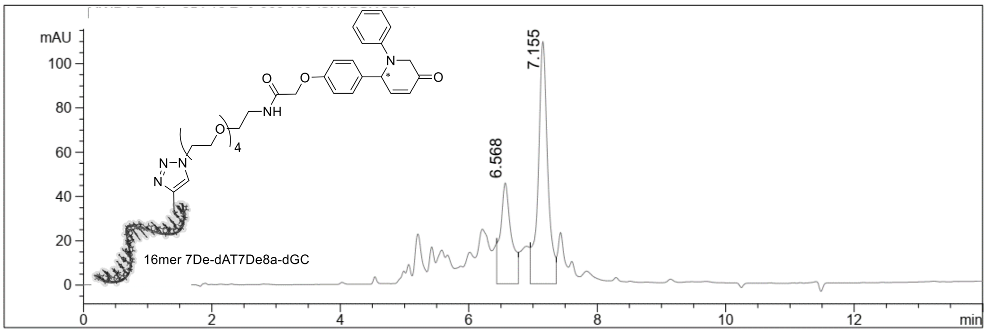

Peak list:

Ret. Time	Width min	Height	Area	Area %
6.568	0.195	45.576	534.402	32.306
7.155	0.171	109.428	1119.782	67.694

HPLC trace of isolated product **41** (analytical RP-HPLC (II))

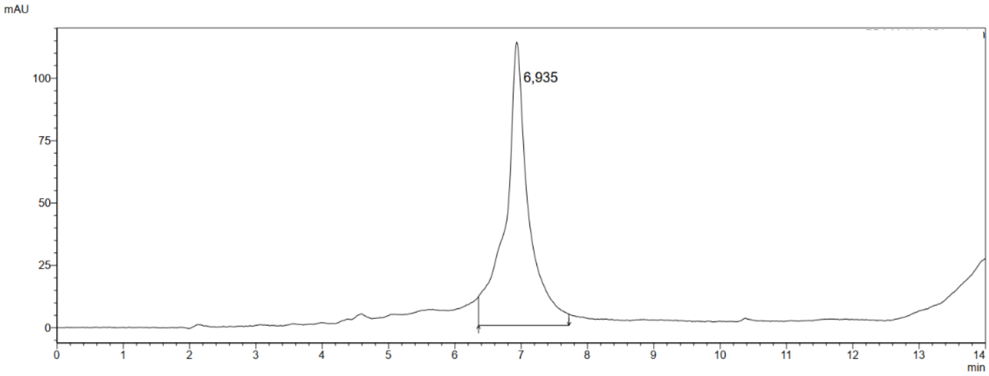

MALDI-MS spectrum of isolated product **41**

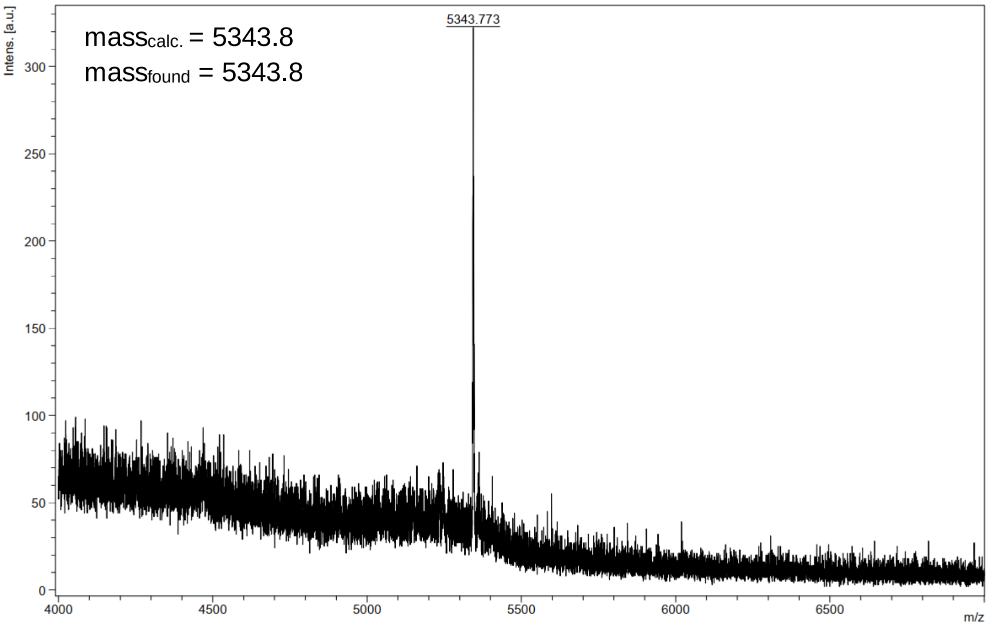

Castagnoli-Cushman reaction

DNA conjugate 43: CPG-bound 16mer 7De-dAT7De8a-dGC-aldehyde conjugate **13** was reacted with aniline **30** and homophthalic anhydride **42** according to RP-14.

HPLC trace of crude reaction mixture **43** (analytical RP-HPLC (I))

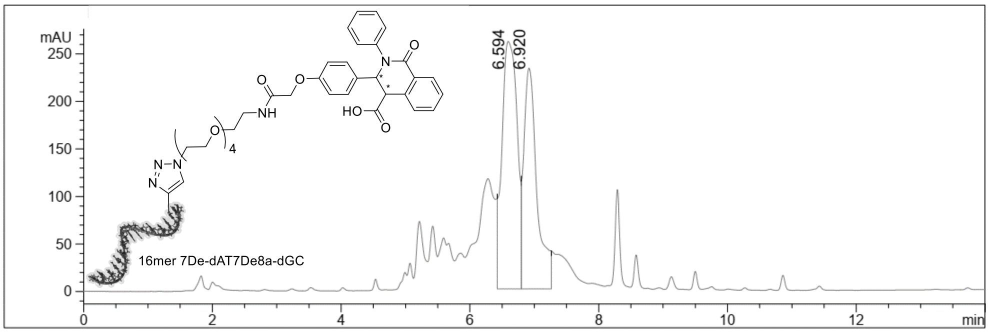

Peak list:

Ret. Time	Width min	Height	Area	Area %
6.594	0.273	260.515	4274.831	56.510
6.920	0.236	232.599	3289.840	43.490

HPLC trace of isolated product **43** (analytical RP-HPLC (II))

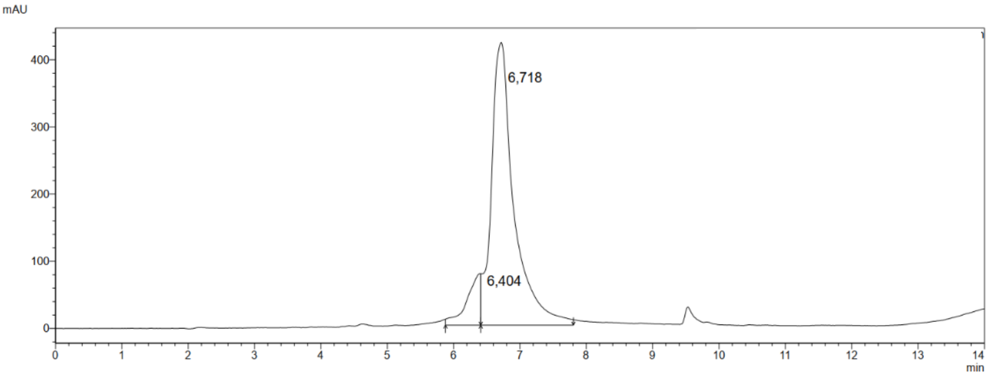

MALDI-MS spectrum of isolated product **43**

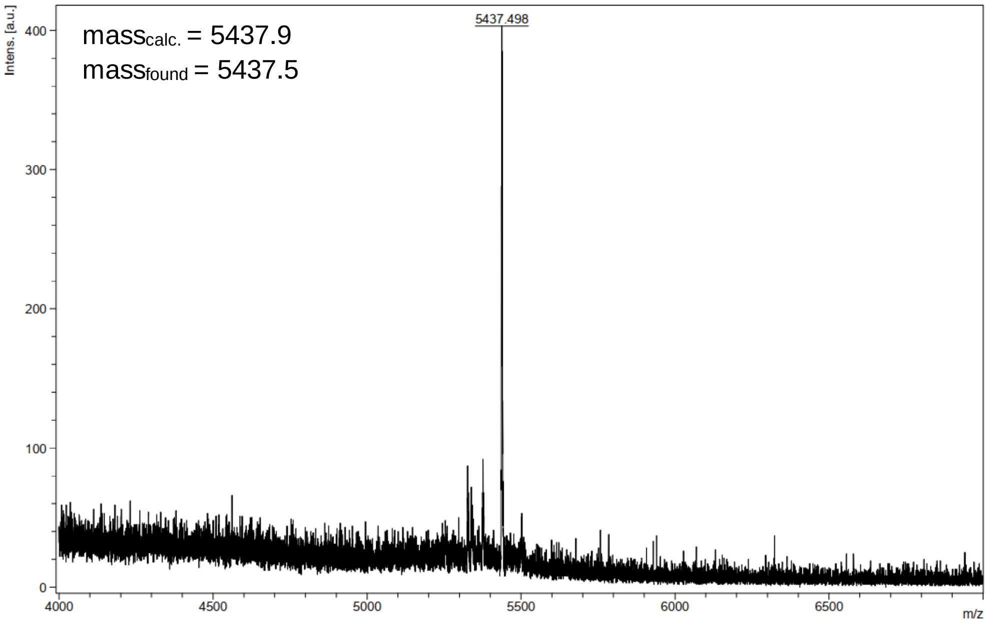

Yb(PFO)$_3$-mediated three-component synthesis of pyrazoles

DNA conjugate 45: CPG-bound 16mer 7De-dAT7De8a-dGC-aldehyde conjugate **13** was reacted with phenylhydrazine **44** and ethyl acetoacetate **28** according to RP-15.

HPLC trace of crude reaction mixture **45** (analytical RP-HPLC (I))

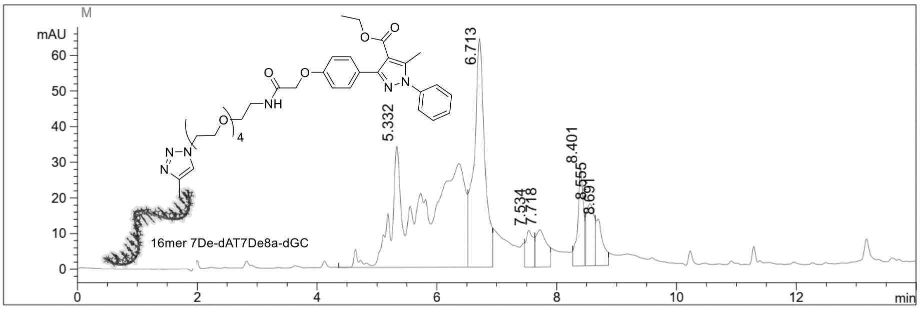

Peak list:

Ret. Time	Width min	Height	Area	Area %
5.332	0.816	34.005	1665.520	52.992
6.713	0.210	64.148	809.037	25.741
7.534	0.146	10.303	90.337	2.874
7.718	0.204	10.423	127.884	4.069
8.401	0.111	27.518	183.331	5.833
8.555	0.154	17.360	160.452	5.105
8.691	0.136	13.061	106.389	3.385

HPLC trace of isolated product **45** (analytical RP-HPLC (II))

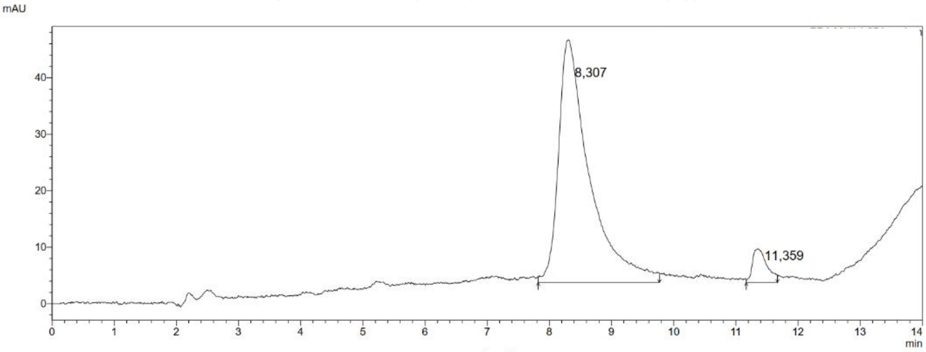

Peak Table

PDA Ch1 254nm

Peak#	Ret. Time	Area	Area%	Height	Height%
1	8,307	1490911	93,842	42963	87,814
2	11,359	97838	6,158	5962	12,186
Total		1588749	100,000	48926	100,000

MALDI-MS spectrum of isolated product **45**

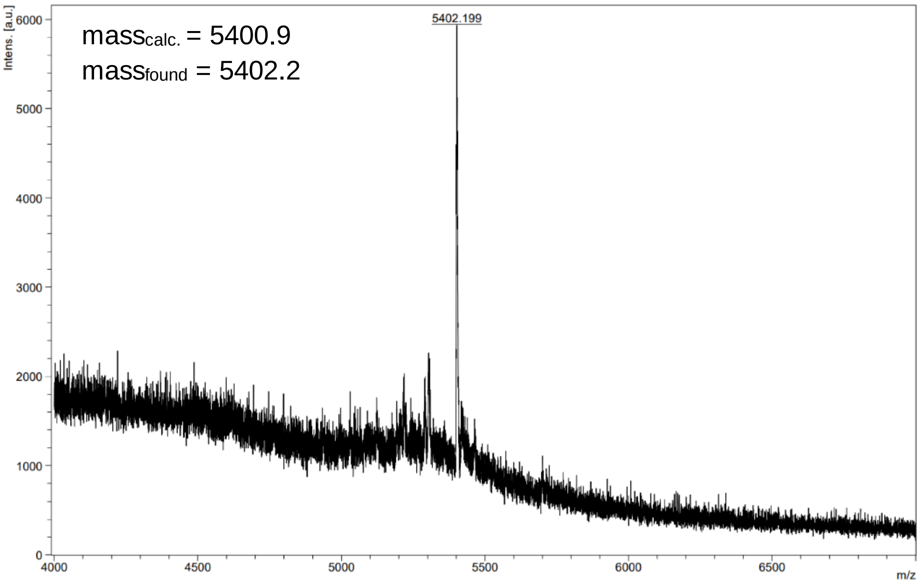

Au(I)/Ag(I)-promoted pyrazoline-containing spiroheterocycle synthesis

DNA conjugate 48: CPG-bound 16mer 7De-dAT7De8a-dGC-aldehyde conjugate **13** was reacted with pent-4-yn-1-ol **46** and *tert*-butyl 2-benzylhydrazine-carboxylate **47** according to RP-16.

HPLC trace of crude reaction mixture **48** (analytical RP-HPLC (I))

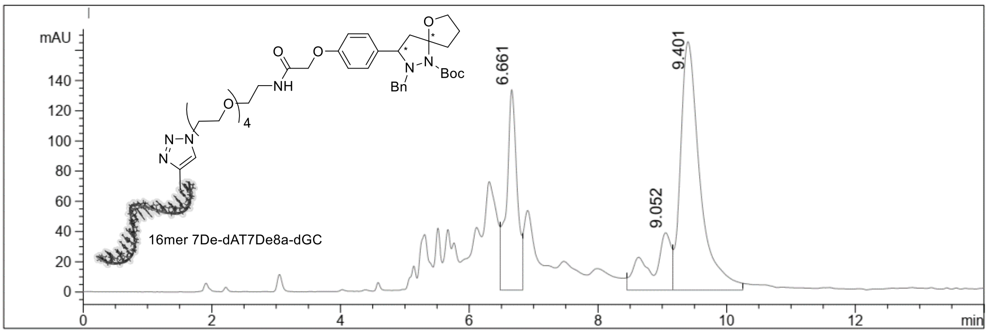

Peak list:

Ret. Time	Width min	Height	Area	Area %
6.661	0.191	132.812	1518.079	26.172
9.052	0.372	37.919	845.359	14.574
9.401	0.348	164.433	3436.979	59.254

HPLC trace of isolated product **48** (analytical RP-HPLC (II))

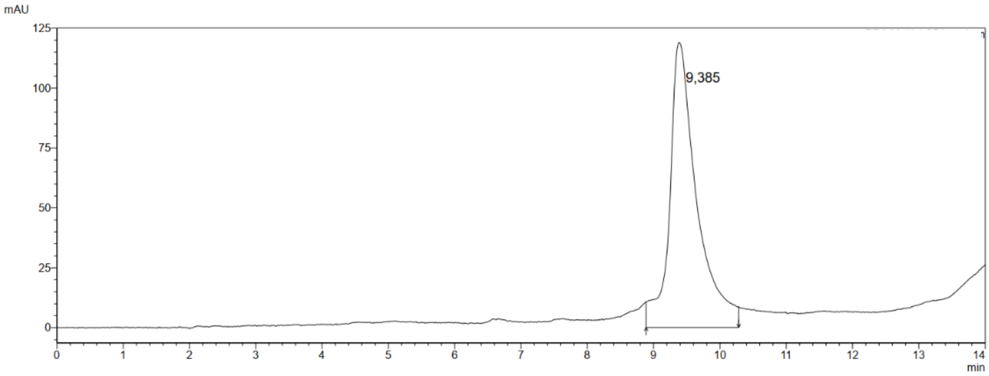

MALDI-MS spectrum of isolated product **48**

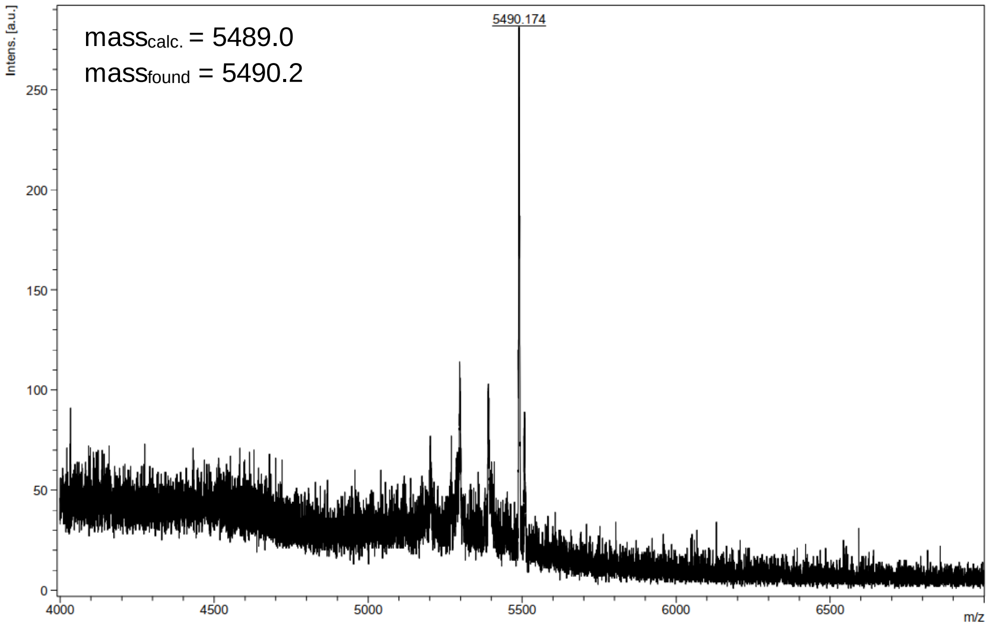

Au(I)/Ag(I)-promoted pyrazoline synthesis

DNA conjugate 51: CPG-bound 16mer 7De-dAT7De8a-dGC-alkyne conjugate **49** was reacted with isobutyraldehyde **50** and *tert*-butyl 2-benzylhydrazine-carboxylate **47** according to RP-17.

HPLC trace of crude reaction mixture **51** (analytical RP-HPLC (I))

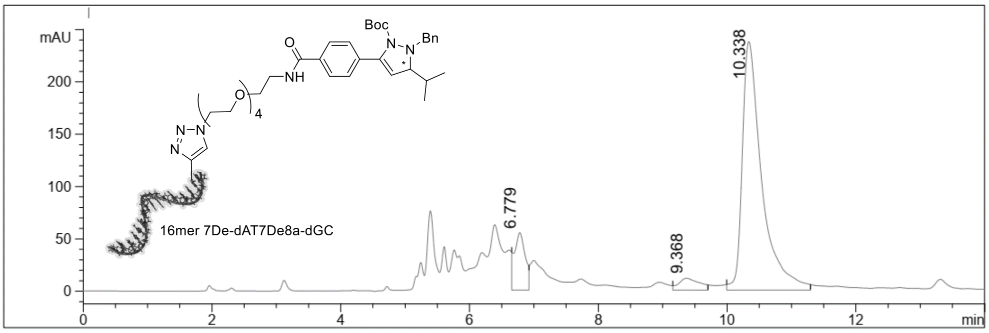

Peak list:

Ret. Time	Width min	Height	Area	Area %
6.779	0.193	54.706	632.944	10.807
9.368	0.374	11.260	252.439	4.310
10.338	0.349	237.392	4971.494	84.883

HPLC trace of isolated product **51** (analytical RP-HPLC (II))

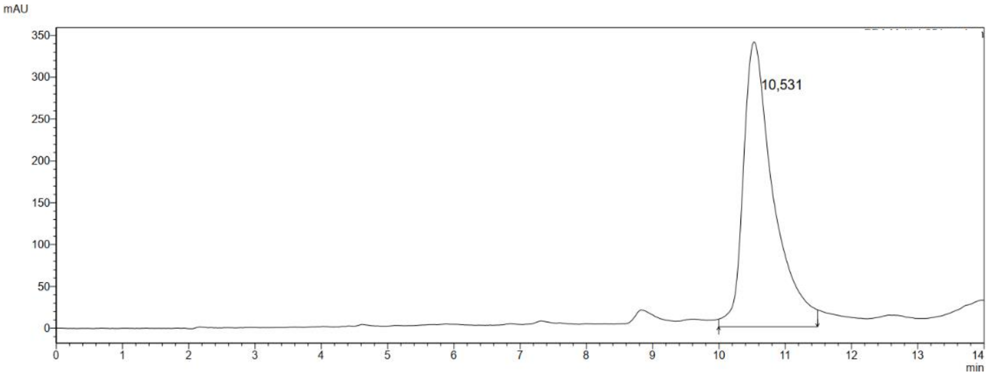

MALDI-MS spectrum of isolated product **51**

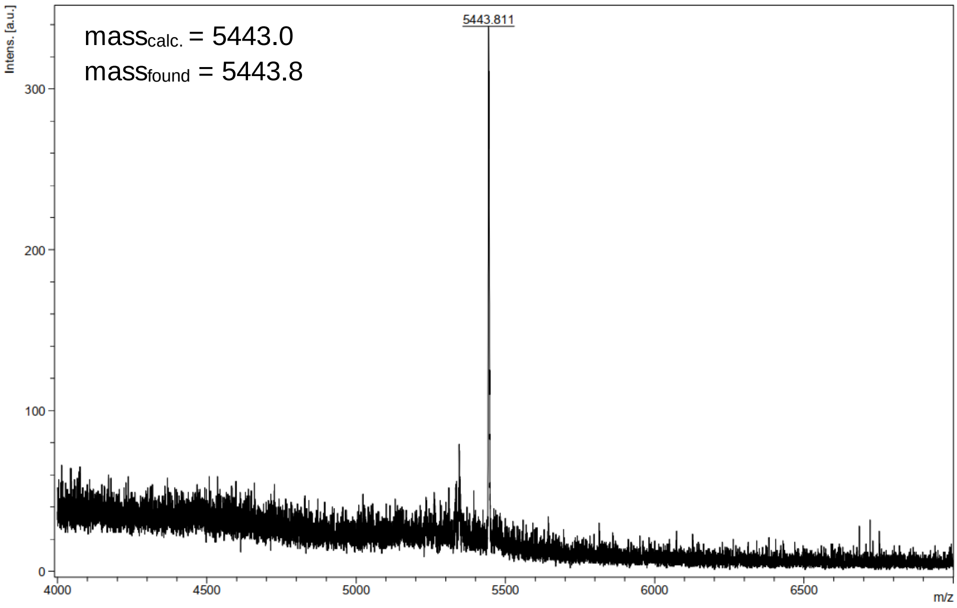

Au(I)/Ag(I)-promoted pyrazole synthesis

DNA conjugate 52: CPG-bound 16mer 7De-dAT7De8a-dGC-alkyne conjugate **49** was reacted with benzaldehyde **34** and *tert*-butyl 2-benzylhydrazine-carboxylate **37** according to RP-18.

HPLC trace of crude reaction mixture **52** (analytical RP-HPLC (I))

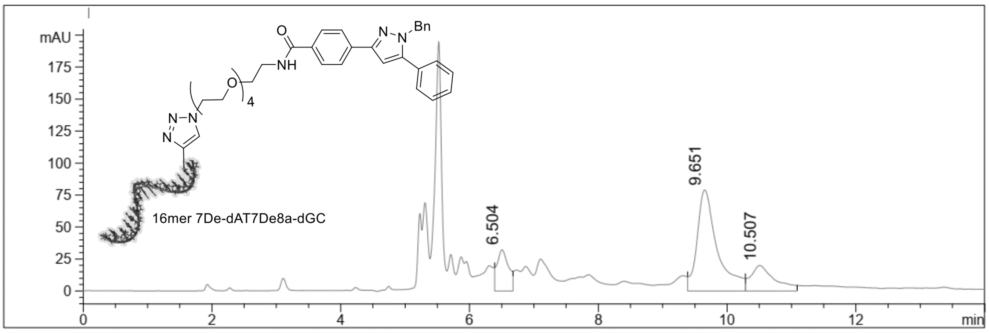

Peak list:

Ret. Time	Width min	Height	Area	Area %
6.504	0.206	32.204	397.243	15.121
9.651	0.362	78.979	1713.328	65.218
10.507	0.432	19.921	516.518	19.661

HPLC trace of isolated product **52** (analytical RP-HPLC (II))

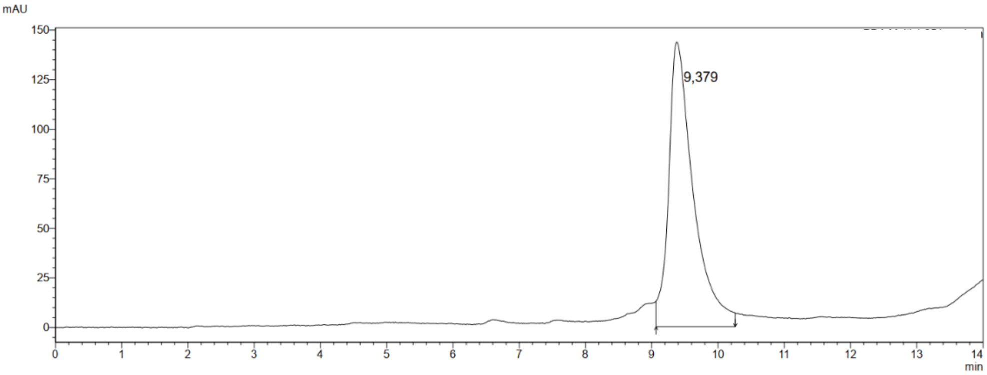

MALDI-MS spectrum of isolated product **52**

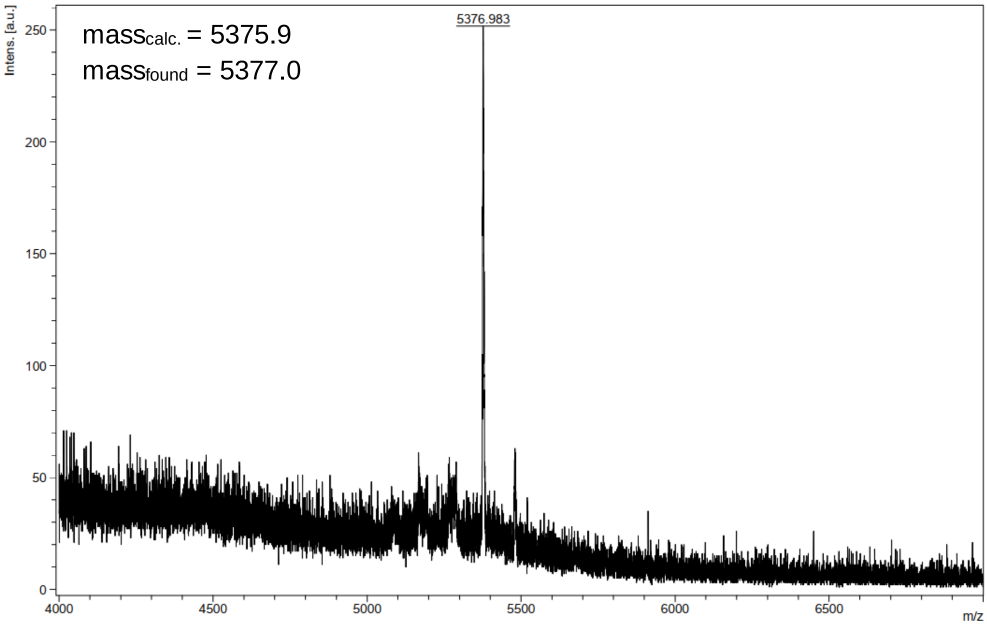

Boc cleavage in aqueous solution

DNA conjugate 53: CPG-bound 16mer 7De-dAT7De8a-dGC-Povarov conjugate **32** was Boc-deprotected according to RP-19.

HPLC trace of purified 16mer 7De-dAT7De8a-dGC-Povarov conjugate **32** (analytical RP-HPLC (II))

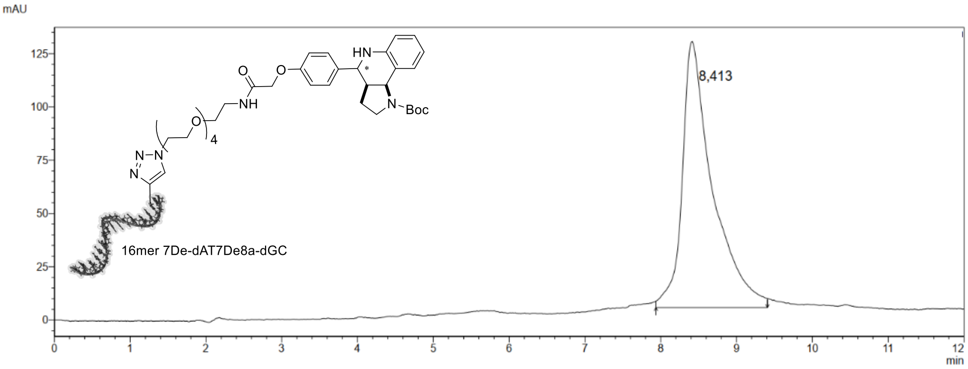

Peak Table

PDA Ch1 254nm

Peak#	Ret. Time	Area	Area%	Height	Height%
1	8,413	3467904	100,000	124909	100,000
Total		3467904	100,000	124909	100,000

HPLC trace of Boc-deprotected 16mer 7De-dAT7De8a-dGC-Povarov conjugate **53** (analytical RP-HPLC (II))

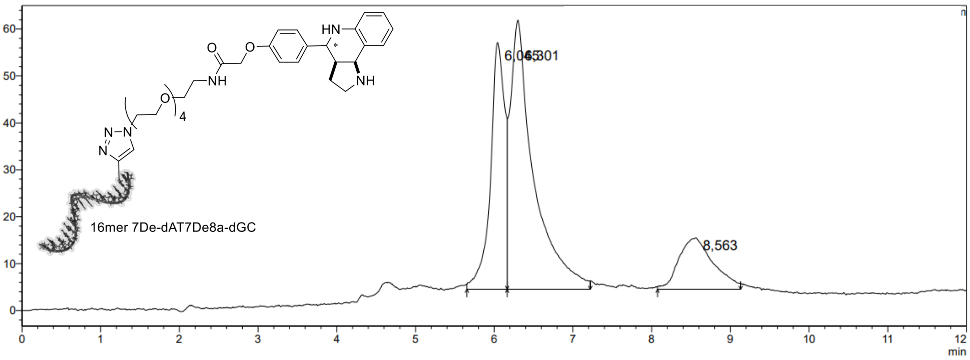

Peak Table

PDA Ch1 254nm

Peak#	Ret. Time	Area	Area%	Height	Height%
1	6,045	674636	30,864	52610	43,514
2	6,301	1178597	53,919	57343	47,429
3	8,563	332618	15,217	10949	9,056
Total		2185851	100,000	120902	100,000

MALDI-MS spectrum of isolated product **53**

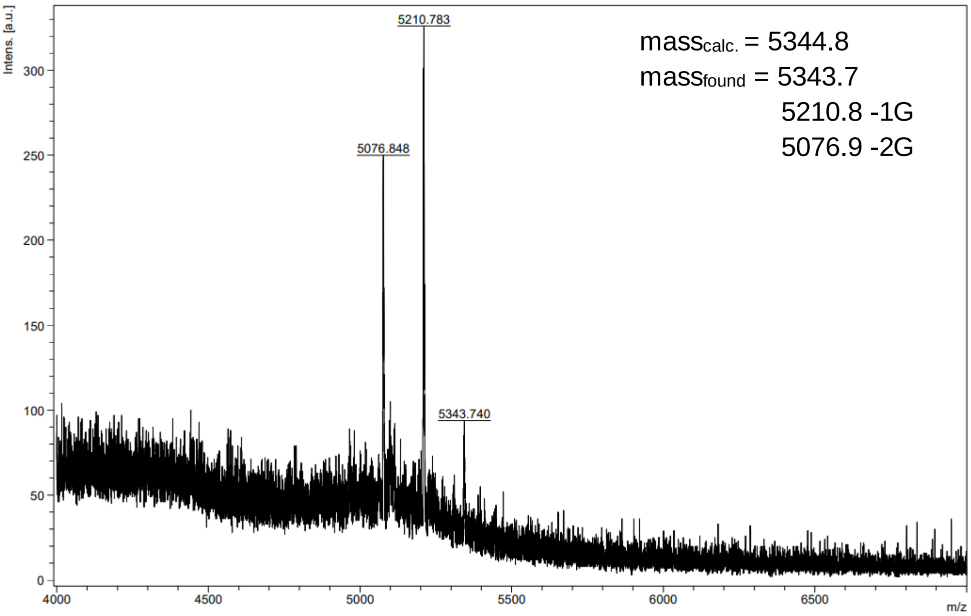

References

[1] M. Potowski, V. B. K. Kunig, L. Eberlein, A. Vakalopoulos, S. M. Kast, A. Brunschweiger, *Angew. Chem*. **2021**, 60, 19744-19749.

[2] L. Eberlein, F. R. Beierlein, N. J. R. van Eikema Hommes, A. Radadiya, J. Heil, S. A. Benner, T. Clark, S. M. Kast, N. G. J. Richards, *J. Chem. Theory Comput*. **2020**, 16, 2766-2777.

[3] M. J. Frisch, G. W. Trucks, H. B. Schlegel, G. E. Scuseria, M. A. Robb, J. R. Cheeseman, G. Scalmani, V. Barone, G. A. Petersson, H. Nakatsuji, X. Li, M. Caricato, A. V. Marenich, J. Bloino, B. G. Janesko, R. Gomperts, B. Mennucci, H. P. Hratchian, J. V. Ortiz, A. F. Izmaylov, J. L. Sonnenberg, D. Williams-Young, F. Ding, F. Lipparini, F. Egidi, J. Goings, B. Peng, A. Petrone, T. Henderson, D. Ranasinghe, V. G. Zakrzewski, J. Gao, N. Rega, G. Zheng, W. Liang, M. Hada, M. Ehara, K. Toyota, R. Fukuda, J. Hasegawa, M. Ishida, T. Nakajima, Y. Honda, O. Kitao, H. Nakai, T. Vreven, K. Throssell, J. A. Montgomery, Jr., J. E. Peralta, F. Ogliaro, M. J. Bearpark, J. J. Heyd, E. N. Brothers, K. N. Kudin, V. N. Staroverov, T. A. Keith, R. Kobayashi, J. Normand, K. Raghavachari, A. P. Rendell, J. C. Burant, S. S. Iyengar, J. Tomasi, M. Cossi, J. M. Millam, M. Klene, C. Adamo, R. Cammi, J. W. Ochterski, R. L. Martin, K. Morokuma, O. Farkas, J. B. Foresman, D. J. Fox, *Gaussian 16*, Re. B.01, Gaussian Inc., Wallingford CT, 2016.

[4] F. Neese, *WIREs Comput. Mol. Sci.* **2012**, 2, 73-78.

[5] F. Neese, *J. Comput. Chem*. **2003**, 24, 1740-1747.

[6] F. Pavošević, P. Pinski, C. Riplinger, F. Neese, E. F. Valeev, *J. Chem. Phys*. **2016**, 144, 144109.

[7] T. Kloss, J. Heil, S. M. Kast, *J. Phys. Chem. B* **2008**, 112, 4337-4343.

[8] T. Pongratz, P. Kibies, L. Eberlein, N. Tielker, C. Hölzl, S. Imoto, M. Beck Erlach, S. Kurrmann, P. H. Schummel, M. Hofmann, O. Reiser, R. Winter, W. Kremer, H. R. Kalbitzer, D. Marx, D. Horinek, S. M. Kast, *Biophys. Chem*. **2020**, 257, 106258.

[9] N. Tielker, L. Eberlein, S. Güssregen, S. M. Kast, *J. Comput.-Aided Mol. Des*. **2018**, 32, 1151-1163.

[10] S. M. Kast, T. Kloss, *J. Chem. Phys*. **2008**, 129, 236101.

[11] J. Wang, R. M. Wolf, J. W. Caldwell, P. A. Kollman, D. A. Case, *J. Comput. Chem.* **2004**, 25, 1157-1174.

[12] J. Wang, W. Wang, P. A. Kollman, D. A. Case, *J. Mol. Graph. Model*. **2006**, 25, 247-260.

[13] M. J. Frisch, G. W. Trucks, H. B. Schlegel, G. E. Scuseria, M. A. Robb, J. R. Cheeseman, G. Scalmani, V. Barone, B. Mennucci, G. A. Petersson, H. Nakatsuji, M. Caricato, X. Li, H. P. Hratchian, A. F. Izmaylov, J. Bloino, G. Zheng, J. L. Sonnenberg, M. Hada, M. Ehara, K. Toyota, R. Fukuda, J. Hasegawa, M. Ishida, T. Nakajima, Y. Honda, O. Kitao, H. Nakai, T. Vreven, J. A. Montgomery, Jr., J. E. Peralta, F. Ogliaro, M. Bearpark, J. J. Heyd, E. Brothers, K. N. Kudin, V. N. Staroverov, R. Kobayashi, J. Normand, K. Raghavachari, A. Rendell, J. C. Burant, S. S. Iyengar, J. Tomasi, M. Cossi, N. Rega, J. M. Millam, M. Klene, J. E. Knox, J. B. Cross, V. Bakken, C. Adamo, J. Jaramillo, R. Gomperts, R. E. Stratmann, O. Yazyev, A. J. Austin, R. Cammi, C. Pomelli, J. W. Ochterski, R. L. Martin, K. Morokuma, V. G. Zakrzewski, G. A. Voth, P. Salvador, J. J. Dannenberg, S. Dapprich, A. D. Daniels, Ö. Farkas, J. B. Foresman, J. V. Ortiz, J. Cioslowski, D. J. Fox, *Gaussian 09*, Rev. E.01, Gaussian Inc. Wallingford CT, 2009.

[14] H. J. C. Berendsen, J. R. Grigera, T. P. Straatsma, *J. Phys. Chem*. **1987**, 91, 6269-6271.

[15] L. Martínez, R. Andrade, E. G. Birgin, J. M. Martínez, *J. Comput. Chem*. **2009**, 30, 2157-2164.

[16] J. C. Phillips, R. Braun, W. Wang, J. Gumbart, E. Tajkhorshid, E. Villa, C. Chipot, R. D. Skeel, L. Kalé, K. Schulten, *J. Comput. Chem*. **2005**, 26, 1781-1802.

[17] F. Seela, H. Driller, *Helvetica Chimica Acta*, **1988**, *71*, 1191-1198.

[18] V. B. K. Kunig, C. Ehrt, A. Dömling, A. Brunschweiger, *Org. Lett.* **2019**, *21*, 7238-7243.

[19] M. Potowski, F. Losch, E. Wünnemann, J. K. Dahmen, S. Chines, A. Brunschweiger, *Chem. Sci.* **2019**, *10*, 10481-10492.

[20] M. Klika Škopić, H. Salamon, O. Bugain, K. Jung, A. Gohla, L. J. Doetsch, D. dos Santos, A. Bhat, B. Wagner, A. Brunschweiger, *Chem. Sci.* **2017**, *8*, 3356-3361.

[21] M. Potowski, V. B. K. Kunig, F. Losch, A. Brunschweiger, *Med. Chem. Commun.* **2019**, *10*, 1082-1093.

[22] M. Potowski, R. Esken, A. Brunschweiger, *Bioorg. Med. Chem.* **2020**, *28*, 115441.

[23] M Klika Škopić, S. Willems, B. Wagner, J. Schieven, N. Krause, A. Brunschweiger, *Org. Biomol. Chem.* **2017**, *15*, 8648–8654.
